# New Crystalline Salts of Nicotinamide Riboside as Food Additives

**DOI:** 10.3390/molecules26092729

**Published:** 2021-05-06

**Authors:** Günter Schabert, Robert Haase, Jaclyn Parris, Laura Pala, Adrian Hery-Barranco, Bernhard Spingler, Urs Spitz

**Affiliations:** 1 Biosynth Carbosynth, Rietlistrasse 4, 9422 Staad, Switzerland; guenter.schabert@biosynth-carbosynth.com (G.S.); Robert.Haase@biosynth-carbosynth.com (R.H.); 2 Department of Chemistry, University of Zurich, Winterthurerstrasse 190, 8057 Zurich, Switzerland; jaclyn.parris@uzh.ch (J.P.); spingler@chem.uzh.ch (B.S.); 3 Biosynth Carbosynth, Axis House, High Street, Compton, Berkshire RG20 6NL, UK; laura.pala@biosynth-carbosynth.com (L.P.); adrian.herybarranco@biosynth-carbosynth.com (A.H.-B.)

**Keywords:** nicotinamide riboside, B3 supplement, NR^+^ salts, crystal structures, salt screening

## Abstract

NR^+^ is a highly effective vitamin B_3_ type supplement due to its unique ability to replenish NAD^+^ levels. While NR^+^ chloride is already on the market as a nutritional supplement, its synthesis is challenging, expensive, and low yielding, making it cumbersome for large-scale industrial production. Here we report the novel crystalline NR^+^ salts, d/l/dl-hydrogen tartrate and d/l/dl-hydrogen malate. Their high-yielding, one-pot manufacture does not require specific equipment and is suitable for multi-ton scale production. These new NR^+^ salts seem ideal for nutritional applications due to their bio-equivalence compared to the approved NR^+^ chloride. In addition, the crystal structures of all stereoisomers of NR^+^ hydrogen tartrate and NR^+^ hydrogen malate and a comparison to the known NR^+^ halogenides are presented.

## 1. Introduction

**Figure 1:**
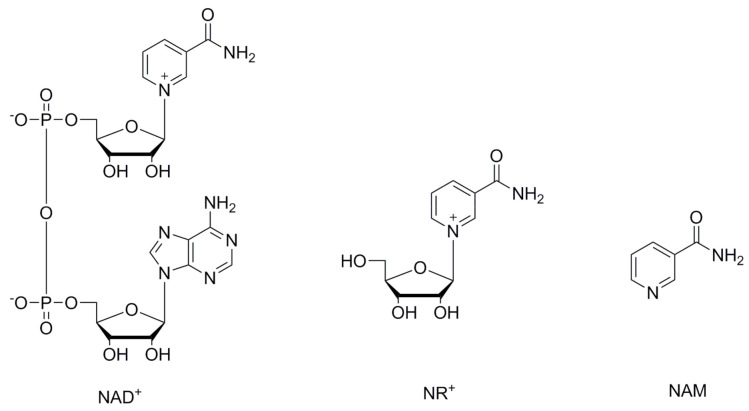
Structures of NAD^+^, NR^+^ and NAM.

Nicotinamide adenine dinucleotide (NAD^+^) (Figure 1) is a redox cofactor for hydride transfer enzymes such as oxidoreductases, with a crucial role in cell metabolism and in the response to oxidative stress [1,2,3,4]. Additionally, NAD^+^ takes part in vital processes not involving hydride transfer such as signaling [1,5], post-translational modification [1,3,6], transcription regulation [1], DNA repair, circadian activity modulation [7,8], and in the regulation of protein–protein interactions. Decreased levels of NAD^+^ are associated with the nuclear and mitochondrial dysfunctions associated with aging [1,2,4,7,9,10] as well as pathophysiological conditions such as ischemia [11,12,13], obesity [2,4,14,15,16], type 2 diabetes [2,17,18,19], metabolic syndrome [2,4,19], non-alcoholic fatty liver disease [2,19], and neurodegenerative disorders [2,4,20,21]. The understanding of the mechanisms and roles of decreased NAD^+^ levels during aging and age-related diseases is of great interest to the scientific community. Numerous studies have been published, many of them showing that supplementing NAD^+^ can increase its bioavailable levels and have beneficial effects [1,2,4,7,9,10]. Increased levels of NAD^+^ can be achieved by supplementation with its precursors, and have great potential in the treatment of pathophysiological conditions involving decreased NAD^+^ levels [4,9,22,23,24]. For this reason, the scientific community has recently been focusing on the roles of NAD^+^ key precursors, with emphasis on nicotinamide riboside (NR^+^) (Figure 1) [11,22,23,24]. NR^+^ is a niacin equivalent and a form of vitamin B3 naturally present in milk [25,26]. The recommended daily intake (RDI) or allowance (RDA) for adults is about 20 mg (equivalent to 0.2 mmol) [27] and it is commercially available as an additive in nutritional supplements and pharmaceutical compositions [23,28,29]. NR^+^ is converted into NAD^+^ by NR^+^ kinase [30], or by the nucleoside phosphorylase and the nicotinamide (NAM) (Figure 1) salvage pathway [9]. When orally supplemented, a preventive and therapeutic effect in numerous diseases has been observed in association with increased NAD^+^ levels [9,11,22,23,24,30,31]. NR^+^ is preferred over other NAD^+^ precursors because its use is not related to serious side effects or flushing, as has been observed with other NAD^+^ precursors [26,32].

**Scheme 1:**
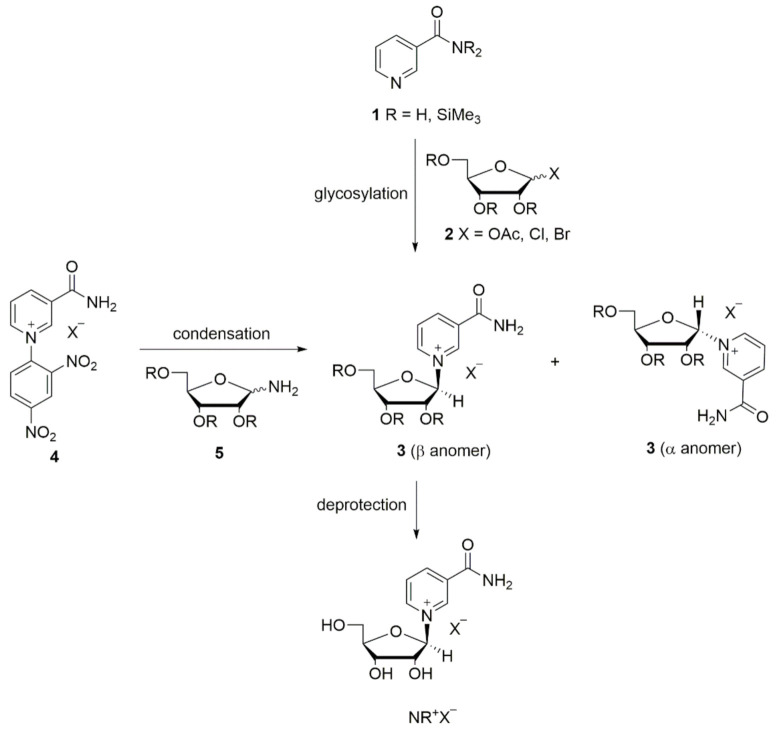
Main approaches for the NR^+^ synthesis. Adapted from Makarov and Migaud [33].

The synthesis and manipulation of NR^+^ salts is challenging due to the relatively labile glycosidic bond compared to other nucleosides [33]. Until now, only the bromide and the chloride of NR^+^ were known as crystalline salts, and only recently have the crystal structures of NR^+^ bromide, chloride, and other non-phosphate ester derivatives been elucidated [34]. Due to its toxicity, the bromide salt is unsuitable as a food supplement. The chloride salt of NR^+^ instead has been generally recognized as safe (GRAS) by the FDA, and numerous studies in humans support this statement. Therefore, the chloride salt of NR^+^ is currently commercially available as a dietary supplement [28,35,36,37,38,39,40,41]. Several synthetic routes towards NR^+^ salts have been reported with two main approaches. The first is the glycosylation of NAM or its derivatives **1** with halosugars or peracylated ribofuranoses **2**, forming the NR^+^ salt via its acylated intermediate **3**. The second approach involves the condensation reaction between *N*-(2,4-dinitrophenyl)-3-carbamoylpyridinium salts **4** and derivatives of d-ribofuranosylamine **5** (Scheme 1) [33]. The synthesis of NR^+^ often achieves poor stereoselectivity, with formation of a mixture of α and β NR^+^ anomers. Since only the β form is biologically active, high stereoselectivity towards the β NR^+^ anomer is highly desirable [33].

**Scheme 2:**
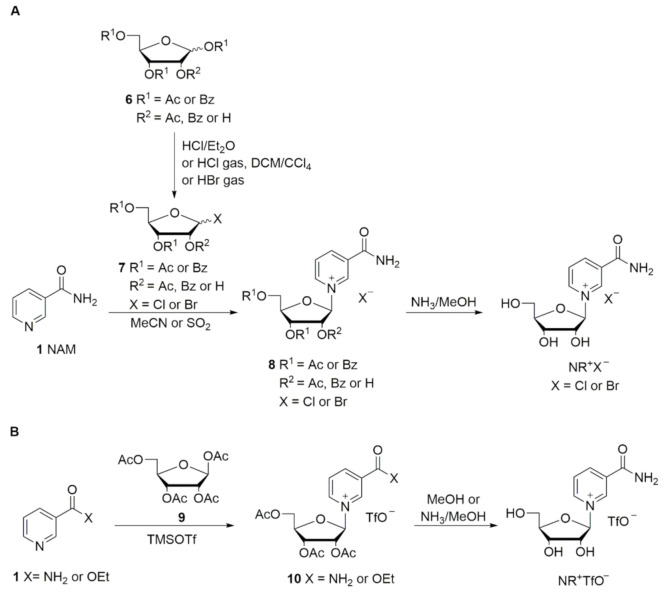
Adapted from Makarov and Migaud [33].

In the first reports describing reactions between NAM and halosugars **7**, Todd and coworkers [42,43] obtained triacetylated or tribenzoylated NR^+^ salts **8** in anhydrous acetonitrile, followed by deprotection in methanolic ammonia at low temperature (Scheme 2A) [33,42,43]. Mikhailopulo et al. reported the synthesis of the acetylated and benzoylated bromide intermediates **8** in liquid SO_2_ (Scheme 2); however this solvent is not suitable for large-scale industrial production [44]. Lee et al. investigated the anomeric ratio obtained in the synthesis of acylated or benzoylated bromosugars **7** via bromination with HBr, which were then reacted with NAM in the glycosylation step (Scheme 2A). A 1.5:1 mixture of β/α anomers was obtained from the acylated sugar, while a 10:1 mixture was obtained with the benzoylated sugar [45].

Although the yields and stereoselectivity of NR^+^ bromide were generally improved compared to the chloride salt [33,45,46,47,48], the bromide is unsuitable for biological applications due to its toxicity. Therefore, an ion exchange step to the chloride is required to obtain a salt with a pharmaceutically acceptable counter ion. The conversion of NR^+^ bromide to NR^+^ chloride via ion exchange methods is not economically attractive for large quantities due to its low exchange capacity. For a complete exchange, either a large exchanger quantity with a correspondingly large amount of solvent is required, or a time-consuming multiple-step process execution is necessary. Moreover, the energy-intensive removal of large quantities of solvent promotes the undesired hydrolysis of NR^+^.

Recently Migaud et al. [47] reported the synthesis of triacetylated NR^+^ chloride in a 6:4 mixture of β/α anomers, with the pure β anomer isolated simply by filtration and washing with acetone. The best deacetylation conditions were reported to be an anhydrous methanolic solution of HCl. Nevertheless, the use of hydrogen chloride, either gaseous or produced in situ, present some issues when scaling-up for industrial production. The use of gaseous hydrogen chloride is expensive in terms of apparatus, while its production in situ requires the use of highly reactive and corrosive reagents which are potential sources of danger.

An alternative synthetic route involves the Hilbert–Johnson method, in which silylated heterocyclic bases are glycosylated with acylated (halo)ribofuranoses in the presence of a Friedel–Crafts catalyst, such as TMSOTf [33]. Tanimori et al. [49] reported the glycosylation of NAM with 1,2,3,5-tetra-*O*-acetyl-β-d-ribofuranose **9** in acetonitrile at room temperature in the presence of 7.3 equivalents of TMSOTf. The formed acetylated NR^+^ triflate salt **10** was deprotected by methanolysis (Scheme 2B). Improved methods have since been reported by Franchetti et al. [50] and Dellinger et al. [51].

Since the triflate is not a pharmaceutically acceptable counter ion, ion exchange is required. This can be achieved by washing with saturated solutions of sodium chloride [52], or by employing ion-exchange resins [53]. Sauve et al. [54,55,56] reported a convenient two-step, one-pot synthetic route consisting of the coupling of ethyl nicotinate and the acetylated sugar **9** in the presence of one equivalent of TMSOTf in dry dichloromethane. Successive deacetylation and hydrolysis of the ethyl ester was performed in one step with methanolic ammonia (Scheme 2B). Felczak [57] described the synthesis of pharmaceutically acceptable salts of NR^+^ starting from silylated NAM and the chlorosugar. However, NR^+^ chloride was isolated after deacetylation as a 3:2 β/α mixture in 38% yield only.

In summary, the production of NR^+^ chloride on the industrial scale is associated with relatively high costs due to the complex and process-intensive synthesis, low yields, poor stereoselectivity, and the use of hazardous or expensive starting materials. The synthesis of the bromide salt is easier, affords higher yields and has better stereoselectivity. However, an additional ion-exchange step is required, which is not ideal for a large-scale synthetic procedure. The discovery of a scalable and efficient synthetic procedure of NR+ pharmaceutically acceptable salts is of great interest from the nutrition perspective and would achieve a very significant market volume comparable to vitamin B12, once a suitable process of manufacturing has been established.

## 2. Materials and Methods

### 2.1. Chemical Synthesis

All reactions were carried out in closed glass vessels under atmospheric pressure without special need for an inert atmosphere. Unless otherwise stated, there was no need for special precautions to exclude moisture. Reagents and solvents were used as purchased without further purification. Proton and carbon NMR spectra for all compounds were acquired on a Bruker Avance III HD 400 MHz spectrometer (Bruker Switzerland AG, Faellanden, Switzerland) at room temperature. Chemical shifts were directly referenced to the residual non-deuterated solvent signal. Ion Chromatography (IC) was performed on a Dionex ICS-2000 System (Thermo Fisher Scientific AG, Reinach, Switzerland). Thin layer chromatography was performed using Merck silica gel 60 F254 plates (Merck KGaA, Darmstadt, Germany) with a solvent system *n*-butanol/water/acetic acid 5:3:2 and visualized with UV light. Melting points (Mp) were measured on a Büchi B-540 melting point apparatus (Büchi Labortechnik AG, Flawil, Switzerland). The synthetic procedures are reported in the Supplementary Information.

### 2.2. Single Crystal Growth Experiments

Single Crystal Growth experiments were attempted with nicotinamide-β-d-riboside d-hydrogen tartrate, nicotinamide-β-d-riboside l-hydrogen tartrate, nicotinamide-β-d-riboside l-hydrogen malate, and nicotinamide-β-d-riboside d-hydrogen malate, all provided in powder form by Biosynth Carbosynth (Staad, Switzerland). Each nicotinamide riboside derivate was left to crystallize using 7 different crystallization trials for a total of 28 crystallization setups. Four different methods were used: vapor diffusion, controlled cooling, under oil crystallization, and combined heating with anti-solvent precipitation.

Vapor Diffusion was trialed for all the nicotinamide riboside derivatives [58]. For each setup, a saturated solution of the compound in 1 mL of methanol (MeOH) was made and filtered. The solution was transferred to a thin vial. A larger scintillation vial was then filled with about 3 mL of the anti-solvent, tetrahydrofuran (THF). The thin vial was then gently placed inside the larger, out vessel, sealed off, and left to crystallize. A second trial series was performed using acetonitrile (MeCN) as the solvent and tetrahydropyran (THP) as the anti-solvent. When the crystals had formed, the vessel was opened, and a single crystal was removed for the single crystal X-ray diffraction analysis. With this method, crystals of nicotinamide-β-d-riboside d-hydrogen tartrate **11a** were obtained by vapor diffusion of THF into MeOH; the crystals are shown in Figure S1A, Supplementary Materials.

Controlled cooling was trialed for all the nicotinamide riboside derivatives. A saturated solution of each compound in MeOH, MeCN, and a 4:1 ratio of MeOH to water were separately produced and filtered. An amount of 200 μL of each of the 12 solutions was transferred to a small, liquid sampling vial and capped off. The trials were set in a Styrofoam box to slow the rate of cooling and placed in the refrigerator. At regular intervals, the crystallization setups were checked. When crystals had formed, the vial was opened, and a single crystal was selected and removed for the single crystal X-ray diffraction analysis. Using this method, crystals of nicotinamide-β-d-riboside d-hydrogen tartrate monohydrate **11b** and l-hydrogen tartrate **12** respectively, were both obtained from the 4:1 MeOH to water solvent mixture; the crystals are shown in Figure S1B,C, Supplementary Materials.

Under oil crystallization was trialed for all the nicotinamide riboside derivates [59]. A saturated solution of each compound in either water or a 4:1 ratio MeOH to water was produced separately and filtered. Eight wells were filled with 100 μL of silicone oil. 5 μL of the saturated solution was then added to each well. Using this method, no crystals were obtained.

Nicotinamide-β-d-riboside l-hydrogen malate **13** and nicotinamide-β-d-riboside d-hydrogen malate **14** were crystallised by thermal crystallization and cooling. Compound **13** was crystallized by dissolving 100 mg of the salt **13** in 76 μL of double distilled water at 37 °C maintained by a water bath, while stirring. The stirring was turned off and a total of 200 μL of ethanol was added in four portions over a period of half an hour. The vial was taken out of the water bath and after 20 min, large colourless crystals had formed. Compound **14** was crystallized by dissolving 50 mg of the salt **14** in 102 μL of double distilled water at 37 °C maintained by a water bath, while stirring. The heating of the water bath and the stirring were turned off and 204 μL of ethanol was added. After two days, large colourless crystals had formed.

### 2.3. X-ray Single Crystal Diffraction

Crystallographic data were collected at 160.0(1) K on a Rigaku-Oxford Diffraction XtaLAB Synergy-S dual source diffractometer (Rigaku Europe SE, Neu-Isenburg, Germany). This is a kappa-axis four-circle goniometer with a Dectris Pilatus3 R 200K HPC (Hybrid Photon Counting) detector (DECTRIS Ltd., Baden-Daettwil, Switzerland) and Cu and Mo PhotonJet microfocus X-ray sources. Suitable crystals were covered with oil (Infineum V8512, formerly known as Paratone N; Infineum International Ltd., Abingdon, United Kingdom), placed on a nylon loop mounted on a CrystalCap Magnetic™ pin (Hampton Research, Aliso Viejo, CA, USA), and immediately transferred to the diffractometer. The program suite CrysAlisPro was used for data collection, numerical and multi-scan absorption correction as well as data reduction [60]. The structures were solved with the dual-space algorithm using SHELXT [61] and refined by the full-matrix least-squares methods on F^2^ with SHELXL-2018 [62] using the Olex2 GUI [63]. The graphical output was produced with the help of the program Mercury [64]. The calculation of the conformation of the ribose rings was done with the help of the program Platon [65] following the description reported by Saenger [66]. CCDC entries 2048059–2048063 contain the supplementary crystallographic data for this paper. These data are provided free of charge by The Cambridge Crystallographic Data Centre via www.ccdc.cam.ac.uk/structures.

### 2.4. Stability Studies of NR^+^ Salts **1**–**4**

The stability of 1 mg/mL aqueous solutions of NR^+^ hydrogen tartrate, hydrogen malate, chloride and bromide was tested by HPLC analysis using a Thermo Fischer Ultimate 3000 instrument (Thermo Fisher Scientific AG, Reinach, Switzerland); Column: Interchim Uptisphere SCX 120 Å, 150 × 4.6 mm, 3 μM; Mobile phase: 100 mM NH_4_HCOO^−^ aqueous Buffer, pH 3.20, MeCN; Method: isocratic 70% MeCN; Detection: UV 254 nm; Flow rate: 1.50 mL/min; Column Oven Temperature: 30 °C; Injection Volume: 10 μL; Run time: 15 min). The samples were run in phosphate buffer at various acidic and neutral pHs (pH 2.5, pH 3.5, pH 4.5, pH 7.0, 0.75% hydrochloric acid), and at different temperatures (5 °C, RT, 40 °C) over time (daily/weekly). The relative peak area of NAM (retention time 1.7 min), formed by degradation of the NR^+^ salt, was used as an index of stability.

## 3. Results

In order to overcome the above-described limitations regarding the synthesis of NR^+^ salts, we investigated alternatives to chloride as the NR^+^ counterion. Our aim was to introduce new pharmaceutically acceptable NR^+^ crystalline salts, which would be better suited to industrial manufacturing, with lower production costs and easier purification to pharmaceutical standards compared to the commercially available NR^+^ chloride. Our initial goal was to screen, identify, and isolate new crystalline forms of NR^+^ salts. Once such compounds were identified, we then searched for a cheap, easily accessible synthetic methodology well suited to large-scale production.

**Scheme 3:**
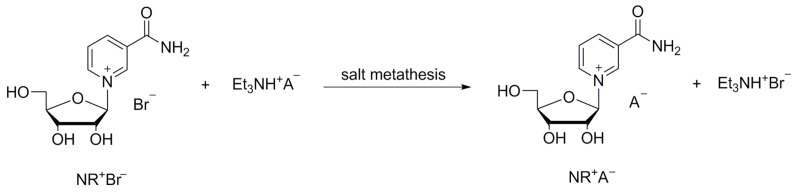
Synthesis of new NR^+^ salt via salt metathesis from NR^+^ bromide and triethylammonium salts.

Initially, we screened different NR^+^ salts searching for new stable and crystalline forms, without using time-consuming and unsuitable hydrolysis-prone ion exchange procedures. These NR^+^ salts were initially prepared by salt metathesis from NR^+^ bromide and triethylammonium salts of various acids (Scheme 3). The synthesis of neutral salts and acidic salts was attempted.

### 3.1. Preparation of NR^+^ Neutral Salts

When the triethylammonium salts, which are highly soluble in methanol, were added to a suspension of NR^+^ bromide in methanol, a clear solution was obtained upon gentle heating. By decreasing the temperature of the solution, usually no solid crystallized, presumably because the dissolved NR^+^ bromide increased the polarity of the supersaturated solution. Crystallization of the undesired bromide salt was observed only with the formiate, salicylate, and maleate salts. Additionally, we observed that in the presence of longer chain alcohols, NR^+^ bromide crystallized with lactate, malonate, methane sulfonate, and sorbate salts. Thus, these salts are either more soluble in methanol compared to the bromide, or not able to form crystals at all. No solid or waxy solid was obtained with benzenesulfonate, fumarate, or succinate.

We expected NR^+^ to be most stable in solutions of the neutral salts of strong mineral acids such as sulfuric acid, or strong organic acids such as benzenesulfonic and methanesulfonic acid. However, in the presence of the corresponding triethylammonium salts we observed no formation of crystals. Carbonic acid salts were not stable towards hydrolysis due to their weakly basic properties and therefore no crystallization was expected.

Furthermore, only a small selection of the desired new salts formed by metathesis precipitated as stable solids by adding the obtained solution in methanol to longer chain alcohols, such as *n*-propanol, *n*-butanol, and isopropanol. These are the NR^+^ salts of l-ascorbic acid, citric acid, d-glucuronic acid as well as of l-malic and l-tartaric acid. These amorphous solids were purified by trituration from alcohols.

The described results are reported in Table 1.

### 3.2. Preparation of NR^+^ Acidic Salts

The synthesis of NR^+^ salts of acidic dicarboxylates was investigated following a similar procedure used for the neutral NR^+^ salts. In the presence of acidic salts of di- or tri-carboxylic acids, NR^+^ proved to be more stable, but in most cases the NR^+^ bromide crystallized.

NR^+^ bromide crystallized with the acidic carboxylates of citrate, fumarate, maleate, malonate, mercaptosuccinate, α-ketoglutarate, oxaloacetate, oxalate, succinate, and tartronate. NR^+^ dihydrogen citrate and meso-hydrogen tartrate formed as unstable solids when the metathesis solution was dropped into isopropanol and ethanol, respectively. To our surprise, NR^+^ crystalline salts formed with d, l, dl hydrogen malate and d, l, dl hydrogen tartrate. The described results are reported in Table 2.

**Figure 2:**
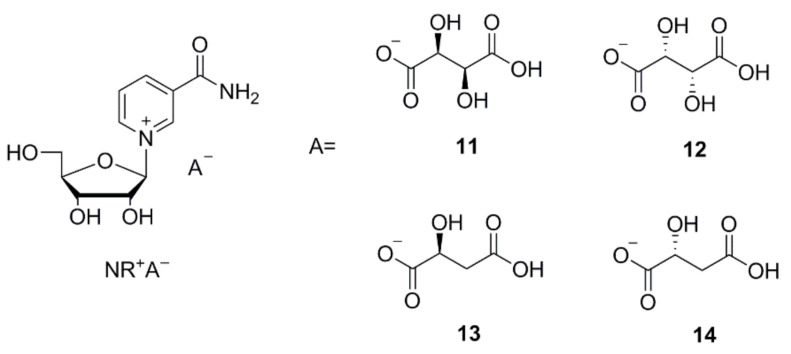
Structural formulas of the crystalline NR^+^ derivatives: nicotinamide-β-d-riboside d-hydrogen tartrate (**11**), nicotinamide-β-d-riboside l-hydrogen tartrate (**12**), nicotinamide-β-d-riboside l-hydrogen malate (**13**), nicotinamide-β-d-riboside d-hydrogen malate (**14**).

The new crystalline salts, which are the acidic NR^+^ salts of tartaric and malic acid (NR d-hydrogen tartrate **11**, NR l-hydrogen tartrate **12**, NR l-hydrogen tartrate **13,** and NR d-hydrogen tartrate **14**, shown in Figure 2) were isolated by using the above-described method not requiring elaborate crystallization experiments. These were formed immediately from salt metathesis with NR^+^ bromide and were isolated with yields and purity ranging from good to excellent. Furthermore, we determined that the residual bromide content was quite low (Table 3). Other bases such as tributylamine or tetrabutylammonium hydroxide can be used instead of Et_3_N, as reported in examples 10b, 10c, and 12b in the SI.

### 3.3. Single Crystal Growth of Newly Synthesized NR^+^ Salts

Several re-crystallization attempts, which differed by method (vapor diffusion, controlled cooling, and under oil crystallization) and solvent type were set up for **11** through **14**.

**Figure 3:**
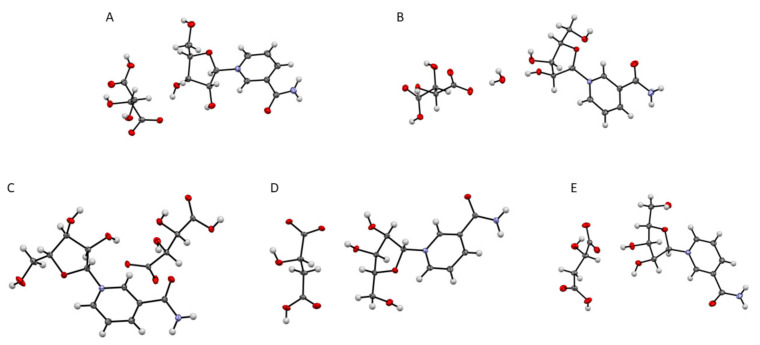
Displacement ellipsoid representations at 50% probability of: **11a** (**A**) obtained by vapor diffusion; **11b** (**B**) and **12** (**C**) obtained by controlled cooling; **13** (**D**) and **14** (**E**) obtained by thermal crystallization and addition of an anti-solvent.

After one week, single crystals of an anhydrate and hydrated derivative of **11** (**11a**, **11b**), and **12** were obtained. Rather rapid cooling delivered single crystals of **13** and **14** within half an hour, as well as two double salt derivatives: dinicotinamide-β-d-riboside l-hydrogen tartrate-d-hydrogen tartrate and dinicotinamide-β-d-riboside l-hydrogen malate-d-hydrogen malate which were crystallized via thermal crystallization. The crystalline nature was verified by powder X-ray diffractograms and single crystal structure investigations as shown in Figure 3 (additional data in Supporting Information). Interestingly, NR^+^ *meso*-hydrogen tartrate was not crystalline.

Single crystals of **11a** were obtained by vapor diffusion at room temperature from a saturated solution in methanol (MeOH), against a reservoir of tetrahydrofuran (THF). The single crystal X-ray diffraction data is summarized in Table S4, Supplementary Materials. **11a** crystallized in the monoclinic space group, P2_1_. The asymmetric unit consists of one molecule of **11**, with two formula units per unit cell. The ribose sugar is in the C2’ endo, twisted conformation. The absolute configuration of the enantiomerically pure molecule was confirmed by the Flack parameter (0.03(7)) and by the expected stereochemistry.

Monohydrated single crystals of **11b** were obtained by way of controlled cooling of a saturated solution in MeOH with 20% water at room temperature to 4 °C. Via this method, **11b** crystallized in the chiral, orthorhombic space group, P2_1_2_1_2_1_. The asymmetric unit consist of **11** and a water molecule, which most likely originated from the non-dried methanol or from the humidity in the air. There are four formula units in one cell. The ribose sugar of **11b** (monohydrated) is in a C3′ endo envelope conformation. The absolute configuration of the enantiomerically pure molecule was confirmed by the Flack parameter (0.02(5)) and its comparison to the expected stereochemistry. **11** was also characterized by powder X-ray diffraction. The measured powder pattern is shown in Figure S2A. The powder pattern looks similar to the calculated powder pattern, simulated from single crystal data of the monohydrate (Figure S2C, Supplementary Materials), and less similar to the simulated pattern of the anhydrous crystal (Figure S2B, Supplementary Materials). This comparison indicates that the bulk compound exists as a monohydrate.

Single crystals of **12** were obtained by way of controlled cooling of a saturated solution of **12** dissolved in a 4:1 ratio of MeOH and water. The crystallographic data is summarized in Table S4, Supplementary Materials. **12** crystallized in the monoclinic space group P2_1_. There are two formula units per unit cell and one molecule in the asymmetric unit. The ribose sugar is in a C2′ endo envelope conformation. **12** was also characterized by powder X-ray diffraction. The measured powder pattern is shown in Figure S3A, Supplementary Materials. The powder pattern looks similar to the calculated powder pattern, simulated from single crystal data (Figure S3B, Supplementary Materials).

**13** crystallized in the monoclinic space group, P2_1_. The asymmetric unit consists of one molecule of **13**, where the carboxylic acid of the first carbon is deprotonated. The unit cell holds two asymmetric units of molecule **13**. The ribose sugar is in a C3′ endo envelope conformation. The absolute configuration of the enantiomerically pure molecule was confirmed by the Flack Parameter (0.03(14)) and by the expected stereochemistry. **13** was characterized by powder X-ray diffraction. The measured powder pattern is shown in Figure S4A, Supplementary Materials. The powder pattern is similar to the calculated powder pattern, simulated from the single crystal data (Figure S4B, Supplementary Materials).

**14** crystallized in the chiral, orthorhombic, space group P2_1_2_1_2_1_. The asymmetric unit contains only **14**, where the carboxylic acid attached to the first carbon is deprotonated, and there are four formula units per unit cell. The ribose sugar has a C3′ endo envelope conformation. The absolute configuration of the enantiomerically pure molecule was confirmed by the Flack parameter (0.01(7)) and the expected stereochemistry. The powder pattern of **14** was simulated using the single crystal data. The result is shown in Figure S5, Supplementary Materials.

**Figure 4:**
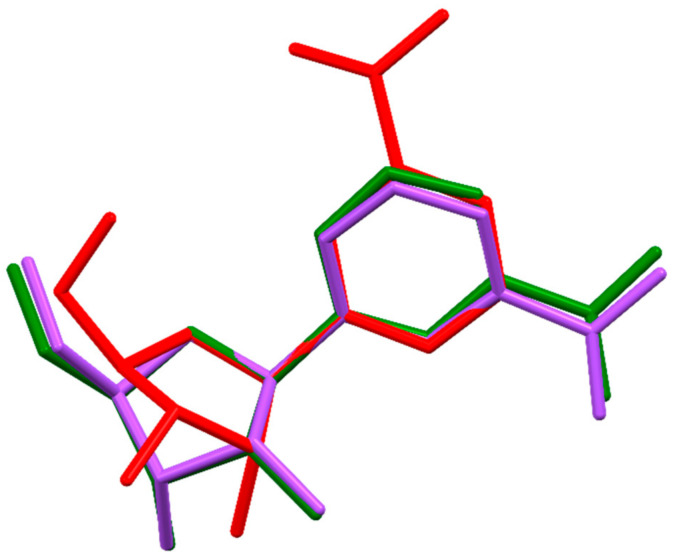
Overlay of the nicotinamide-β-d-riboside cations of **11a**, **11b,** and **12**. The structures were overlayed on atoms O4, C1, and N1 with the help of the program Mercury. [64]

By comparison of **11**–**14**, it is evident that the anion type, and the stereochemistry of the anion is decisive for the overall structure. The space groups, the sugar pucker [66] conformations of **11**–**14** and a bromide and chloride salt from a previous work [34] as well as the χ(O4-C1-N1-C6) torsion angles) are given in Table 4. The two anhydrous hydrogen tartrate diastereomers crystallized both into the space group P2_1_, but the sugar of the d-hydrogen tartrate isomer has a twisted conformation, whereas l-hydrogen tartrate is in the C2´-endo envelope conformation. Alternatively, the hydrogen malate diastereomers did not crystallize in the same space groups. The l-isomer crystallized into the space group P2_1_ like l-hydrogen tartrate, but the d-hydrogen malate salt crystallized in the space group P2_1_2_1_2_1_. Both malate isomers have riboside sugars in the 3´endo conformation. The l-hydrogen malate salt resembles the structures of the bromide and chloride anions of the nicotinamide riboside, as they crystallized into the same space groups with a 3´envelope conformation of sugars. The overlay of three representative structures (**11a**, **11b,** and **12**) are shown in Figure 4. The overlays were calculated by minimizing the distances between the atoms O4, C1, and N1 respectively. Apart from the 2’-endo versus 3’-endo differences, structure **11b** stood out, since it is the only structure with a positive χ torsion angle of 10.9(2)° corresponding to a *syn* nucleoside confirmation. All the other structures have a negative χ angle (between −136.1(1)° and −164.1(2)°) corresponding to an *anti* nucleoside confirmation.

Additionally, the hydrogen bonding found differs in each structure. An attempt was made to simulate the PXRD of the l-hydrogen malate **13** by removal of the hydroxyl group from C14 of the d-hydrogen tartrate **11**, but this simulated pattern was very different from the PXRD pattern of **13**. This might suggest that the presence of one less hydrogen bond donor/acceptor on the anion is enough to change the orientation of the salt and the space group.

In summary, **11a**, **12**, and **13** all crystallized into the same space group, each with a different conformation of the riboside. **11b**, **14**, and the previously reported bromide and chloride salts [34] all crystallized into the same space group with the same sugar pucker conformation. However, the hydrogen bonding differs in each structure. Removal of an alcohol oxygen atom of d-hydrogen tartrate to yield l-hydrogen malate or of l-hydrogen tartrate to yield d-hydrogen malate respectively could in theory yield isostructural structures of the corresponding salt pairs, which is not the case. This suggests that the presence of one less hydrogen bond donor/acceptor on the anion is enough to change the orientation of the salt and the space group. In the absence of DFT studies, a quantitative explanation is not possible. Additionally, we observed that the double salts did not co-crystallize, rather they crystallized as the l- and d-enantiomers, separately [data not shown].

### 3.4. Properties of the New Crystalline NR^+^ Salts

The solubility of the new NR^+^ salts together with chloride and bromide NR^+^ salts was tested. The solubility in water is comparable, however the new NR^+^ salts were far less soluble in methanol (Table S3, Supplementary Materials). The low solubility in methanol is convenient since it allows for the synthesis of the new salts by salt metathesis from the bromide.

The stability of NR^+^ hydrogen tartrate, hydrogen malate, chloride and bromide was tested in phosphate buffer at various acidic and neutral pHs (pH 2.5, pH 3.5, pH 4.5, pH 7.0, 0.75% hydrochloric acid), and at different temperatures (5 °C, RT, 40 °C) (data shown in Supplementary Materials).

The rate of degradation is dependent on temperature. At a given temperature, degradation to NAM occurs at the same rate regardless of NR^+^ salt and pH. Comparing the different salts (different anions), no difference in stability could be found in solution. A slightly higher degradation rate under neutral (pH 7.0) conditions was found for the first few days, especially in the cases of NR^+^ chloride and bromide. NR^+^ hydrogen tartrate and hydrogen malate seem to be more stable at neutral pH, probably because they are slightly acidic and can compensate for the negative effects of the surrounding solvent, being self-buffering.

After 3 weeks at 40 °C in solution, NR^+^ is almost completely degraded to NAM. A slight flattening of the curve can be observed at higher NAM values. At RT, the degradation appears to be much slower: after 3 weeks only about 20% NAM is formed, compared to about 90% at 40 °C. At 5 °C after 3 weeks, just 1% NAM has formed, while when stored at pH 7.0 and a halogen as counter ion approximately 1.4% NAM formed. It can be concluded that the temperature has a decisive influence on the stability of the product in solution, while the pH has little impact.

**Figure 5:**
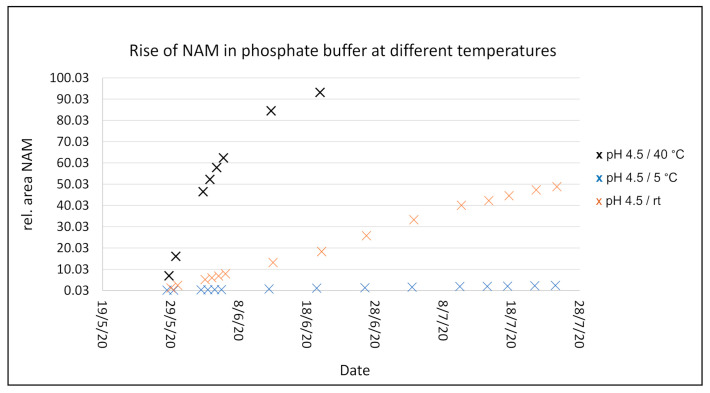
Degradation rate of NR^+^ l-hydrogen malate to NAM as peak area measurement over time at pH 4.5 at different temperatures.

The graph from Figure 5 shows the degradation rate of NR l-hydrogen malate into NAM at pH 4.5. A similar pH is obtained after dissolving the salt in water that, due to its slightly acidic character, brings the pH to this value. As explained, the pH has an insignificant effect on the degradation rate, while the temperature strongly influences it. The data was collected at 40 °C, 5 °C, and room temperature over several weeks, showing a much faster degradation for the higher temperature, while at low temperature it remains very stable. The rest of the salts behave similarly (individual data shown more in detail in the Supporting Information).

### 3.5. Improved Synthetic Approach via the Triflate NR^+^

**Scheme 4:**
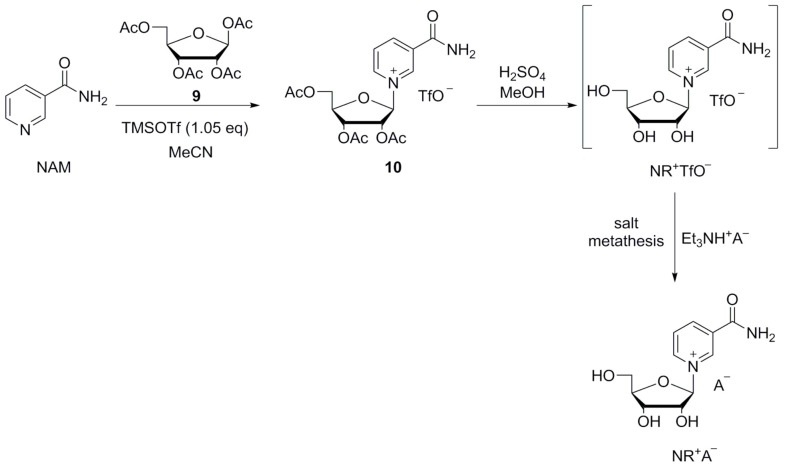
Improved synthetic route via NR triflate.

Thanks to a thorough screening, we were able to identify new crystalline salts of NR^+^, which were initially synthesized via salt metathesis from NR^+^ bromide. While this procedure is high yielding, manufacturing large amounts of NR^+^ bromide would be too expensive for industrial production, and so an alternative synthetic route was explored. This involves deacetylation of NR^+^-triacetate triflate, followed by pH adjustment and salt metathesis with triethylammonium salts (Scheme 4).

NR^+^-triacetate triflate is formed directly from ribose tetraacetate and NAM activated in situ with TMSOTf, with an almost quantitative turnover. Since no excess reagent is required, no by-products must be separated, and no special vacuum pumps are needed. The reaction is easy to carry out without special equipment and proceeds gently. The stable NR^+^-triacetate triflate can be processed and deacetylated immediately, and the final product crystallizes directly from the reaction solution upon salt metathesis. All three stages can be carried out in one go, making it a convenient one-pot process.

NR^+^-triacetate iodide was also synthesized, as reported in examples 17 and 18 in the SI. However, the reaction required a higher temperature to proceed (40–45 °C) and the conversion did not go to completion. For this reason, the synthesis of the triflate derivative is preferred.

By comparison, the 1-chloro-2,3,5-triacetyl-d-ribofuranose must be prepared separately using HCl gas or acetyl chloride. This is difficult to scale up because the chlorosugar is unstable under the reaction conditions. For full conversion, excess HCl is necessary. Removal of excess HCl requires special, corrosion-resistant vacuum pumps and increased temperatures that are unfavorable for the glycosylation.

According to Tanimori et al. [49] glycosylation of NAM takes place on activation of ribose tetraacetate **10** by an excess of TMSOTf (Scheme 4). We found, however, just over one equivalent is sufficient for almost complete conversion. The avoidance of such a large excess of the expensive, hazardous, and air-sensitive TMSOTf is a great economical advantage compared to previously described methodologies. The triacetate was not isolated by the authors, but immediately deacetylated in situ after the addition of methanol. The deprotected NR^+^ triflate was described by the authors as a crystalline substance, but no evidence supporting this statement was reported. However, the triflate obtained from our experiments proved to be non-crystalline. Surprisingly, ammonia is used in most procedures to remove the acyl protecting groups of the NR^+^ salt [42,43,45,46,50,54,55], although an alkaline environment leads to considerable decomposition. We also proved experimentally that the resinous NR^+^-triacetate triflate **10** may be deacetylated with ammonia in methanol. However, we realized that deacetylation with mineral acids is a cleaner procedure leading to the formation of less impurities. We found that deacetylation is also possible by employing triethylamine, obtaining higher yields (Scheme 4).

Additionally, we attempted deacetylation with HBr and we observed that the bromide crystallized, but its yield was unexpectedly low. When we used HCl the yield of the chloride resulted in being even lower. This shows that both halogenides are not ideal to isolate the NR^+^ out of the metathesis mixture. The difference in yield clearly highlights the benefit of the new NR^+^ salts described in this work for economic synthesis of NR^+^ salts. We would therefore recommend using sulfuric acid for deacetylation, because it avoids the disturbing crystallization of undesired salts such as NR^+^ bromide. The overall yields (from the three steps of glycosylation, deacetylation, and salt metathesis) obtained with the various deacetylation methods are reported in Table 5.

The improved synthetic procedure described above is extremely convenient because once the NR^+^ cation is formed, it can be converted into a desired NR^+^ salt simply by double conversion, provided that the salt is either crystalline or at least precipitable as an amorphous solid. To promote precipitation or crystallization of the NR^+^ salt, a pH adjustment can be performed. With this newly described method, the desired NR^+^ hydrogen tartrate or hydrogen malate salts precipitated from the reaction solution as the least soluble and the only crystalline phase. Due to the low solubility and high crystallization tendency of the new salts in methanol, the products obtained are already quite pure and only require one further recrystallization to be suitable for food additives.

## 4. Conclusions

In conclusion, after a thorough screening, we discovered new pharmaceutically suitable NR^+^ salts, specifically high-crystalline NR^+^ hydrogen malate and NR^+^ hydrogen tartrate salts with numerous favorable properties. These salts were initially synthesized via salt metathesis from NR^+^ bromide. A fast re-crystallization step allowed us to obtain single crystals and elucidate their crystal structure. Once pharmaceutically suitable forms of NR^+^ salts were identified, the synthetic procedure was greatly improved to a high yielding one-pot procedure, not requiring specific and expensive apparatus. The improved yields and the ease of production are suitable for large-scale industrial manufacturing compatible with distribution in the food supplement global market.

The newly reported compounds are salts of well-known anions, which are metabolites produced in vivo and also present in the market as food supplements. The new NR^+^ salts can therefore be safely administered orally as food supplements. In fact, NR^+^ is fully solvated in water and its reactivity and physiology does not depend on its counter ion, as long as this is benign.

## 5. Patents

Some of the experimental results and procedures presented in this manuscript are collected in the patent application number WO 2021/013795 A2.

## Figures and Tables

**Table 1:** Salt metathesis results with NR^+^ neutral salts.

A in Et_3_N·A	NR^+^Br^−^ (g)	H_2_O (mL)	MeOH (mL)	S/D*	Et_3_N·A (mL)	Solvent Added (mL)	NR·A (g)	NR^+^Br^−^ (g)	Yield %
l-Ascorbate	5.0	3	10	D	16	375 EtOH	3.45	-	54
Benzenesulfonate	0.56	0.4	1	D	1	-	-	-	0
Citrate	9.0	-	18 (DMSO)	D	73	1125 ^i^PrOH	6.32	-	75
Formiate	10.0	-	25	S	12.5	-	-	6.21	0
Fumarate	0.50	0.3	1	S	0.5	-	-	-	0
d-Glucuronate	5.0	3	10	D	11.3	455 *n*-BuOH	6.64	-	99
Lactate	5.80	-	20	S	10	400 ^i^PrOH	-	4.27	0
l-Malate	5.0	-	15	S	10	280 ^i^PrOH	4.51	-	100
Maleate	0.94	-	1	S	1	-	-	0.24	0
Malonate	7.10	4.3	14	D	7.3	350 ^i^PrOH	-	5.75	0
Methane sulfonate	0.62	0.4	1	D	1	24 *n*-BuOH	-	0.23	0
Salicylate	2.75	-	9	S	5	-	-	1.73	0
Sorbate	2.90	1.5	5	D	5	100 Dioxane	-	1.93	0
Succinate	0.48	0.3	1	S	0.5	-	-	-	0
l-Tartrate	9.65	5.8	20	D	10	500 EtOH	8.06	-	85

* S = suspended, D = dissolved.

**Table 2:** Salt metathesis results with NR^+^ acidic salts.

HA in Et_3_N·HA	NR^+^Br^−^ (g)	H_2_O (mL)	MeOH (mL)	S/D*	Et_3_N·HA (mL)	NR·HA (g)	NR^+^Br^−^ (g)	Yield %
Hydrogen citrate	0.92	-	2	S	1	-	**	0
Dihydrogen citrate	0.41	-	1	S	1	0.23		42
Hydrogen fumarate	0.58	-	1.5	S	1	-	**	0
Hydrogen maleate	0.58	-	1.5	S	1	-	0.13	0
d-Hydrogen malate	5.80	-	10	S	10	4.01		60
l-Hydrogen malate	5.80	-	10	S	10	4.15		62
dl-Hydrogen malate	5.80	-	10	S	10	4.52		67
Hydrogen malonate	0.60	-	1	S	1	-	0.07	0
H-mercaptosuccinate	0.58	-	1.5	S	1	-	**	0
Hydrogen ketoglutarate	0.58	-	1.5	S	1	-	**	0
Hydrogen oxalacetate	0.60	-	1	S	1	-	**	0
Hydrogen oxalate	0.62	-	1	S	1	-	0.15	0
Hydrogen succinate	0.58	-	1	S	1	-	**	0
d-Hydrogen tartrate	5.80	-	10	S	10	4.90		70
l-Hydrogen tartrate	5.80	3.5	10	D	10	6.62		95
dl-Hydrogen tartrate	5.80	3.5	10	D	10	6.31		90
meso-Hydrogen tartrate	0.57	-	1	S	1	0.44		62
Hydrogen tartronate	0.60	-	1	S	1	-	**	0

* S = suspended, D = dissolved. ** The product was not dried and therefore the yield not determined.

**Table 3:** Purity and residual bromide of crystalline NR^+^ hydrogen malates and tartrates.

NR^+^ Salt	Purity (NMR)	Residual Br (IC)
d-Hydrogen tartrate **11**	>97%	0.3%
l-Hydrogen tartrate **12**	>97%	0.2%
dl-Hydrogen tartrate **11** + **12**	>97%	0.1%
l-Hydrogen malate **13**	>97%	0.1%
d-Hydrogen malate **14**	>97%	0.9%
dl-Hydrogen malate **13** + **14**	>96%	2.3%

**Table 4:** Sugar conformation of different NR^+^ derivatives.

Compound	Space Group	Conformation	χ(O4-C1-N1-C6) [°]	Ref.
Nicotinamide-β-d-riboside d-Hydrogen tartrate anhydrate (11a)	P2_1_	2’-endo, Twisted (inbetween C1’-exo and C2’-endo)	−157.0(2)	This work
Nicotinamide-β-d-riboside d-Hydrogen tartrate hydrate (11b)	P2_1_2_1_2_1_	3’-endo, Envelope	10.9(2)	This work
Nicotinamide-β d-riboside l-Hydrogen tartrate (12)	P2_1_	2’-endo, Envelope	−158.10(9)	This work
Nicotinamide-β-d-riboside l-Hydrogen malate (13)	P2_1_	3’-endo, Envelope	−154.35(7)	This work
Nicotinamide-β-d-riboside d-Hydrogen malate (14)	P2_1_2_1_2_1_	3’-endo, Envelope	−164.1(2)	This work
Nicotinamide-β-d-riboside bromide	P2_1_2_1_2_1_	3’-endo, Envelope	−137.1(2)	[34]
Nicotinamide-β-d-riboside chloride	P2_1_2_1_2_1_	3’-endo, Envelope	−136.1(1)	[34]

**Table 5:** Yields of the newly obtained NR salts with various methodologies.

Example (SI)	Deacetylation	Neutralization	Salt for SM	Product	Yield %
15a	H_2_SO_4_	Et_3_N	Et_3_N l-H-Tartrate	NR^+^ l-H-Tartrate	54
15b	HBr	-	-	NR^+^ Bromide	25
15b	HBr	Et_3_N	Et_3_N l-H-Tartrate	NR^+^ l-H-Tartrate	53
15c	HCl	-	-	NR^+^ Chloride	2
15c	HCl	Et_3_N	Et_3_N l-H-Tartrate	NR^+^ l-H-Tartrate	60
15d	Et_3_N	-	Et_3_N l-H-Tartrate	NR^+^ l-H-Tartrate	63
16a	H_2_SO_4_	Et_3_N	Et_3_N l-H-Malate	NR^+^ l-H-Malate	37
16b	HBr	Bu_3_N	Et_3_N l-H-Malate	NR^+^ l-H-Malate	33
16c	HCl	Et_3_N	Et_3_N l-H-Malate	NR^+^ l-H-Malate	43
16d	Et_3_N	-	Et_3_N l-H-Malate	NR^+^ l-H-Malate	43

## Supplementary Materials

Section S1: Preparation of neutral salts. Section S2: Preparation of acidic salts. Section S3: Solubility of NR^+^ crystalline salts. Section S4: Single crystal growth and structural determination of nicotinamide riboside derivatives by X-ray diffraction. Section S5: Stability studies of NR+ salts. Figure S1: Photos of the grown crystals of **11a** (anhydrate of 11) (**A**), **11b** (monohydrate of **11**) (**B**), **12** (**C**), **13** (**D**) and **14** (**E**); Figure S2: X-ray powder pattern of **11** (**A**). X-ray powder pattern of **11a** (anhydrous) simulated from single crystal data (**B**). X-ray powder pattern of **11b** (monohydrate) simulated from single crystal data (**C**). Figure S3: X-ray powder pattern of **12** (**A**). X-ray powder pattern of **12** simulated from single crystal data (**B**); Figure S4: X-ray powder pattern of **13** (**A**). X-ray powder pattern of **13** simulated from single crystal data (**B**); Figure S5: X-ray powder pattern of **14** (**A**). X-ray powder pattern of **14** simulated from single crystal data (**B**); Figure S6: NAM peak area measurement over time at RT at various pH for NR^+^-l-hydrogen malate (**A**), NR^+^-dl-hydrogen tartrate (**B**), NR^+^ chloride (**C**), NR^+^ bromide (**D**); Comparison of the different NR^+^ Salts at RT in HCl (**E**); Figure S7: NAM peak area measurement over time at 5 °C at various pH for NR^+^-l-hydrogen malate (**A**), NR^+^-dl-hydrogen tartrate (**B**), NR^+^-chloride (**D**), NR^+^-bromide (**E**); Comparison of the different NR^+^ Salts at 5 °C in HCl (**F**); Figure S8: NAM peak area measurement over time at 40 °C at various pH for NR^+^-l-hydrogen malate (**A**), NR^+^-dl-hydrogen tartrate (**B**), NR^+^-chloride (**D**), NR^+^-bromide (**E**); Comparison of the different NR^+^ Salts at 40 °C in HCl (**F**); Table S1: Carboxylic acids used to prepare the Et3N·A solutions; Table S2: Dicarboxylates used to prepare the Et3N·HA solutions; Table S3: Solubilities (in mL solvent per g of solute) of the stereoisomeric crystalline NR^+^ salts and the halogenides in methanol and in aqueous solutions; Table S4: Summary of Crystal Data and Structure Refinement of the Salts **11a**, **11b**, **12**, **13**, and **124**.

## Sample Availability

Samples of the compounds are not available from the authors.
