# Supplementary material for: New Crystalline Salts of Nicotinamide Riboside as Food Additives
Source: Molecules. 2021 May 6;26(9):2729. doi: 10.3390/molecules26092729 (PMC8125264; doi:10.3390/molecules26092729)
Supplement: Supplementary file 1 [file molecules-26-02729-s001.zip › molecules-1173671-supplementary/Molecules-1173671 - SI.pdf]

Günter Schabert [1], Robert Haase [1], Jaclyn Parris [2], Laura Pala [3], Adrian Hery-Barranco [3], Bernhard Spingler [2] and Urs Spitz [1,3]*

[1] Biosynth Carbosynth, Rietlistrasse 4, 9422 Staad, Switzerland
[2] University of Zurich, Department of Chemistry, Winterthurerstrasse 190, 8057 Zurich, Switzerland
[3] Biosynth Carbosynth, Axis House, High Street, Compton, Berkshire, RG20 6NL, United Kingdom
* Correspondence: urs.spitz@biosynth-carbosynth.com.

New crystalline salts of nicotinamide riboside as food additives

---

Supporting Information

- **Section S1: Preparation of neutral salts**
- **Section S2: Preparation of acidic salts**
- **Section S3: Solubility of NR^+ crystalline salts**
- **Section S4: Single Crystal Growth and Structural Determination of Nicotinamide riboside derivatives by X-Ray Diffraction**
- **Section S5: Stability studies of NR^+ salts**

Section S1: Preparation of neutral salts

Preparation of solutions of Et_3N·(di)-Carboxylate (Et_3N·A), general procedure

The carboxylic acid was suspended (S) or dissolved (D) in methanol (MeOH) while stirring at RT. The solution was cooled in an ice bath and a multi-equivalent amount of triethylamine (Et_3N) sufficient for complete neutralisation was added, after which a slight temperature increase was observed. The pH of the colourless to yellowish solutions was about 8. The exceptions are the glucuronate solution with pH around 7, the fumarate and succinate solutions with pH around 6. With the general method described above, x mL of a y molar Et_3N·carboxylate solution (Et_3N·A) were obtained and used directly in the next step without further purification. The data for the carboxylic acid tested are reported in Table S1.

Table S1: Carboxylic acids used to prepare the Et_3N·A solutions.

Carboxylic acid	Acid Amount	MeOH (mL)	S / D	Et_3N (mL)	x (mL)	y (M)
L-Ascorbic	4.50 g	22.5	S	3.64	27.5	0.93
Benzenesulfonic	4.38 g	10.0	D	3.64	15.5	1.68
Citric	5.52 g*	55.0**	D	12.00	73.00	0.36
Formic	1.40 mL	10.0	D	5.00	15.00	2.40
Fumaric	3.02 g	10.0	S	7.28	17.50	1.49
D-Glucuronic	5.10 g	15.0	S	3.60	19.50	1.32
L-Lactic	2.34 g	10.0	D	3.64	15.00	1.73
L-Malic	3.50 g	10.0	D	7.30	18.00	1.45
Maleic	3.02 g	10.0	D	7.27	18.50	1.40
Malonic	2.70 g	10.0	D	7.27	18.00	1.45
Methanesulfonic	1.70 mL	10.0	S	3.64	14.00	1.86
Salicylic	3.60 g	10.0	D	3.64	16.00	1.63
Sorbic	2.94 g	10.0	S	3.64	15.50	1.68
Succinic	3.07 g	10.0	S	7.28	18.00	1.44
L-Tartaric	3.90 g	10.0	D	7.27	18.00	1.44

* Citric acid monohydrate

** DMSO (instead of MeOH)

Salt metathesis reactions

Example 1: Nicotinamide-β-D-ribofuranoside L-ascorbate

4.50 g (26 mmol) of L-ascorbic acid were suspended in 22.5 mL of methanol while stirring. The colorless suspension was cooled in an ice bath and 3.64 mL (26 mmol, 1 eq) of triethylamine were added. 27.5 mL of a 0.93 M solution of Et_3N·L-ascorbate were prepared. 5.00 g (14.9 mmol, 1 eq) of nicotinamide-β-D-ribofuranoside bromide were dissolved with stirring in 3.0 mL of water at RT. 10 mL of methanol and 16 mL (14.9 mmol, 1 eq) of the above prepared solution of triethylammonium L-ascorbate were added. The yellow-orange solution was dropped slowly to 375 mL of ethanol, wherein a yellow flocculent suspension was produced. The suspension was stirred for a few h at RT.

The product was filtered, washed with ethanol, and dried under vacuum at 35 °C. Nicotinamide-β-D-ribofuranoside L-ascorbate was isolated as a yellow powder (3.45 g, 54%).
3.11 g (7.22 mmol) of crude nicotinamide-β-D-ribofuranoside L-ascorbate were dissolved in 1.9 mL of water. The orange clear solution was diluted with 16 mL of methanol. The solution was dropped slowly to 238 mL of ethanol, wherein an orange suspension was produced. Nicotinamide-β-D-ribofuranoside L-ascorbate was isolated by filtration and dried at 35 °C. Nicotinamide-β-D-ribofuranoside L-ascorbate was isolated as a yellowish powder (1.13 g, 36%). IC: Residual bromide 0.38%.

^{1}H-NMR (400 MHz, D_2O): 3.82 (br dd, 1H, *J*= 12.9 Hz, 3.2 Hz, H5′A), 3.97 (br d, 1H, H5′B), 4.28 (t, 1H, *J*= 4.3 Hz, H3′), 4.34-4.47 (m, 2H, H4′, H2′), 6.17 (d, 1H, *J*= 4.2 Hz, H1′), 8.20 (t, 1H, *J*= 7.2 Hz, H5), 8.89 (br d, 1H, *J*= 7.8 Hz, H4), 9.19 (br d, 1H, *J*= 6.0 Hz, H6), 9.52 (s, 1H, H2); Ascorbate: 3.58-3.70 (m, 2H), 3.92-3.97 (m, 1H), 4.40-4.46 (m, 1H). Impurities: 16 mol% nicotinamide: 7.49 (t, 1H), 8.14 (d, *J*= 8.2 Hz, 1H), 8.60 (d, 1H), 8.82 (s, 1H); no Et_3N salt. Solvents: 1.3 mol% methanol: 3.26 (s, 3H); 46 mol% ethanol: 1.08 (t, 3H), 3.55 (q, 2H).
^{13}C-NMR (100 MHz, D_2O): 60.2 (C5′), 69.8 (C3′), 77.4 (C2′), 87.7 (C4′), 99.9 (C1′), 128.4 (C5), 133.9 (C3), 140.4 (C2), 142.6 (C6), 145.6 (C4), 165.7 ($CONH_2$); Ascorbate: 62.5, 69.5, 78.2, 113.3, 174.6, 177.2. Impurities: nicotinamide: 124.2, 129.3, 136.5, 147.6, 151.7. Solvents: 16.8, 57.4 (ethanol).

Example 2: Nicotinamide-β-D-ribofuranoside citrate
5.52 g (26.3 mmol) of citric acid monohydrate were dissolved in 55 mL of DMSO while stirring. The colorless solution was cooled in an ice bath and 12 mL (86 mmol, 3.27 eq) of triethylamine were added. 73 mL of a 0.36 molar solution of $(Et_3N)_3$·citrate were prepared. 9.00 g (26.8 mmol, 1.02 eq) of nicotinamide-β-D-ribofuranoside bromide were suspended in 18 mL of DMSO. 73 mL (26.3 mmol, 1 eq) of the above produced solution were added and heated to 55 °C. The brownish solution was added to 1125 mL isopropanol, wherein a white suspension was produced. The solid was isolated by filtration and dried at 35 °C. Nicotinamide-β-D-ribofuranoside citrate was isolated as a light-yellow powder (6.32 g, 75%).
3.22 g of crude nicotinamide-β-D-ribofuranoside citrate were dissolved in a solution of 16 mL of methanol and 2 mL of water. The formed solution was dropped to 220 mL of isopropanol, wherein a white suspension was produced. The solid was isolated by filtration, washed with isopropanol and dried in vacuo at 35 °C. Nicotinamide-β-D-ribofuranoside citrate was isolated as a white powder (2.67 g, 83%). IC: Residual bromide 0.19%.

^{1}H-NMR (400 MHz, D_2O): 2.54-2.68 (m, 4H, CH_2, citrate), 3.82 (dd, 3H, *J* = 13.0, 3.5 Hz, H5′A), 3.97 (dd, 3H, *J* = 13.0, 2.9 Hz, H5′B), 4.26-4.29 (m, 3H, H3′), 4.40 (q, 3H, *J* = 3.3 Hz, H4′), 4.44 (t, 3H, *J* = 4.7 Hz, H2′), 6.17 (d, 3H, *J* = 4.4 Hz, H1′), 8.21 (dd, 3H, *J* = 8.2, 6.3 Hz, H5), 8.90 (d, 3H, *J* = 8.2 Hz, H4), 9.20 (d, 3H, *J* = 6.3 Hz, H6), 9.52 (s, 3H, H2). Impurities: 6 mol% nicotinamide: 7.50 (dd, 1H), 8.15 (m, 1H), 8.61 (d, 1H), 8.82 (s, 1H); 1.5 mol% Et_3N salt: 1.19 (t, 9H), 3.11 (q, 6H). Solvents: 31 mol% methanol: 3.26 (s, 3H); 18 mol% isopropanol: 1.07 (d, 6H), 3.92 (m, 1H).
^{13}C-NMR (100 MHz, D_2O): 44.6 (CH_2, citrate), 60.2 (C5′), 69.7 (C3′), 74.4 (C3, citrate), 77.4 (C2′), 87.7 (C4′), 99.9 (C1′), 128.4 (C5), 133.9 (C3), 140.4 (C2), 142.6 (C6), 145.6 (C4), 165.7 ($CONH_2$), 176.9 (2x COO, citrate), 180.0 (COO, citrate). Impurities: Nicotinamide: 124.3, 136.7, 147.5, 151.6; Et_3N salt: 8.2, 46.6. Solvents: 48.9 (methanol); 23.7, 64.2 (isopropanol).

Example 3: **Salt metathesis experiments where spontaneous crystallization of nicotinamide-β-D-ribofuranoside bromide occurred**

Nicotinamide-β-D-ribofuranoside formiate

1.40 mL (36 mmol, 1 eq) of formic acid were dissolved in 10 mL of methanol while stirring. The colorless solution was cooled in an ice bath and 5.0 mL (36 mmol, 1 eq) of triethylamine were added. 15 mL of a 2.40 M solution of Et_3N·formiate were prepared. 10.0 g of nicotinamide-β-D-ribofuranoside bromide (29.8 mmol) were suspended in 25 mL of methanol. 12.5 mL (30 mmol, 1 eq) of the triethylammonium formiate solution prepared according to the above procedure were added and the suspension heated to 50 °C until all solid dissolved, generating a slightly yellow solution. By storing this at 4 °C for 16 h, crystallization occurred. The formed solid was filtered and washed with ethanol. In the mother liquor additional crystals appeared and were filtered likewise. The combined solids were dried in vacuum at 35 °C, obtaining 6.21 g (62%) of white crystalline nicotinamide-β-D-ribofuranoside bromide.

^{1}H-NMR (400 MHz, D_2O): 3.88 (dd, 1H, *J* = 12.9, 3.6 Hz, H5′A), 4.02 (dd, 1H, *J* = 12.9, 2.9 Hz, H5′B), 4.33-4.36 (m, 1H, H3′), 4.45 (q, 1H, *J* = 3.6 Hz, H4′), 4.52 (t, 1H, *J* = 4.7 Hz, H2′), 6.24 (d, 1H, *J* = 4.4 Hz, H1′), 8.28 (dd, 1H, *J* = 8.0, 6.3 Hz, H5), 8.96 (d, 1H, *J* = 7.7 Hz, H4), 9.26 (d, 1H, *J* = 6.2 Hz, H6), 9.57 (s, 1H, H2). Impurities: 1 mol% nicotinamide; 0.3 mol% Et_3N salt: 1.26 (t, 9H), 3.18 (q, 6H). Solvents: 2.4 mol% methanol: 3.31 (s, 3H).
^{13}C-NMR (100 MHz, D_2O): 60.3 (C5′), 69.8 (C3′), 77.4 (C2′), 87.7 (C4′), 100.0 (C1′), 128.6 (C5), 134.0 (C3), 140.4 (C2), 142.7 (C6), 145.7 (C4), 165.8 ($CONH_2$). Solvents: 49.0 (methanol).

Experiments with maleic acid and salicylic acid behaved similarly.

Example 4: Nicotinamide-β-D-ribofuranoside D-glucuronate

5.10 g (26 mmol, 1 eq) of D-glucuronic acid were suspended in 15 mL of methanol while stirring. The colorless suspension was cooled in an ice bath and 3.60 mL (25.8 mmol, 0.99 eq) of triethylamine were added. 19.5 mL of a 1.32 molar solution of Et_3N·D-glucuronate were prepared. 5.00 g (14.9 mmol) of nicotinamide-β-D-ribofuranoside bromide were dissolved while stirring in 3.0 mL of water at RT, and 10 mL of methanol were added. 11.3 mL (14.9 mmol, 1 eq) of the above prepared solution of triethylammonium D-glucuronate were added. The clear yellowish solution was dropped slowly to 455 mL of *n*-butanol, wherein a white suspension was produced. The suspension was stirred for further five h at RT. The product was filtered, washed with isopropanol, and dried in vacuo at 35 °C. Nicotinamide-β-D-ribofuranoside D-glucuronate (6.64 g, 99 %) was dissolved in 6.6 mL of water and diluted with 33 mL of methanol. The yellowish solution was dropped to 550 mL of *n*-butanol, wherein a white suspension was produced. The suspension was filtered, the residue was washed with isopropanol and dried at 35 °C. Nicotinamide-β-D-ribofuranoside D-glucuronate was isolated as a white powder (5.19 g, 78%). Mp. 66–76 °C; residual bromide 1.43% (IC).

^{1}H-NMR (400 MHz, D_2O): NR: 3.88 (dd, 1H, *J* = 13.0, 3.5 Hz, H5′A), 4.03 (dd, 1H, *J* = 13.0, 2.9 Hz, H5′B), 4.32-4.35 (m, 1H, H3′), 4.46 (q, 1H, *J* = 3.4 Hz, H4′), 4.49 (t, 1H, *J* = 4.8 Hz, H2′), 6.23 (d, 1H, *J* = 4.4 Hz, H1′), 8.26 (dd, 1H, *J* = 8.1, 6.3 Hz, H5), 8.96 (dt, 1H, *J* = 8.1, 1.4 Hz, H4), 9.25 (d, 1H, *J* = 6.3 Hz, H6), 9.58 (s, 1H, H2); GlcUA (anomeric mixture): 3.21-3.27 (m), 3.44-3.49 (m), 3.50-3.57 (m), 3.65-3.71 (m), 4.00 (t), 4.60 (d, *J* = 7.9 Hz, β-anomer), 5.20 (d, *J* = 3.8 Hz, α-anomer). Impurities: 2 mol% nicotinamide: 7.61 (dd, 1H), 8.69 (d, 1H); 0.9 mol% Et_3N salt: 1.25 (t, 9H), 3.17 (q, 6H). Solvents: 23 mol% methanol: 3.31 (s, 3H); 5.7 mol% butanol: 0.86 (t, 3H, H4), 1.30 (m, 2H, H3), 1.48 (m, 2H, H2).
^{13}C-NMR (100 MHz, D_2O): NR: 60.2 (C5′), 69.8 (C3′), 77.4 (C2′), 87.7 (C4′), 99.9 (C1′), 128.4 (C5), 133.9 (C3), 140.4 (C2), 142.6 (C6), 145.6 (C4), 165.8 ($CONH_2$); GlcUA: 71.3, 71.7, 71.8, 72.0, 72.5, 74.0, 75.5, 76.1, 92.1, 95.9, 175.7, 176.7. Solvents: 48.9 (methanol); 13.1, 18.4, 33.4, 61.5 (n-butanol).

Example 5: Salt metathesis experiments where nicotinamide-β-D-ribofuranoside bromide crystallized during precipitation in alcohol or during addition to longer chain alcohols

Nicotinamide-β-D-ribofuranoside L-lactate

2.34 g (26 mmol) of L-lactic acid were dissolved in 10 mL of methanol while stirring. The almost colorless solution was cooled in an ice bath and 3.64 mL (26 mmol, 1 eq) of triethylamine added. 15 mL of a 1.73 molar solution of Et_3N·L-lactate were prepared. 5.80 g (17.3 mmol) nicotinamide-β-D-ribofuranoside bromide were suspended in 20 mL methanol. 10 mL (17.3 mmol, 1 eq) of the triethylammonium L-lactate solution prepared according to the above instructions were added and the suspension heated to 50 °C until all solid dissolved. The slightly yellowish, viscous solution was slowly dropped to 400 mL of isopropanol, producing a white, crystalline suspension. The solid was filtered after a few h of stirring, washed with isopropanol and dried in vacuo at 35 °C. A white crystalline powder was obtained (4.27 g, 73 %). NMR showed that nicotinamide-β-D-ribofuranoside bromide had crystallized. IC: 24.4 % bromide.

^{1}H-NMR (400 MHz, D_2O): 3.87 (dd, 1H, *J* = 12.9, 3.6 Hz, H5′A), 4.01 (dd, 1H, *J* = 12.9, 2.9 Hz, H5′B), 4.33-4.36 (m, 1H, H3′), 4.44 (q, 1H, *J* = 3.4 Hz, H4′), 4.50 (t, 1H, *J* = 4.7 Hz, H2′), 6.22 (d, 1H, *J* = 4.4 Hz, H1′), 8.25 (dd, 1H, *J* = 8.1, 6.3 Hz, H5), 8.95 (dt, 1H, *J* = 8.1, 1.5 Hz, H4), 9.24 (d, 1H, *J* = 6.3 Hz, H6), 9.56 (s, 1H, H2). Impurities: < 1 mol% nicotinamide; 0.3 mol% Et_3N salt: 1.26 (t, 9H), 3.17 (q, 6H). Solvents: 1.5 mol% isopropanol: 1.11 (d, 6H).
^{13}C-NMR (100 MHz, D_2O): 60.2 (C5′), 69.8 (C3′), 77.4 (C2′), 87.7 (C4′), 100.0 (C1′), 128.5 (C5), 134.0 (C3), 140.4 (C2), 142.7 (C6), 145.7 (C4), 165.8 ($CONH_2$). Solvents: 23.8 (iso-propanol).

Experiments with malonic acid, methanesulfonic acid and sorbic acid behaved similarly.

Example 6: Nicotinamide-β-D-ribofuranoside L-malate

3.50 g (26 mmol) of L-malic acid were dissolved in 10 mL of methanol while stirring. The almost colorless solution was cooled in an ice bath and 7.30 mL (52 mmol, 2 eq) of triethylamine were added. 18 mL of a 1.45 molar solution of $(Et_3N)_2$·L-malate were prepared. 5.00 g (14.9 mmol) of nicotinamide-β-D-ribofuranoside bromide were suspended in 15 mL of methanol. 10.3 mL (14.9 mmol, 1 eq) of the triethylammonium L-malate solution prepared according to the above instructions were added and the suspension heated to 50 °C until all solid dissolved. The slightly yellowish, viscous solution (28 mL) was slowly dropped to 280 mL of isopropanol, producing a white, flaky suspension. The solid was filtered after a few hours of stirring, washed with isopropanol and dried in vacuum at 35 °C. Nicotinamide-β-D-ribofuranoside L-malate was isolated as a white powder (4.51 g, quantitative yield). 1.80 g of crude nicotinamide-β-D-ribofuranoside L-malate were dissolved in 1.1 mL of water and diluted with 9 mL of methanol. The solution was slowly dropped to 125 mL of isopropanol, wherein a white flocculent suspension was produced. After a few hours, the solid was isolated by filtration, washed with isopropanol and dried in vacuo at 35 °C. Nicotinamide-β-D-ribofuranoside L-malate was isolated as a white powder (1.46 g, 81%). Mp: 63–68 °C. IC: Residual bromide 2.18%.

^{1}H-NMR (400 MHz, D_2O): 2.35 (dd, 1H, *J* = 15.6, 9.5 Hz, CH_2, malate), 2.63 (dd, 1H, *J* = 15.6, 3.4 Hz, CH_2, malate), 3.83 (dd, 2H, *J* = 12.9, 3.5 Hz, H5′A), 3.98 (dd, 2H, *J* = 12.9, 2.9 Hz, H5′B), 4.22 (dd, 1H, *J* = 9.5, 3.4 Hz, CHOH, malate), 4.27-4.30 (m, 2H, H3′), 4.41 (q, *J* = 3.3 Hz, 2H, H4′), 4.44 (t, 2H, *J* = 4.8 Hz, H2′), 6.18 (d, 2H, *J* = 4.4 Hz, H1′), 8.21 (dd, 2H, 8.1, 6.1 Hz, H5), 8.91 (dt, 2H, *J* = 8.1, 1.5 Hz, H4), 9.20 (d, 2H, *J* = 6.1 Hz, H6), 9.53 (s, 2H, H2). Impurities: 22 mol% nicotinamide: 7.49 (dd, 1H), 8.14 (m, 1H), 8.60 (d, 1H), 8.81 (s, 1H); 0.45 mol% Et_3N salt: 1.19 (t, 9H), 3.11 (q, 6H). Solvents: 16 mol% methanol: 3.26 (s, 3H); 17.3 mol% iso-propanol: 1.08 (d, 6H), 3.92 (m, 1H).
^{13}C-NMR (100 MHz, D_2O): 41.8 (CH_2, malate), 60.2 (C5′), 69.8 (CHOH, malate), 69.8 (C3′), 77.4 (C2′), 87.7 (C4′), 99.9 (C1′), 128.4 (C5), 133.9 (C3), 140.4 (C2), 142.6 (C6), 145.6 (C4), 165.7 ($CONH_2$), 178.6

(COO, malate), 180.4 (COO, malate). Impurity nicotinamide: 124.3, 136.6, 147.5, 151.6. Solvents: 48.9 (methanol); 23.7, 64.2 (iso-propanol).

Example 7: Nicotinamide-β-D-ribofuranoside L-tartrate

3.90 g (26.1 mmol) of L-tartaric acid were dissolved in 10 mL of methanol while stirring. The almost colorless solution was cooled in an ice bath and 7.30 mL (52.2 mmol, 2 eq) of triethylamine were added. 18 mL of a 1.44 molar solution of $(Et_3N)_2$·L-tartrate were prepared. 9.65 g (28.8 mmol, 2 eq) of nicotinamide-β-D-ribofuranoside bromide were dissolved in 5.8 mL of water and the solution was diluted with 20 mL of methanol. 10 mL (14.4 mmol) of the triethylammonium L-tartrate solution prepared according to the above instructions were added. The slightly yellowish, viscous solution (40 mL) was slowly dropped to 500 mL of ethanol, producing a white, fine suspension. The solid was filtered after an hour of stirring, washed with ethanol, and dried in vacuum at 35 °C. Nicotinamide-β-D-ribofuranoside L-tartrate was isolated as a white powder (8.06 g, 85%). 7.72 g of crude nicotinamide-β-D-ribofuranoside L-tartrate were dissolved in 7.7 mL of water and diluted with 38 mL of methanol. The colorless solution was slowly dropped to 600 mL of ethanol, wherein a white flocculent suspension was produced. After an h the solid was isolated by filtration, washed with ethanol and dried in vacuo at 35 °C. Nicotinamide-β-D-ribofuranoside L-tartrate was isolated as a white powder (5.16 g, 67%). IC: Residual bromide 0.55 %.

^{1}H-NMR (400 MHz, D_2O): 3.83 (dd, 2H, *J* = 12.9, 3.5 Hz, H5′A), 3.98 (dd, 2H, *J* = 12.9, 2.8 Hz, H5′B), 4.25 (s, 2H, 2 x CHOH, tartrate), 4.29 (t, 2H, *J* = 4.7 Hz, H3′), 4.39-4.42 (m, 2H, H4′), 4.45 (t, 2H, *J* = 4.7 Hz, H2′), 6.18 (d, 2H, *J* = 4.4 Hz, H1′), 8.21 (dd, 2H, *J* = 8.1, 6.3 Hz, H5), 8.91 (dt, 2H, *J* = 8.1, 1.5 Hz, H4), 9.21 (d, 2H, *J* = 6.3 Hz, H6), 9.53 (s, 2H, H2). Impurities: 10 mol% nicotinamide: 7.51 (dd, 1H), 8.16 (m, 1H), 8.61 (d, 1H), 8.82 (s, 1H); 0.1 mol% Et_3N salt: 1.20 (t, 9H), 3.12 (q, 6H). Solvents: 3 mol% methanol: 3.27 (s, 3H); 33 mol% ethanol: 1.09 (t, 3H), 3.56 (q, 2H).
^{13}C-NMR (100 MHz, D_2O): 60.2 (C5′), 69.7 (C3′), 73.7 (2x CHOH, tartrate), 77.4 (C2′), 87.7 (C4′), 99.9 (C1′), 128.4 (C5), 133.9 (C3), 140.4 (C2), 142.6 (C6), 145.6 (C4), 165.8 ($CONH_2$), 178.0 (2 x COO, tartrate). Impurities: Nicotinamide: 124.4, 129.4, 136.9, 147.3, 151.4; Et_3N salt: 8.2, 46.6. Solvent ethanol: 16.8, 57.4.

Section S2: Preparation of acidic salts

Preparation of a solution of Et_3N·hydrogen dicarboxylate (Et_3N·HA):

The amount of dicarboxylic acid H_2A (or citric acid) (26.0 mmol) shown in the table was suspended (S) or dissolved (D) in methanol (MeOH) while stirring at RT. The yellowish suspension or solution was cooled at 0 °C and triethylamine (Et_3N) was added, which caused a slight temperature increase. In some cases, presence of solid was observed due to poor solubilization or crystallization of the Et_3N salt. In these cases, the indicated amount of water (H_2O) was added to the suspension. The pH of the still slightly yellowish solutions was 4–4.5, with the exception of the hydrogen citrate solution, where the pH was about 6. Thus, approximately x mL of y molar Et_3N·hydrogen dicarboxylate solution (Et_3N·HA) were obtained (Table S2).

Table S2: Dicarboxylates used to prepare the Et_3N·HA solutions.

**Dicarboxylate**	**Amount Acid (g)**	**MeOH (mL)**	**S / D**	**Et_3N (mL)**	**H_2O (mL)**	**x (mL)**	**y (M)**

Hydrogen citrate	5.50*	10	D	7.30	-	~19	1.38
Dihydrogen citrate	5.50*	10	D	3.65	5.5	~21.5	1.22
Hydrogen fumarate	3.02	10	S	3.64	-	~15	1.73
Hydrogen maleate	3.02	10	D	3.64	-	~15	1.73
D-Hydrogen malate	3.50	10	D	3.64	-	~15	1.73
L-Hydrogen malate	3.50	10	D	3.64	-	~15	1.73
DL-Hydrogen malate	3.50	10	D	3.64	-	~15	1.73
Hydrogen malonate	2.70	10	D	3.64	-	~14.5	1.80
H-mercaptosuccinate	3.90	10	D	3.64	-	~15	1.73
Hydrogen ketoglutarate	3.80	10	D	3.74	-	~15	1.73
Hydrogen oxalacetate	3.43	10	D	3.64	-	~14.5	1.79
Hydrogen oxalate	2.34	10	D	3.64	0.5	~14	1.85
Hydrogen succinate	3.07	10	S	3.64	-	~15	1.73
D-Hydrogen tartrate	3.92	10	D	3.64	-	~15	1.73
L-Hydrogen tartrate	3.92	10	D	3.64	-	~15	1.73
DL-Hydrogen tartrate	3.92	10	D	3.64	-	~15	1.73
meso-Hydrogen tartrate	2.25**	5	D	1.82	-	~ 7.7	1.69
Hydrogen tartronate	3.12	10	D	3.64	-	~14.5	1.79

* Citric acid monohydrate

** meso-Tartaric acid monohydrate

<u>Non-crystalline Salts</u>

Example 8: Preparation of nicotinamide-β-D-ribofuranoside dihydrogen citrate
5.50 g (26.2 mmol) of citric acid monohydrate were dissolved in 10 mL of methanol and 5.5 mL of water while stirring. The colorless solution was cooled in an ice bath and 3.65 mL (26.2 mmol, 1 eq) of triethylamine added. 21.5 mL of a 1.22 molar solution of Et_3N·dihydrogen citrate were prepared. 0.41 g (1.22 mmol) of nicotinamide-β-D-ribofuranoside bromide were suspended in 1 mL of methanol upon stirring. 1 mL (1.22 mmol, 1 eq) of the 1.22 M solution of triethylammonium dihydrogen citrate was added. The suspension was heated at reflux and was then cooled down. The colorless and clear solution was dropped into 38 mL of isopropanol. The formed suspension was filtered, and the residue was dried at room temperature in vacuo. 0.23 g (42 %) of a white hygroscopic powder were obtained, which melted during drying and was therefore not further investigated.

Example 9: Preparation of nicotinamide-β-D-ribofuranoside meso-hydrogen tartrate
2.25 g (13.0 mmol) of meso-tartaric acid monohydrate 97% were dissolved in 5 mL of methanol with stirring. The colorless solution was cooled in an ice bath and 1.82 mL (13.0 mmol, 1 eq) of triethylamine added. 7.7 mL of a 1.69 M solution of Et_3N·meso-hydrogen tartrate were prepared. 0.57 g (1.70 mmol) of nicotinamide-β-D-ribofuranoside bromide were suspended in 1 mL of methanol upon stirring. 1 mL (1.69 mmol, 1 eq) of the 1.69 molar solution of triethylammonium meso-hydrogen tartrate was added. The suspension was heated to the boiling point and was then cooled down. The formed emulsion was slightly warmed up, and the clear solution dropped into 20

mL of ethanol. The formed suspension was filtered, and the residue was dried at room temperature in vacuo. Nicotinamide-β-D-ribofuranoside meso-hydrogen tartrate was isolated as a flaky, hygroscopic powder (0.44 g, 62%).

^{1}H-NMR (400 MHz, D_2O): 3.82 (dd, 1H, *J* = 12.9, 3.5 Hz, H5′A), 3.97 (dd, 1H, *J* = 12.9, 2.8 Hz, H5′B), 4.28 (m, 1H, H3′), 4.35 (s, 2H, 2 x CHOH, meso-H-tartrate), 4.40 (m, 1H, H4′), 4.44 (t, 1H, *J* = 4.7 Hz, H2′), 6.18 (d, 1H, *J* = 4.5 Hz, H1′), 8.21 (t, 1H, *J* = 7.0 Hz, H5), 8.91 (d, 1H, *J* = 8.2 Hz, H4), 9.20 (d, 1H, *J* = 6.2 Hz, H6), 9.53 (s, 1H, H2). Impurities: 5 mol% nicotinamide: 7.65 (dd, 1H), 8.33 (d, 1H), 8.67 (d, 1H); 10 mol% Et_3N salt: 1.20 (t, 9H), 3.12 (q, 6H). Solvents: 16 mol% methanol: 3.26 (s, 3H); 40 mol% ethanol: 1.09 (t, 3H), 3.56 (q, 2H).
^{13}C-NMR (100 MHz, D_2O): 60.2 (C5′), 69.7 (C3′), 73.7 (2 x CHOH, meso-H-tartrate), 77.4 (C2′), 87.7 (C4′), 99.9 (C1′), 128.4 (C5), 133.9 (C3), 140.4 (C2), 142.6 (C6), 145.6 (C4), 165.8 ($CONH_2$), 175.7 (2 x COO, meso-H-tartrate). Impurities: Nicotinamide: 125.0, 138.6, 146.0, 149.8; Et_3N salt: 8.2, 46.6. Solvents: 48.9 (methanol); 16.8, 57.4 (ethanol).

Crystalline Salts from Nicotinamide-β-D-ribofuranoside bromide

Example 10: Preparation of nicotinamide-β-D-ribofuranoside L-hydrogen tartrate from nicotinamide-β-D-ribofuranoside bromide using various ammonium L-hydrogen tartrate salts for salt metathesis

Example 10a: Use of triethylammonium·L-hydrogen tartrate
3.92 g (26.1 mmol) of L-tartaric acid were dissolved in 10 mL of methanol while stirring. The colorless solution was cooled in an ice bath, and 3.64 mL triethylamine (26.1 mmol, 1 eq) were added. The pH of the slightly yellowish solution was around 4–4.5. 15 mL of a 1.73 M solution of Et_3N·L-hydrogen tartrate were prepared. 5.80 g (17.3 mmol) of nicotinamide-β-D-ribofuranoside bromide were dissolved while stirring in 3.5 mL water at room temperature, and 10 mL of methanol were added. 10 mL (17.3 mmol, 1 eq) of the above prepared solution of triethylammonium L-hydrogen tartrate were added to the clear colorless solution. The formed white suspension was stirred for a further hour at room temperature. The product was filtered, washed with methanol and dried in vacuo at 35 °C. Nicotinamide-beta-D-ribofuranoside·L-hydrogen tartrate was isolated as a white, crystalline powder (6.62 g, 95%). Mp: 129–130°C; IC: Residual bromide 0.20 %.

^{1}H-NMR (400 MHz, D_2O): 3.82 (dd, 1H, *J* = 12.9, 3.5 Hz, H5′A), 3.96 (dd, 1H, *J* = 12.9, 2.9 Hz, H5′B), 4.28 (t, 1H, *J* = 4.6 Hz, H3′), 4.38 – 4.46 (m, 2H, H2′, H4′), 4.41 (s, 2H, 2 x CHOH, H-tartrate), 6.17 (d, 1H, *J* = 4.4 Hz, H1′), 8.20 (dd, 1H, *J* = 8.1, 6.3 Hz, H5), 8.90 (dt, 1H, *J* = 8.1, 1.5 Hz, H4), 9.19 (d, 1H, *J* = 6.2 Hz, H6), 9.52 (s, 1H, H2). Impurities: < 1 mol% nicotinamide; 1.2 mol% Et_3N salt: 1.19 (t, 9H), 3.11 (q, 6H). Solvents: 7.3 mol% methanol: 3.25 (s, 3H).
^{13}C-NMR (100 MHz, D_2O): 60.2 (C5′), 69.7 (C3′), 72.8 (2x CHOH, H-tartrate), 77.4 (C2′), 87.6 (C4′), 99.9 (C1′), 128.4 (C5), 133.9 (C3), 140.4 (C2), 142.6 (C6), 145.6 (C4), 165.8 ($CONH_2$), 176.3 (2 x COO, H-tartrate). Impurity: 8.2, 46.6 (Et_3N). Solvents: 48.9 (methanol).

Example 10b: Use of tributylammonium·L-hydrogen tartrate
3.92 g (26.1 mmol) of L-tartaric acid were dissolved in 10 mL of methanol with stirring. The colorless solution was cooled in an ice bath and 6.3 mL tributylamine (26.0 mmol, 1 eq) added. The pH of the slightly yellowish solution was around 4. In this manner 17.5 mL of a 1.49 molar solution of tributylammonium L-hydrogen tartrate was prepared. 5.80 g (17.3 mmol) of nicotinamide-β-D-ribofuranoside bromide were dissolved while stirring in 3.5 mL water at room temperature, and

10 mL of methanol were added. 11.6 mL (17.3 mmol, 1 eq) of the above prepared solution of tributylammonium L-hydrogen tartrate were added to the clear colorless solution. White product immediately starts crystallizing, and the suspension was stirred for a further hour at room temperature. The product was filtered, washed with methanol and dried in vacuum at 35 °C. Nicotinamide-beta-D-ribofuranoside·L-hydrogen tartrate was isolated as a white, crystalline powder (6.37 g, 91%). Mp. 128 °C; IC: Residual bromide 0.62%.

^{1}H-NMR (400 MHz, D_2O): Analogous to Example 10a. Impurities: < 1 mol% nicotinamide, 2.7 mol% Bu_3N salt: 0.84 (t, 9H), 1.28 (m, 6H), 1.58 (m, 6H), 3.04 (q, 6H); solvents: 3.7 mol% methanol: 3.25 (s, 3H).
^{13}C-NMR (100 MHz, D_2O): Analogous to Example 10a.

Example 10c: Use of tetrabutylammonium·L-hydrogen tartrate
3.92 g (26.1 mmol) of L-tartaric acid were dissolved in 10 mL methanol while stirring. The colorless solution was cooled in an ice bath and 17.1 mL (26.1 mmol, 1 eq) of a 40% solution of tetrabutylammonium hydroxide in water were added. The pH of the slightly yellowish solution was around 4. 29 mL of a 0.9 M solution of tetrabutylammonium L-hydrogen tartrate were prepared. 5.80 g (17.3 mmol) of nicotinamide-β-D-ribofuranoside bromide were dissolved while stirring in 3.5 mL of water at room temperature, and 10 mL of methanol were added. 19.3 mL (17.3 mmol, 1 eq) of the above prepared solution of tetrabutylammonium L-hydrogen tartrate were added to the clear colorless solution. The formed white suspension was stirred for a further hour at room temperature. The product was filtered, washed with methanol and dried in vacuum at 35 °C. Nicotinamide-beta-D-ribofuranoside·L-hydrogen tartrate was isolated as a white, crystalline powder (5.60 g, 80%). Mp. 129–130°C; IC: Residual bromide 0.13%.

^{1}H-NMR (400 MHz, D_2O): Analogous to Example 10a. Impurities: < 1 mol% nicotinamide; 0.35 mol% Bu_4N salt: 0.36 (t, 9H), 1.27 (m, 6H), 1.56 (m, 6H), 3.11 (q, 6H); solvents: 2.4 mol% methanol: 3.26 (s, 3H).
^{13}C-NMR (100 MHz, D_2O): Analogous to Example 10a.

Example 11: Preparation of nicotinamide-β-D-ribofuranoside DL-hydrogen tartrate from nicotinamide-β-D-ribofuranoside bromide

NR^+ DL-hydrogen tartrate was prepared analogously to Example 10a and isolated as a white, crystalline powder (6.30 g, 90%). Mp: 112–114°C; IC: Residual bromide 0.1%.

^{1}H-NMR (400 MHz, D_2O): 3.82 (dd, 1H, *J* = 12.9, 3.6 Hz, H5′A), 3.97 (dd, 1H, *J* = 12.9, 2.8 Hz, H5′B), 4.28 (t, 1H, *J* = 4.6 Hz, H3′), 4.38–4.46 (m, 2H, H2′, H4′), 4.42 (s, 2H, 2 x CHOH, H-tartrate), 6.17 (d, 1H, *J* = 4.5 Hz, H1′), 8.20 (dd, 1H, *J* = 8.1, 6.2 Hz, H5), 8.90 (dt, 1H, *J* = 8.1, 1.4 Hz, H4), 9.19 (d, 1H, *J* = 6.3 Hz, H6), 9.52 (s, 1H, H2). Impurities: < 1 mol% nicotinamide; 0.7 mol% Et_3N salt: 1.19 (t, 9H), 3.11 (q, 6H). Solvents: 3.3 mol% methanol: 3.25 (s, 3H).
^{13}C-NMR (100 MHz, D_2O): 60.2 (C5′), 69.7 (C3′), 72.8 (2 x CHOH, H-tartrate), 77.4 (C2′), 87.7 (C4′), 99.9 (C1′), 128.4 (C5), 133.9 (C3), 140.4 (C2), 142.6 (C6), 145.6 (C4), 165.8 (CONH2), 176.3 (2 x COO, H-tartrate). Impurity: 8.2 (Et_3N). Solvents: 48.9 (methanol).

Example 12: Preparation of nicotinamide-β-D-ribofuranoside L-hydrogen malate from nicotinamide-β-D-ribofuranoside bromide using various ammonium L-hydrogen malate salts for salt metathesis

Example 12a: Use of triethylammonium·L-hydrogen malate

3.50 g (26.1 mmol) of L-malic acid were dissolved in 10 mL methanol with stirring. The solution was cooled in an ice bath and 3.64 mL of triethylamine (26.1 mmol, 1 eq) added. The pH of the slightly yellowish solution was around 4.5. 15 mL of a 1.73 molar solution of Et_3N·L-hydrogen malate were prepared. 5.80 g (17.3 mmol) of nicotinamide-β-D-ribofuranoside bromide were suspended in 10 mL of methanol upon stirring. 10 mL (17.3 mmol, 1 eq) of the 1.73 molar solution of triethylammonium L-hydrogen malate were added. The suspension was heated until the solids dissolved completely. After cooling, a white solid precipitated. The suspension was stirred for 30 min and then filtered. The residue was washed with methanol and dried in vacuo at 35 °C. Nicotinamide-beta-D-ribofuranoside·L-hydrogen malate was isolated as a white crystalline powder (4.15 g, 62%). Mp: 116.5–117 °C. IC: Residual bromide 0.10%.

^{1}H-NMR (400 MHz, D_2O): 2.53 (dd, 1H, *J* = 16.0, 8.1 Hz, CH_2, H-malate), 2.72 (dd, 1H, *J* = 16.0, 4.3 Hz, CH_2, H-malate), 3.81 (dd, 1H, *J* = 12.9, 3.5 Hz, H5′A), 3.96 (dd, 1H, *J* = 12.9, 2.8 Hz, H5′B), 4.27 (m, 1H, H3′), 4.28 (m, 1H, CHOH, H-malate), 4.39 (q, *J* = 3.3 Hz, 1H, H4′), 4.43 (t, 1H, *J* = 4.7 Hz, H2′), 6.17 (d, 1H, *J* = 4.5 Hz, H1′), 8.20 (t, 1H, *J* = 6.9 Hz, H5), 8.90 (dt, 1H, *J* = 8.1, 1.4 Hz, H4), 9.19 (d, 1H, *J* = 6.3 Hz, H6), 9.52 (s, 1H, H2). Impurities: < 1 mol% nicotinamide; 0.7 mol% Et_3N salt: 1.19 (t, 9H), 3.11 (q, 6H). Solvents: 6.3 mol% methanol: 3.25 (s, 3H).

^{13}C-NMR (100 MHz, D_2O): 40.0 (CH_2, H-malate), 60.2 (C5′), 68.5 (CHOH, H-malate), 69.7 (C3′), 77.4 (C2′), 87.7 (C4′), 99.9 (C1′), 128.4 (C5), 133.9 (C3), 140.4 (C2), 142.6 (C6), 145.6 (C4), 165.7 ($CONH_2$), 176.3 (COO, H-malate), 179.0 (COO, H-malate). Impurity Et_3N salt: 8.2. Solvent: 48.9 (methanol).

Example 12b: Use of tetrabutylammonium·L-hydrogen malate

3.50 g (26.1 mmol) of L-malic acid were dissolved in 10 mL of methanol while stirring. The colorless solution was cooled in an ice bath and 6.23 mL of tributylamine (26.1 mmol, 1 eq) were added. The pH of the slightly yellowish solution was around 5. 17.5 mL of a 1.49 M solution of tributylammonium L-hydrogen malate were prepared. 5.80 g (17.3 mmol) of nicotinamide-β-D-ribofuranoside bromide were suspended in 17.5 mL of methanol upon stirring. 11.6 mL (17.3 mmol, 1 eq) of the above prepared solution of tributylammonium L-hydrogen malate were added. The suspension was heated until the solids dissolved completely. A white solid crystallized upon cooling. The suspension was stirred for 3 h and then filtered. The residue was washed with methanol and dried in vacuo at 35 °C. Nicotinamide-beta-D-ribofuranoside·L-hydrogen malate was isolated as a white crystalline powder (4.89 g, 73%). Mp. 115.5 °C; IC: Residual bromide 0.64%.

^{1}H-NMR (400 MHz, D_2O): Analogous to Example 12a. Impurities: < 1 mol% nicotinamide; 0.2 mol% Bu_3N salt; 2 mol% methanol.

^{13}C-NMR (100 MHz, D_2O): Analogous to Example 12a.

Example 13: The following crystalline nicotinamide-β-D-ribofuranoside salts were prepared analogously to Example 12a

NR^+ D-hydrogen malate: 4.01 g (60%) of a white, crystalline powder were obtained; Mp. 117.0-117.5 °C; IC: Residual bromide 0.90%.

^{1}H-NMR (400 MHz, D_2O): 2.54 (dd, 1H, *J* = 16.0, 8.1 Hz, CH2, H-malate), 2.72 (dd, 1H, *J* = 16.0, 4.3 Hz, CH2, H-malate), 3.81 (dd, 1H, *J* = 12.9, 3.5 Hz, H5′A), 3.96 (dd, 1H, *J* = 12.9, 2.9 Hz, H5′B), 4.27 (m, 1H, H3′), 4.28 (m, 1H, CHOH, H-malate), 4.39 (q, *J* = 3.4 Hz, 1H, H4′), 4.43 (t, 1H, *J* = 4.8 Hz, H2′), 6.17 (d, 1H, *J* = 4.4 Hz, H1′), 8.20 (t, 1H, 6.9 Hz, H5), 8.90 (d, 1H, *J* = 8.1 Hz, H4), 9.19 (d, 1H, *J* = 6.3

Hz, H6), 9.52 (s, 1H, H2). Impurities: 1 mol% nicotinamide; 0.45 mol% Et_3N salt: 1.19 (t, 9H), 3.11 (q, 6H). Solvents: 9 mol% methanol: 3.25 (s, 3H).
^{13}C-NMR (100 MHz, D_2O): 40.0 (CH_2, H-malate), 60.2 (C5′), 68.5 (CHOH, H-malate), 69.7 (C3′), 77.4 (C2′), 87.7 (C4′), 99.9 (C1′), 128.4 (C5), 133.9 (C3), 140.4 (C2), 142.6 (C6), 145.6 (C4), 165.7 ($CONH_2$), 176.3 (COO, H-malate), 179.0 (COO, H-malate). Solvents: 48.9 (methanol).

NR^+ DL-Hydrogen malate: 4.52 g (67%) of a white, crystalline powder were obtained; Mp. 108-109 °C; IC: Residual bromide 2.30%.

^{1}H-NMR (400 MHz, D_2O): 2.54 (dd, 1H, *J* = 16.0, 8.1 Hz, CH_2, H-malate), 2.72 (dd, 1H, *J* = 16.0, 4.3 Hz, CH_2, H-malate), 3.82 (dd, 1H, *J* = 12.9, 3.5 Hz, H5′A), 3.96 (dd, 1H, *J* = 12.9, 2.9 Hz, H5′B), 4.27 (m, 1H, H3′), 4.28 (m, 1H, CHOH, H-malate), 4.40 (q, *J* = 3.4 Hz, 1H, H4′), 4.43 (t, 1H, *J* = 4.8 Hz, H2′), 6.17 (d, 1H, *J* = 4.4 Hz, H1′), 8.20 (t, 1H, 6.9 Hz, H5), 8.90 (d, 1H, *J* = 7.7 Hz, H4), 9.19 (d, 1H, *J* = 6.3 Hz, H6), 9.52 (s, 1H, H2). Impurities: < 1 mol% nicotinamide; 1.4 mol% Et_3N salt: 1.19 (t, 9H), 3.11 (q, 6H). Solvents: 3.4 mol% methanol: 3.25 (s, 3H).
^{13}C-NMR (100 MHz, D_2O): 40.0 (CH_2, H-malate), 60.2 (C5′), 68.5 (CHOH, H-malate), 69.7 (C3′), 77.4 (C2′), 87.7 (C4′), 99.9 (C1′), 128.4 (C5), 133.9 (C3), 140.4 (C2), 142.6 (C6), 145.6 (C4), 165.7 ($CONH_2$), 176.3 (COO, H-malate), 179.0 (COO, H-malate). Impurity: 8.2, 46.6 (Et_3N). Solvents: 48.9 (methanol).

NR^+ D-Hydrogen tartrate: 4.90 g (70%) of a white, crystalline powder were obtained; Mp. 124-126 °C; IC: Residual bromide 0.30%.

^{1}H-NMR (400 MHz, D_2O): 3.82 (dd, 1H, *J* = 12.9, 3.5 Hz, H5′A), 3.97 (dd, 1H, *J* = 12.9, 2.9 Hz, H5′B), 4.28 (t, 1H, *J* = 4.6 Hz, H3′), 4.38 – 4.46 (m, 2H, H2′, H4′), 4.42 (s, 2H, 2 x CHOH, H-tartrate), 6.17 (d, 1H, *J* = 4.4 Hz, H1′), 8.20 (dd, 1H, *J* = 8.1 , 6.2 Hz, H5), 8.90 (dt, 1H, *J* = 8.1, 1.4 Hz, H4), 9.19 (d, 1H, *J* = 6.2 Hz, H6), 9.52 (s, 1H, H2). Impurities: 1 mol% nicotinamide; 1.6 mol% Et_3N salt: 1.19 (t, 9H), 3.11 (q, 6H). Solvents: 11 mol% methanol: 3.25 (s, 3H).
^{13}C-NMR (100 MHz, D_2O): 60.2 (C5′), 69.7 (C3′), 72.8 (2 x CHOH, H-tartrate), 77.4 (C2′), 87.7 (C4′), 99.9 (C1′), 128.4 (C5), 133.9 (C3), 140.4 (C2), 142.6 (C6), 145.6 (C4), 165.8 ($CONH_2$), 176.3 (2 x COO, H-tartrate). Impurity: 8.2, 46.6 (Et_3N). Solvents: 48.9 (methanol).

NR^+ D-hydrogen tartrate monohydrate:
2.0 g nicotinamide-β-D-ribofuranoside D-hydrogen tartrate prepared in the example above were dissolved in 9 ml water. 70 ml methanol were added to the colorless solution with stirring. After approximately one minute white crystals precipitated. One hour later the formed suspension was filtered. The residue was washed with methanol and dried in vacuo at 35 °C. 1.54 g (77 %) of a white crystalline powder of the monohydrate was obtained. Water content: 4.24 % (determined according to K. Fischer); Mp. 115-116 °C; IC: Residual bromide: < 0.01 %.

Example 14: Preparation of nicotinamide-2,3,5-tri-*O*-acetyl-β-D-ribofuranoside triflate

Example 14a: according to our method
11.55 g (94 mmol) of nicotinamide and 29.7 g (93 mmol, 1 eq) of β-D-ribofuranose 1,2,3,5-tetraacetate were dissolved upon stirring at room temperature in 750 mL of acetonitrile which has been dried over 3 Å molecular sieves. 18.2 mL (97 mmol, 1.03 eq) of trimethylsilyl triflate were added within 20 minutes. The yellow solution was stirred for 20 minutes. Subsequently, the solvent was removed in vacuo at 35 °C. The formed foam was dissolved in 300 mL of dichloromethane and 4.5 g of activated charcoal were added. The suspension was filtered and the filtrate was concentrated in vacuo.

Nicotinamide-2,3,5-tri-*O*-acetyl-β-D-ribofuranoside triflate was isolated as a yellow foam (49.5 g, quantitative yield).

^{1}H-NMR (400 MHz, D_2O): 2.10, 2.13, 2.17 (3 x s, 3 x 3H, $COCH_3$), 4.53 (m, 2H, *J* = 2.5 Hz, H5′), 4.89 (m, 1H, H4′), 5.46 (t, 1H, *J* = 5.5 Hz, H3′), 5.56 (dd, 1H, *J* = 5.5, 3.9 Hz, H2′), 6.59 (d, 1H, *J* = 3.9 Hz, H1′), 8.28 (t, 1H, *J* = 8.1 Hz, H5), 9.00 (d, 1H, *J* = 8.2 Hz, H4), 9.21 (d, 1H, *J* = 6.4 Hz, H6), 9.45 (s, 1H, H2). Impurities: 4 mol% nicotinamide, 2 mol% D-ribose-tetraacetate.
^{13}C-NMR (100 MHz, D_2O): 19.8, 19.9, 20.2 (3 x $COCH_3$), 62.6 (C5′), 69.4 (C3′), 76.4 (C2′), 82.7 (C4′), 97.3 (C1′); 114.9 + 118.1 + 121.2 + 124.4 (q, CF3); 128.7 (C5), 134.2 (C3), 140.4 (C2), 143.1 (C6), 146.2 (C4), 165.4 ($CONH_2$), 172.3, 172.4, 173.3 (3x CO).

Example 14b: for comparison
The method was carried out as described by Tanimori using a high excess of trimethylsilyl triflate, wherein the product was isolated as described above. The obtained foam contained a complex mixture of compounds. 0.43 g (3.5 mmol, 1.11 eq) of nicotinamide and 1.00 g (3.14 mmol) of β-D-ribofuranose 1,2,3,5-tetraacetate were dissolved upon stirring at room temperature in 25 mL acetonitrile which has been dried over 3 Å molecular sieves. 5.00 mL (26.8 mmol, 8.5 eq) of trimethylsilyl triflate were added within 5 minutes. The yellow solution was stirred for an h. Subsequently, the solvent was removed in vacuo at 35 °C. The reddish-brown oil was dissolved in 11 mL of dichloromethane and 0.16 g of activated charcoal were added. The suspension was stirred for 15 min, filtered, and the filtrate was concentrated. 1.72 g of a yellow-brown oil were obtained.

^{1}H-NMR (400 MHz, D_2O): Complex mixture of compounds

Crystalline Salts through NR^+-triacetate triflate

Examples 15: Deacetylation of nicotinamide-β-D-riboside-2,3,5-triacetate triflate for preparation of nicotinamide-β-D-ribofuranoside L-hydrogen tartrate

Example 15a: Deacetylation using sulfuric acid and neutralization using triethylamine
Preparation of a diluted sulfuric acid in methanol: 27 g of methanol were cooled down to 0 °C. 3.00 g (30.6 mmol) of a 96% sulfuric acid were added while stirring. 30 g of a 10% methanolic sulfuric acid were obtained.
Deacylation of nicotinamide-2,3,5-tri-*O*-acetyl-β-D-ribofuranoside triflate: 3.00 g (5.66 mmol) of nicotinamide-2,3,5-tri-*O*-acetyl-β-D-ribofuranoside triflate were dissolved in 15 mL of methanol while stirring. 5.55 g (5.66 mmol, 1 eq) of the above methanolic sulfuric acid were added. The resulting colorless solution was stirred at room temperature for three days. Control by thin-layer chromatography revealed complete deacylation and some nicotinamide impurities.
Conversion to nicotinamide-β-D-riboside L-hydrogen tartrate after neutralization with triethylamine: 1.1 mL of triethylamine (7.89 mmol, 1.4 eq) were added to the above solution. 3.3 mL (5.61 mmol, 1 eq) of a 1.7 molar methanolic solution of triethylammonium L-hydrogen tartrate were added, wherein product immediately started precipitating. Subsequently, 0.40 g of L-tartaric acid (2.6 mmol, 0.46 eq) were added. The product suspension was stored for 12 h in a refrigerator. After filtration, the obtained solid was washed with methanol and ethanol and dried in vacuo at 30°C. Nicotinamide-β-D-ribofuranoside L-hydrogen tartrate was isolated as a white crystalline powder (1.23 g, 54%). Mp. 127–128 °C.

^{1}H-NMR (400 MHz, D_2O): 3.81 (dd, 1H, *J* = 12.9, 3.6 Hz, H5′A), 3.95 (dd, 1H, *J* = 12.9, 2.9 Hz, H5′B),

4.25-4.29 (m, 1H, H3′), 4.36-4.45 (m, 2H, H4′, H2′), 4.40 (s, 2H, 2 x CHOH, H-tartrate), 6.16 (d, 1H, *J*= 4.4 Hz, H1′), 8.19 (dd, 1H, *J* = 8.1, 6.3 Hz, H5), 8.89 (dt, 1H, *J* = 8.1, 1.5 Hz, H4), 9.18 (d, 1H, *J* = 6.3 Hz, H6), 9.51 (s, 1H, H2). Impurities: 2 mol% nicotinamide: 7.83 (m, 1H), 8.54 (m, 1H), 8.76 (d, 1H), 9.00 (s, 1H); 2.9 mol% Et_3N salt: 1.18 (t, 9H), 3.10 (q, 6H). Solvents: 16 mol% methanol: 3.25 (s, 3H), 2 mol% ethanol.
^{13}C-NMR (100 MHz, D_2O): 60.2 (C5′), 69.7 (C3′), 72.8 (2x CHOH, H-tartrate), 77.4 (C2′), 87.6 (C4′), 99.9 (C1′), 128.4 (C5), 133.9 (C3), 140.4 (C2), 142.6 (C6), 145.6 (C4), 165.8 ($CONH_2$), 176.3 (2 x COO, H-tartrate). Impurities: 8.2, 46.6 (Et_3N salt). Solvents: 48.9 (methanol).

Example 15b: Deacetylation using HBr in glacial acetic acid and neutralization using triethylamine
8.0 g (15 mmol) of nicotinamide-2,3,5-tri-*O*-acetyl-β-D-ribofuranoside triflate were dissolved in 32 mL of methanol while stirring. The solution was cooled down to 0–5°C. After addition of 5.2 mL (30 mmol, 2 eq) of HBr 33% in glacial acetic acid, the solution was kept stirring at room temperature. According to control by thin-layer chromatography, the product was deacylated after 48 h. The solution was divided into two halves.
Isolation of the formed intermediate bromide: One half of the solution (20.5 mL) was seeded with nicotinamide-β-D-ribofuranoside bromide and was stirred at RT. After about 30 min a suspension was formed. The suspension was filtered and the residue was washed with methanol and ethanol, and was subsequently dried in vacuo at 30 °C. Nicotinamide-β-D-ribofuranoside bromide was isolated as a white crystalline powder (0.62 g, 25%).

^{1}H-NMR (400 MHz, D_2O): 3.87 (dd, 1H, *J* = 12.9, 3.6 Hz, H5′A), 4.01 (dd, 1H, *J* = 12.9, 2.9 Hz, H5′B), 4.30–4.35 (m, 1H, H3′), 4.44 (q, 1H, *J* = 3.4 Hz, H4′), 4.50 (t, 1H, *J* = 4.7 Hz, H2′), 6.22 (d, 1H, *J* = 4.4 Hz, H1′), 8.25 (dd, 1H, *J* = 8.1, 6.3 Hz, H5), 8.95 (dt, 1H, *J* = 8.1, 1.5 Hz, H4), 9.24 (d, 1H, *J* = 6.3 Hz, H6), 9.56 (s, 1H, H2). Impurities: 1 mol% nicotinamide (8.11 ppm).
^{13}C-NMR (100 MHz, D_2O): 60.2 (C5′), 69.8 (C3′), 77.4 (C2′), 87.7 (C4′), 100.0 (C1′), 128.5 (C5), 134.0 (C3), 140.4 (C2), 142.7 (C6), 145.7 (C4), 165.8 ($CONH_2$). Solvents: 23.8 (iso-propanol).

Conversion to nicotinamide-β-D-riboside L-hydrogen tartrate after neutralization with triethylamine: 1.8 mL of triethylamine (12.9 mmol, 1.7 eq) were added to the other half of the solution, wherein HBr and acetic acid were partially neutralized. 4.4 mL (7.5 mmol, 1 eq) of a 1.7 M methanolic solution of triethylammonium L-hydrogen tartrate were added to the yellowish solution, wherein product started precipitating. After filtration and washing with methanol and ethanol, and drying in vacuo at 30 °C, nicotinamide-β-D-ribofuranoside L-hydrogen tartrate was isolated as a white crystalline powder (1.62 g, 53%). Mp. 127–128 °C.

^{1}H-NMR (400 MHz, D_2O): Analogous to Example 15a. Impurities: 1 mol% nicotinamide; 3.7 mol% Et_3N salt. Solvents: 12.5 mol% methanol.
^{13}C-NMR (100 MHz, D_2O): Analogous to Example 15a.

Example 15c: Deacetylation using hydrochloric acid and neutralization using triethylamine
8.00 g (15.1 mmol) of nicotinamide-2,3,5-tri-*O*-acetyl-β-D-ribofuranoside triflate were dissolved in 32 mL of methanol while stirring. 5.5 mL (31 mmol, 2 eq) of a 5.6 M solution of hydrogen chloride in ethanol were added while cooling. The resulting colorless solution was stirred at RT overnight. Control by thin-layer chromatography revealed complete deacylation and some nicotinamide impurities. The solution was divided into two halves.
Isolation of the formed intermediate chloride: One half of the solution (20.5 mL) was seeded with

nicotinamide-β-D-ribofuranoside chloride and stirred at room temperature. After about 2 h, a very thin suspension was formed, which was stored overnight in the refrigerator. The thin suspension was filtered, the residue was washed with ethanol and subsequently dried in vacuo at 30 °C. Only 0.05 g (2%) of a white crystalline powder of nicotinamide-beta-D-ribofuranoside chloride were obtained. Mp. 104 °C.

^{1}H-NMR (400 MHz, D_2O): 3.85 (dd, 1H, *J* = 12.9, 3.5 Hz, H5′A), 3.99 (dd, 1H, *J* = 12.9, 2.9 Hz, H5′B), 4.27–4.35 (m, 1H, H3′), 4.42 (q, 1H, *J* = 3.5 Hz, H4′), 4.47 (t, 1H, *J* = 4.7 Hz, H2′), 6.20 (d, 1H, *J* = 4.5 Hz, H1′), 8.24 (d, 1H, *J* = 6.9 Hz, H5), 8.93 (d, 1H, *J* = 8.1, H4), 9.22 (d, 1H, *J* = 6.3 Hz, H6), 9.54 (s, 1H, H2). Impurities: 3 mol% nicotinamide (8.07 ppm).
^{13}C-NMR (100 MHz, D_2O): 60.2 (C5′), 69.8 (C3′), 77.4 (C2′), 87.7 (C4′), 100.0 (C1′), 128.5 (C5), 134.0 (C3), 140.4 (C2), 142.7 (C6), 145.7 (C4), 165.8 ($CONH_2$).

Conversion to nicotinamide-β-D-riboside L-hydrogen tartrate after neutralization with triethylamine: 1.6 mL of triethylamine (11.5 mmol, 1.5 eq) were added dropwise to the other half of the above solution. 4.4 mL (7.5 mmol, 1 eq) of a 1.7 molar methanolic solution of triethylammonium L-hydrogen tartrate were added to the almost colorless solution, wherein product started precipitating. The suspension was stirred for a few hours and then stored for 16 h in a refrigerator. After filtration, the obtained solid was washed with methanol and ethanol and dried in vacuo at 30 °C. 1.82 g (60%) of a white crystalline powder of nicotinamide-beta-D-ribofuranoside L-hydrogen tartrate were obtained. Mp. 124–125 °C.

^{1}H-NMR (400 MHz, D_2O): Analogous to Example 15a. Impurities: < 1 mol% nicotinamide; 3.8 mol% Et_3N salt. Solvents: 8.3 mol% methanol.
^{13}C-NMR (100 MHz, D_2O): Analogous to Example 15a.

Example 15d: Deacetylation using triethylamine
Deacylation of nicotinamide-D-riboside-2,3,5-triacetate triflate: 3.00 g (5.66 mmol) of the triflate were dissolved in 18 mL methanol while stirring. 0.8 mL (5.7 mmol, 1 eq) of triethylamine were added to the solution cooled down to 0 °C. After stirring for 4 days at 0–5°C, thin-layer control showed complete conversion.
Conversion to nicotinamide-β-D-riboside L-hydrogen tartrate: The brown-orange solution obtained in the step above was warmed to RT. Subsequently, 0.86 g (5.7 mmol, 1 eq) of L-tartaric acid were added, and a suspension was formed. The product suspension was cooled down to 0 °C and stirred. After storage in a refrigerator for 12 h, the suspension was filtered, the obtained solid washed with 5 mL isopropanol and dried in vacuo at 30 °C. 1.44 g (63%) of a brownish-yellow crystalline powder were obtained. Mp. 127 °C.

^{1}H-NMR (400 MHz, D_2O): Analogous to Example 15a. Impurities: 2 mol% nicotinamide; 1.9 mol% Et_3N salt. Solvents: 13.3 mol% methanol, 2.8 mol% isopropanol.
^{13}C-NMR (100 MHz, D_2O): Analogous to Example 15a.

Examples 16: Deacetylation of nicotinamide-β-D-riboside-2,3,5-triacetate triflate for preparation of nicotinamide-β-D-ribofuranoside L-hydrogen malate

Example 16a: Deacetylation using sulfuric acid and neutralization using triethylamine
Preparation of a diluted sulfuric acid in methanol: 27 g of methanol were cooled down to 0 °C. 3.00 g (30.6 mmol) of a 96% sulfuric acid were added while stirring. 30 g of a 10% methanolic sulfuric

acid were obtained.

Deacylation of nicotinamide-2,3,5-tri-*O*-acetyl-β-D-ribofuranoside triflate: 8.00 g (15.1 mmol) of nicotinamide-2,3,5-tri-*O*-acetyl-β-D-ribofuranoside triflate were dissolved in 10 mL of methanol while stirring. 15.6 g (15.9 mmol, 1.05 eq) of the above methanolic sulfuric acid were added. The resulting colorless solution was stirred at RT for 16 h. Control by thin-layer chromatography revealed complete deacylation and some nicotinamide impurities.

Conversion to nicotinamide-β-D-riboside L-hydrogen malate after neutralization with triethylamine: 3.0 mL (21.5 mmol, 1.4 eq) of triethylamine were added dropwise to the above solution. 2.03 g (15.1 mmol, 1 eq) of L-malic acid were added, followed by additional 2.5 mL (18 mmol, 1.2 eq) of triethylamine. The product soon started precipitating. The suspension was stirred for a few h and then stored for 12 h in a refrigerator. After filtration, the obtained solid was washed with methanol and ethanol and dried in vacuo at 30 °C. 2.15 g (36.7%) of a white crystalline powder were obtained. Mp. 109–110°C.

^{1}H-NMR (400 MHz, D_2O): 2.52 (dd, 1H, *J* = 16.0, 8.1 Hz, CH_2, H-malate), 2.71 (dd, 1H, *J* = 16.0, 4.2 Hz, CH_2, H-malate), 3.81 (dd, 1H, *J* = 12.9, 3.5 Hz, H5′A), 3.92-4.00 (m, H5′B), 4.24-4.31 (m, 1H, H3′ and 1H, CHOH, H-malate), 4.39 (q, 1H, *J* = 3.4 Hz, H4′), 4.43 (t, 1H, *J* = 4.7 Hz, H2′), 6.17 (d, 1H, *J* = 4.5 Hz, H1′), 8.20 (dd, 1H, *J* = 8.0, 6.3 Hz, H5), 8.90 (d, 1H, *J* = 7.6 Hz, H4), 9.19 (d, 1H, *J* = 6.3 Hz, H6), 9.51 (s, 1H, H2). Impurities: < 1 mol% nicotinamide; 0.9 mol% Et_3N salt. Solvents: 3.7 mol% methanol.
^{13}C-NMR (100 MHz, D_2O): 40.1 (CH_2, H-malate), 60.2 (C5′), 68.5 (CHOH, H-malate), 69.7 (C3′), 77.4 (C2′), 87.7 (C4′), 99.9 (C1′), 128.4 (C5), 133.9 (C3), 140.4 (C2), 142.6 (C6), 145.7 (C4), 165.8 ($CONH_2$), 176.3 (COO, H-malate), 179.1 (COO, H-malate).

Example 16b: Deacetylation using HBr in glacial acetic acid, neutralization using tributylamine

Deacylation of nicotinamide-2,3,5-tri-O-acetyl-D-ribofuranoside triflate: 2.00 g (3.8 mmol) of the triflate were dissolved in 8 mL of methanol while stirring. The solution was cooled down to 0–5°C. After addition of HBr 33% in glacial acetic acid (1.33 mL, 7.6 mmol, 2 eq), the green-yellowish solution was stirred at RT. After two days no educt could be determined in the solution by thin-layer chromatography.

Conversion to nicotinamide-β-D-ribofuranoside L-hydrogen malate after neutralization with tributylamine: 1.3 mL (5.45 mmol, 1.43 eq) of tributylamine were added to the above solution. After addition of 0.6 mL of water, any precipitated material was completely dissolved. 0.51 g (3.8 mmol, 1 eq) of L-malic acid were added to the brown-yellowish solution. After addition of further 0.9 mL (3.8 mmol, 1 eq) of tributylamine, the deacetylated product started crystallizing. It was filtered off, washed with methanol and dried in vacuo at 30 °C. 0.48 g (33 %) of nicotinamide-β-D-ribofuranoside L-hydrogen malate were obtained. Mp. 115–116°C.

^{1}H-NMR (400 MHz, D_2O): 2.55 (dd, 1H, *J* = 16.0, 8.1 Hz, CH_2, H-malate), 2.74 (dd, 1H, *J* = 16.0, 4.3 Hz, CH_2, H-malate), 3.83 (dd, 1H, *J* = 12.9, 3.5 Hz, H5′A), 3.98 (dd, 1H, *J* = 12.9, 2.9 Hz, H5′B), 4.28 (m, 1H, H3′), 4.30 (m, 1H, CHOH, H-malate), 4.39–4.42 (m, 1H, H4′), 4.44 (t, 1H, *J* = 4.8 Hz, H2′), 6.18 (d, 1H, *J* = 4.5 Hz, H1′), 8.21 (dd, 1H, *J* = 8.1, 6.3 Hz, H5), 8.91 (d, 1H, *J* = 7.8 Hz, H4), 9.20 (d, 1H, *J* = 6.3 Hz, H6), 9.53 (s, 1H, H2). Impurities: < 1 mol% nicotinamide; 0.35 mol% Bu_3N salt: 0.85 (t, 9H), 1.29 (m, 6H), 1.59 (m, 6H), 3.05 (q, 6H); solvents: 2.3 mol% methanol: 3.27 (s, 3H).
^{13}C-NMR (100 MHz, D_2O): 40.0 (CH_2, H-malate), 60.2 (C5′), 68.5 (CHOH, H-malate), 69.8 (C3′), 77.4 (C2′), 87.7 (C4′), 99.9 (C1′), 128.4 (C5), 133.9 (C3), 140.4 (C2), 142.6 (C6), 145.6 (C4), 165.8 ($CONH_2$), 176.3 (COO, H-malate), 179.0 (COO, H-malate).

Example 16c: Deacetylation using hydrochloric acid and neutralization using triethylamine

Deacylation of nicotinamide-2,3,5-tri-*O*-acetyl-β-D-ribofuranoside triflate: 8.00 g (15.1 mmol) of nicotinamide-2,3,5-tri-*O*-acetyl-β-D-ribofuranoside triflate were dissolved in 32 mL of methanol while stirring. 4.6 mL (25.8 mmol, 1.7 eq) of a 5.6 M solution of hydrogen chloride in ethanol were added while cooling. The resulting colorless solution was stirred at RT overnight. Control by thin-layer chromatography revealed complete deacetylation and some nicotinamide impurities.
Conversion to nicotinamide-β-D-riboside L-hydrogen malate after neutralization with triethylamine: 3.0 mL (21.5 mmol, 1.4 eq) of triethylamine were added dropwise to the above solution. 2.03 g (15.1 mmol, 1 eq) of L-malic acid were added, followed by additional 1.55 mL (11.1 mmol, 0.74 eq) of triethylamine. The product soon started precipitating. The suspension was stirred for a few h and then stored for 16 h in a refrigerator. After filtration, the obtained solid was washed with methanol and ethanol and dried in vacuo at 30 °C. 2.54 g (43.3%) of a white crystalline powder were obtained. Mp. 112–113 °C.

^{1}H-NMR (400 MHz, D_2O): Analogous to **Example 16a**. Impurities: < 1 mol% nicotinamide; 0.5 mol% Et_3N salt. Solvents: 3.7 mol% methanol.
^{13}C-NMR (100 MHz, D_2O): Analogous to **Example 16a**.

Example 16d: Deacetylation using triethylamine
Deacylation of nicotinamide-D-riboside-2,3,5-triacetate triflate: 3.00 g (5.66 mmol) of the triflate were dissolved in 18 mL of methanol while stirring. 0.8 mL of triethylamine (5.7 mmol, 1 eq) were added to the solution cooled down to 0 °C. After stirring for 4 days at 0–5°C, thin-layer control showed complete conversion.
Conversion to nicotinamide-β-D-riboside L-hydrogen malate: The brown-orange solution obtained in the step above was warmed to RT. Subsequently, 0.80 g (5.9 mmol, 1.04 eq) of L-malic acid were added. Product started precipitating after a few minutes. The product suspension was cooled down to 0 °C and stirred. After storage in a refrigerator for 12 h, the suspension was filtered, the obtained solid washed with methanol and ethanol and dried in vacuo at 30 °C. 0.95 g (43 %) of a yellow-beige crystalline powder were obtained. Mp. 111–112°C

^{1}H-NMR (400 MHz, D_2O): Analogous to **Example 16a**. Impurities: < 1 mol% nicotinamide; 1 mol% Et_3N salt. Solvents: 11 mol% methanol.
^{13}C-NMR (100 MHz, D_2O): Analogous to **Example 16a**.

Example 17: Preparation of nicotinamide-2,3,5-tri-*O*-acetyl-β-D-ribofuranoside iodide
6.00 g (49 mmol, 1.04 eq) of nicotinamide and 14.9 g (47 mmol) of β-D-ribofuranose-1,2,3,5-tetraacetate were suspended upon stirring at RT in 190 mL of acetonitrile which has been dried over 3 Å molecular sieves. The suspension was warmed to 35 °C while most of the solids dissolved. 7.0 mL (49 mmol, 1.04 eq) of trimethylsilyl iodide were added within 20 minutes and the yellow suspension was stirred for a further two hours at 35 °C. Subsequently, the internal temperature was kept at 40 °C and 45 °C for one hour each. The solvent was removed in vacuo at 35 °C. The formed foam was dissolved in 100 mL of dichloromethane and 1.2 g of activated charcoal were added. The suspension was filtered, and the filtrate was concentrated in vacuo. Nicotinamide-2,3,5-tri-*O*-acetyl-β-D-ribofuranoside iodide was isolated as a deep yellow foam (22 g, 93%).

^{1}H-NMR (400 MHz, D_2O): 2.03, 2.06, 2.10 (3x s, 3 x 3H, $COCH_3$), 4.43-4.50 (m, 2H, H5′), 4.81-4.86 (m, 1H, H4′), 5.40 (t, 1H, *J* = 5.4 Hz, H3′), 5.49-5.55 (m, 1H, H2′), 6.55 (d, 1H, *J* = 3.9 Hz, H1′), 8.24 (dd,

1H, *J* = 8.1, 6.3 Hz, H5), 9.16 (d, 1H, H4), 9.18 (d, 1H, H6), 9.38 (s, 1H, H2). Impurities: 24 mol% ribose tetraacetate, 16 mol% alpha-anomer, 3 mol% nicotinamide.
^{13}C-NMR (100 MHz, D_2O): 20.0, 20.1, 20.4 (3 x $COCH_3$), 62.6 (C5′), 69.3 (C3′), 76.1 (C2′), 82.5 (C4′), 97.2 (C1′), 128.8 (C5), 134.1 (C3), 140.4 (C2), 143.1 (C6), 146.2 (C4), 165.1 ($CONH_2$), 172.0, 172.1, 173.0 (3 x CO).

Example 18: Deacetylation of nicotinamide-β-D-riboside-2,3,5-triacetate iodide
Deacetylation using sulfuric acid and neutralization using triethylamine
Preparation of a diluted sulfuric acid in methanol: 10 mL of methanol were cooled down to 0 °C. 1.20 mL (21.6 mmol) of a 96% sulfuric acid were added while stirring. The methanolic sulfuric acid was used in the deacetylation below.
Deacylation of nicotinamide-2,3,5-tri-*O*-acetyl-β-D-ribofuranoside iodide: 11.0 g (21.6 mmol) of nicotinamide-2,3,5-tri-*O*-acetyl-β-D-ribofuranoside iodide were dissolved in 33 mL of methanol while stirring. The above prepared methanolic sulfuric acid was added. The resulting orange-brown solution was stirred at room temperature for 24 h. Control by thin-layer chromatography revealed complete deacylation and some impurities. 3.5 mL (25.1 mmol, 1.16 eq) of triethylamine were added. The solution was divided into two halves.
Conversion to nicotinamide-β-D-riboside L-hydrogen tartrate after neutralization with triethylamine: To one half of the above solution 1.65 g (11 mmol, 1.02 eq) of L-tartaric acid were added, followed by further 1.6 mL (11.5 mmol, 1.06 eq) of triethylamine. Product started precipitating almost immediately. The product suspension was stirred one h at RT, two h in an ice-bath and stored for 12 h in a refrigerator. After filtration, the obtained solid was washed with methanol and dried in vacuo at 30 °C. Nicotinamide-β-D-ribofuranoside L-hydrogen tartrate was isolated as an almost white crystalline powder (2.10 g, 48%). Mp. 125.5–126°C.

^{1}H-NMR (400 MHz, D_2O): Analogous to **Example 15a**. Impurities: 1 mol% nicotinamide; 3.8 mol% Et_3N salt. Solvents: 18.2 mol% methanol.
^{13}C-NMR (100 MHz, D_2O): Analogous to **Example 15a**.

Conversion to nicotinamide-β-D-riboside L-hydrogen malate after neutralization with triethylamine: 1.45 g of L-malic acid (10.8 mmol, 1 eq) were added to the other half of the above solution, followed by further 1.1 mL (7.9 mmol, 0.73 eq) of triethylamine. The solution was seeded and the product started precipitating a few min later. The product suspension was stirred one h at RT, two h in an ice-bath, then stored for 12 h in a refrigerator. After filtration, the obtained solid was washed with methanol and ethanol and dried in vacuo at 30 °C. Nicotinamide-β-D-ribofuranoside L-hydrogen malate was isolated as an almost white crystalline solid (1.37 g, 32.7%). Mp. 114–115°C.

^{1}H-NMR (400 MHz, D_2O): Analogous to **Example 16a**. Impurities: 0.5 mol% nicotinamide; 0.5 mol% Et_3N salt. Solvents: 2.4 mol% methanol, 0.4 mol% ethanol.
^{13}C-NMR (100 MHz, D_2O): Analogous to **Example 16a**.

Section S3: Solubility of NR^+ crystalline salts

Table S3: Solubilities (in mL solvent per g of solute) of the stereoisomeric crystalline NR^+ salts and the halogenides in methanol and in aqueous solutions.

**NR^+ salts**	**Solubility in MeOH**	**Solubility in MeOH / H_2O 4:1**	**Solubility in Water**
D-Hydrogen malate	1300	220	3.3
L-Hydrogen malate	500	50	1.05
DL-Hydrogen malate	750	120	2.3
D-Hydrogen tartrate	400	40	4.2
L-Hydrogen tartrate	3800	410	5.3
DL-Hydrogen tartrate	2600	300	3.8
Bromide	50	6	0.5
Chloride	40	5	0.55

Section S4: Single Crystal Growth and Structural Determination of Nicotinamide riboside derivatives by X-Ray Diffraction

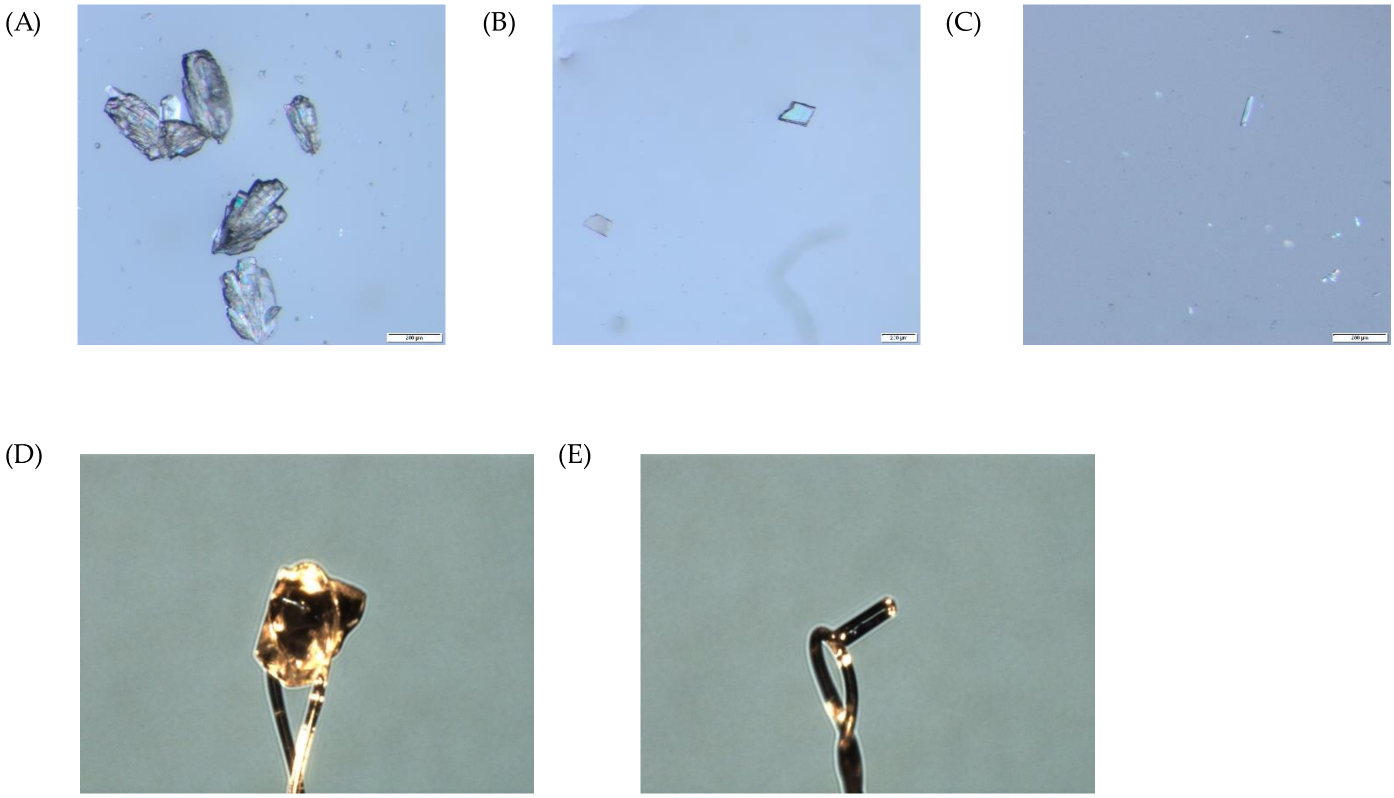

Figure S1: Photos of the grown crystals of **11a** (anhydrate of **11**) (A), **11b** (monohydrate of **11**) (B), **12** (C), **13** (D) and **14** (E).

Table S4: Summary of Crystal Data and Structure Refinement of the Salts **11a**, **11b**, **12**, **13**, and **124**.

	**11a** (anhydrate)	**11b** (hydrate)	**12**	**13**	**14**
Empirical formula	$C_{15}H_{20}N_2O_{11}$	$C_{15}H_{22}N_2O_{12}$	$C_{15}H_{20}N_2O_{11}$	$C_{15}H_{20}N_2O_{10}$	$C_{15}H_{20}N_2O_{10}$
Formula weight	404.33	422.34	404.33	388.33	388.33
Crystal system	Monoclinic	Orthorhombic	Monoclinic	Monoclinic	Orthorhombic
Space group	$P2_1$	$P2_12_12_1$	$P2_1$	$P2_1$	$P2_12_12_1$
a [Å]	7.52820(10)	7.71190(10)	7.64990(12)	7.66800(10)	7.35565(8)
b [Å]	7.70690(10)	14.56510(10)	13.8774(2)	8.21630(10)	7.82204(8)
c [Å]	14.33700(10)	15.1856(2)	7.80215(13)	13.4615(2)	28.1002(2)
α [°]	90	90	90	90	90
β [°]	96.8240(10)	90	96.2221(15)	96.4630(10)	90
γ [°]	90	90	90	90	90
Volume [$Å^3$]	825.927(16)	1705.72(3)	823.40(2)	842.72(2)	1616.78(3)
Z	2	4	2	2	4
Density (calculated) [Mg/m^3]	1.626	1.645	1.631	1.530	1.595
Temperature [K]	160.00(10)	160.00(10)	159.99(10)	160.00(10)	160.00(10)
Wavelength [Å]	1.54184	1.54184	0.71073	0.71073	1.54184
Absorption coefficient [mm^{-1}]	1.223	1.256	0.141	0.130	1.175
F(000)	424	888	424	408	816
Crystal size [mm^3]	0.111 x 0.055 x 0.022	0.127 x 0.025 x 0.02	0.226 x 0.073 x 0.065	0.32 x 0.28 x 0.204	0.215 x 0.051 x 0.045
Crystal description	colorless plate	colorless needle	colorless block	colorless block	colorless needle
Theta range for data collection [°]	3.104 to 79.576	4.206 to 79.535	2.626 to 37.874	2.673 to 37.797	5.872 to 79.555
Index ranges	-6<=h<=9<br>-9<=k<=9<br>-18<=l<=18	-9<=h<=9<br>-18<=k<=18<br>-18<=l<=15	-13<=h<=11<br>-23<=k<=23<br>-13<=l<=13	-12<=h<=13<br>-14<=k<=14<br>-22<=l<=23	-9<=h<=9<br>-8<=k<=9<br>-35<=l<=28
Reflections collected	14426	18041	34397	34851	17769
Independent reflections	3442 [R(int) = 0.0369]	3683 [R(int) = 0.0273]	8442 [R(int) = 0.0249]	8567 [R(int) = 0.0305]	3505 [R(int) = 0.0324]
Reflections observed	3367	3589	7909	8252	3423
Criterion for observation	I > 2 σ(I)	I > 2 σ (I)	I > 2 σ (I)	I > 2 σ (I)	I > 2 σ (I)
Completeness to theta	97.6 % to 67.684°	100.0 % to 67.684°	100.0 % to 25.242°	99.9 % to 25.242°	99.9 % to 67.684°
Absorption correction	Gaussian	Gaussian	Gaussian	Gaussian	Gaussian
Max. and min. transmission	1.000 and 0.854	1.000 and 0.793	1.000 and 0.807	1.000 and 0.420	1.000 and 0.632
Data / restraints / parameters	3442 / 1 / 260	3683 / 0 / 275	8442 / 1 / 259	8567 / 1 / 249	3505 / 0 / 250
Goodness-of-fit on F^2	1.070	1.054	1.074	1.039	1.084
Final R indices [I > 2 σ (I)]	R1 = 0.0307, wR2 = 0.0803	R1 = 0.0244, wR2 = 0.0638	R1 = 0.0322, wR2 = 0.0870	R1 = 0.0297, wR2 = 0.0828	R1 = 0.0294, wR2 = 0.0789
R indices (all data)	R1 = 0.0318, wR2 = 0.0841	R1 = 0.0253, wR2 = 0.0641	R1 = 0.0350, wR2 = 0.0887	R1 = 0.0309, wR2 = 0.0835	R1 = 0.0304, wR2 = 0.0800
Absolute structure parameter	0.04(7)	0.02(5)	0.01(14)	0.03(14)	0.01(6)
Extinction coefficient	0.0030(8)	0.0011(2)	n/a	n/a	0.0014(3)
Largest diff. peak and hole [$e.Å^{-3}$]	0.280 and -0.195	0.202 and -0.175	0.445 and -0.222	0.497 and -0.230	0.426 and -0.210

Discussion of the hydrogen bonding network of **11a**

There are several direct hydrogen bonds and other chain motifs in the structure. The nitrogen of the amide group forms hydrogen bonds with the oxygen atoms of hydroxyl groups on a neighboring hydrogen tartrate molecule (N2-H2B O12 (+x, +y, 1+z): 2.779(3) Å, N2-H2B O14 ((+x, +y, 1+z): 3.020(3) Å). O2, O3, and O5 of hydroxyl groups on the riboside form individual hydrogen bonds with the carbonyl of the hydrogen tartrate, and two neighboring riboside units, respectively (O2-H2 O13: 2.616(2) Å), O3-H O5 (+x, 1+y, +z): 2.718(3) Å), (O5-H5 O2 (-x, -0.5+y, 1-z): 2.772(3) Å). The donor O3 is also the start of a seven membered chain ending with the acceptor atom. The donor O5 is also the start of a six membered chain which ends at the acceptor O2. Two oxygen atoms of hydroxyl groups on the same hydrogen tartrate form hydrogen bonds with the carbonyl amide of a different neighboring riboside (O14-H14 O1 (-x, -0.5+y, 1-z): 2.805(3) Å, O15-H15 O1 (1-x, -0.5 + y, 1-z): 2.823(3) Å. An additional hydroxyl oxygen of the riboside forms a hydrogen bond with an oxygen of a neighboring hydrogen tartrate (O17-H17 O12 (-x, -1+y, +z): 2.473(3) Å). From this donor atom, O17, a seven membered chain motif propagates to the acceptor O12.

Discussion of the hydrogen bonding network of **11b**

There are various hydrogen bonding motifs in the structure. The hydroxyl groups of the riboside form hydrogen bonds with oxygen atoms of the neighboring hydrogen tartrate and O2 forms a hydrogen bond with the oxygen of the water molecule (O3-H3 O13 (1 + x, + y, + z): 2.8723(18) Å, O5-H5 O2 (1 + x, + y, + z): 2.7760(17) Å, O2-H2 O6: 2.6299(18) Å). From the donor atom O5, there begins a seven membered chain ending with the acceptor O2. The oxygen of the water molecule forms additional hydrogen bonds with the carbonyl oxygen of the hydrogen tartrate molecule and a hydroxyl group of a neighboring hydrogen tartrate molecule (O6-H6A O13: 2.7584(19) Å, O6-H6B O17 (1 + x, + y, + z): 2.8330(19) Å). The hydroxy groups of hydrogen tartrate molecule at O14, O15, and O17 form hydrogen bonds with the oxygen of a neighboring riboside sugar, the amide group, and neighboring hydrogen tartrate molecule, respectively (O14-H14 O5 (1 – x, -0.5 + y, 0.5 - z): 2.7797(19) Å, O15-H15 O1 (-1.5 + x, 0.5 – y, 1-z): 2.9135(19) Å, O17-H17 O12 (-1 + x, + y, + z) 2.4916(17) Å). At O17 a seven membered chain begins and extends to the acceptor atom O12. Further there is an intramolecular hydrogen bond from O15 of the hydrogen tartrate hydroxyl group to the carbonyl oxygen (O15-H15 O16: 2.7083(18) Å).

Discussion of the hydrogen bonding network of **12**

There are various discrete hydrogen bonds present. The amide group forms hydrogen bonds with a carbonyl and a hydroxyl oxygen of two neighboring hydrogen tartrate molecules (N2-H2A O16 (-1 - x, -0.5 + y, -z): 2.9950(13) Å, N2-H2B O15 (-x, -0.5 + y, -z): 2.9925(13) Å). There are hydrogen bonds between different hydroxyl group of the riboside with two oxygens of the hydrogen tartrate molecule in the same asymmetric unit (O2-H2 O12: 2.7951(12) Å, O2-H2 O14: 2.8782(13) Å). Other hydroxyl oxygens of the riboside form hydrogen bonds with a carbonyl of a neighboring hydrogen tartrate and with a hydroxyl oxygen of a neighboring riboside (O3-H3 O13 (-x, 0.5+y, -z): 2.6638(12) Å, O5-H5 O2 (1 + x, + y, + z): 2.7162(13) Å). At O5 of the riboside a seven membered chain motif begins, ending at the acceptor atom O2. The hydrogen tartrate hydroxyl groups form hydrogen bonds from to the riboside in the same asymmetric unit, a neighboring riboside and a neighboring hydrogen tartrate molecule (O14-H14 O1: 2.8011(13) Å, O15-H15 O5 (-1 + x, + y, 1 + z): 2.6544(13) Å, and O17-H17 O12 (-1 + x, + y, + z): 2.5755(11) Å). A fourteen membered ring is formed from the riboside by the proton of O2 to O14 of the hydrogen tartrate, and the proton of O14 forms a hydrogen bond with O1, in the same asymmetric unit. Additionally, at the donor atom, O17, there begins a seven membered chain motif ending with the acceptor O12.

Discussion of the hydrogen bonding network of **13**

The nitrogen of the amide group forms hydrogen bonds with a deprotonated oxygen and a carbonyl of two neighboring hydrogen malate molecules (N2-H2A O14 (-1 + x, +y, 1 + z): 3.0865(12) Å, N2-H2B O13 (+ x, + y,

1+z): 2.8994(11) Å). Two hydroxyl oxygens, O2, and O3, of the riboside form hydrogen bonds with oxygen atoms of neighboring hydrogen malate (O2-H2 O15 (-1 +x, +y, +z): 2.74313(11) Å, O3-H3 O12: 2.6532(11) Å). A third hydroxyl oxygen of the riboside forms a hydrogen bond with the oxygen of a neighboring amide (O5-H5 O1 (1 +x, +y, +z): 2.7685(11) Å). This hydrogen bond beginning with the donor O5 propagates through a chain of eleven atoms and ends with the acceptor atom, O1. Another hydroxyl oxygen of the riboside forms a hydrogen bond to an oxygen of a neighboring riboside (O2-H2 O3 (1+ x, + y, + z): 2.694(2) Å). A hydroxyl oxygen of the hydrogen malate O16 forms a hydrogen bond with a hydroxyl and carbonyl oxygen of neighboring ribosides and O12 forms a hydrogen bond with a neighboring hydrogen malate oxygen (O16-H16 O12 (1 + x, + y, + z): 2.5449(11) Å). This hydrogen bond from O16 propagates through a chain of seven atoms ending with the acceptor atom O12.

Discussion of the hydrogen bonding network of **14**

There are several hydrogen bonding motifs. The nitrogen of the amide group forms a hydrogen bond with hydroxyl oxygens of two neighboring hydrogen malate molecules (N2-H2A O15 (0.5 - x, -y, -0.5 + z): 3.005(2) Å, N2-H2B O14 (0.5 -x, 1 - y, - 0.5 + z): 3.024(2) Å). Hydroxyl oxygen atoms of the riboside O3 and O5 form hydrogen bonds with the hydrogen malate in the same asymmetric unit and to a neighboring hydrogen malate, respectively (O3-H3 O13: 2.677(2) Å, O5-H5 O13 (1 - x, 0.5 +y, 1.5 - z): 2.722(2) Å). Another hydroxyl oxygen of the riboside forms a hydrogen bond to an oxygen of a neighboring riboside (O2-H2 O3 (1 - x, -0.5 +y, 1.5 -z): 2.694(2) Å). The hydrogen bond from O2 propagates through a five-atom chain, ending again with the acceptor atom O3. Hydroxyl oxygen atoms of the hydrogen malate, O14 and O16 form hydrogen bonds, the first with a hydroxyl and carbonyl oxygen of neighboring ribosides, and the second with a neighboring hydrogen malate oxygen (O14-H14 O5 (-x, -0.5 + y, 1.5 - z): 2.887(2) Å, O14-H14 O1 (-x, 0.5 + y, 1.5 - z): 2.918(2) Å, O16-H16 O12 (+ x, -1 + y,+ z): 2.580(2) Å. The hydrogen bond beginning from O16-H16 propagates through 7 atoms before repeating with O16-H16 of the neighboring hydrogen malate (+x, -1 + y, + z).

(A)

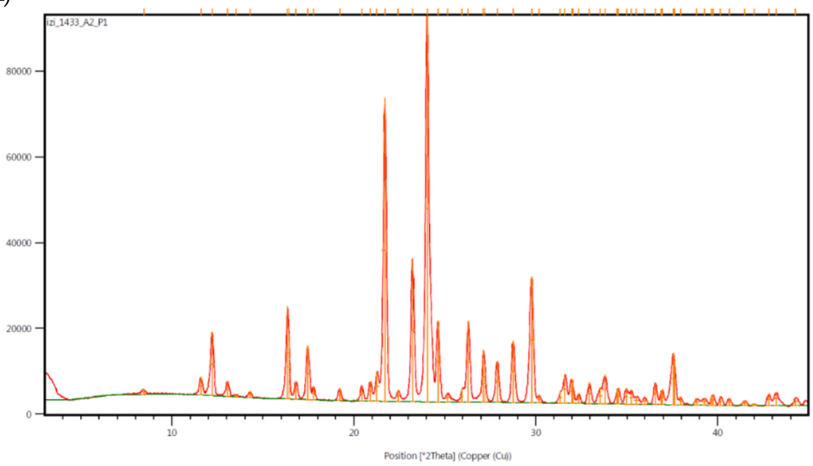

(B)

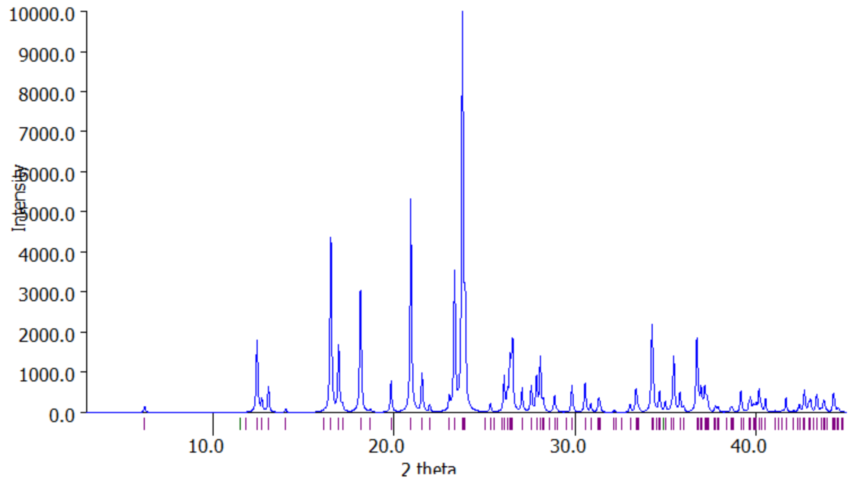

(C)

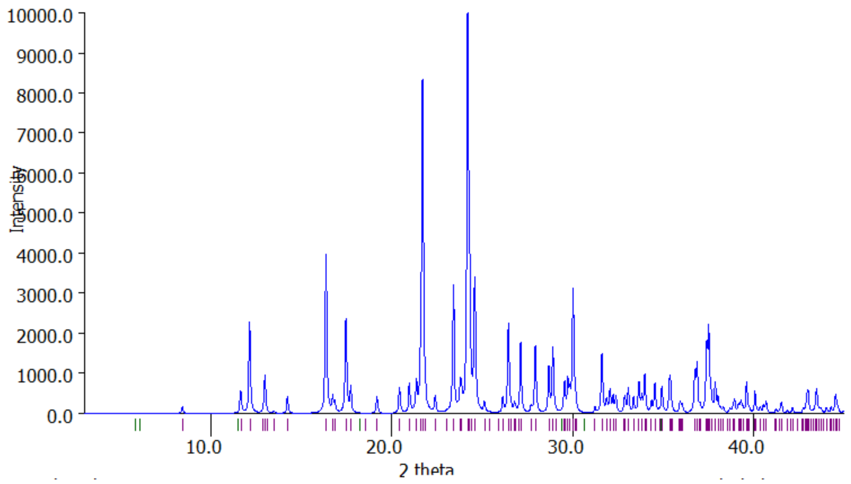

Figure S2: X-ray powder pattern of **11** (A). X-ray powder pattern of **11a** (anhydrous) simulated from single crystal data (B). X-ray powder pattern of **11b** (monohydrate) simulated from single crystal data (C).

(A)

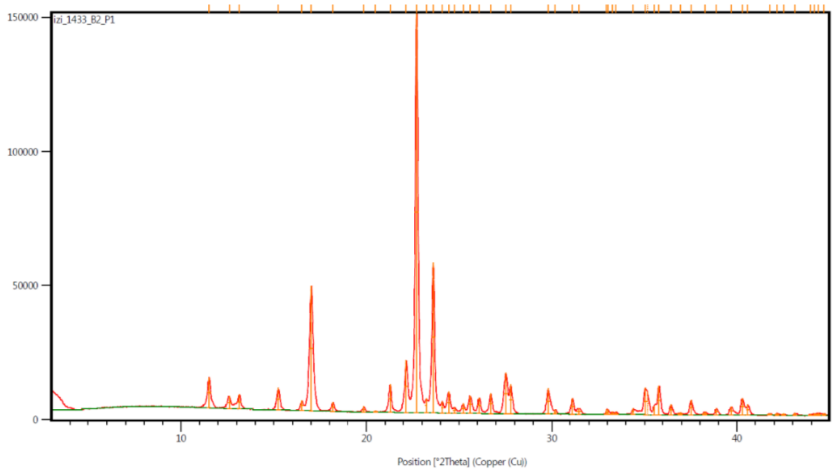

(B)

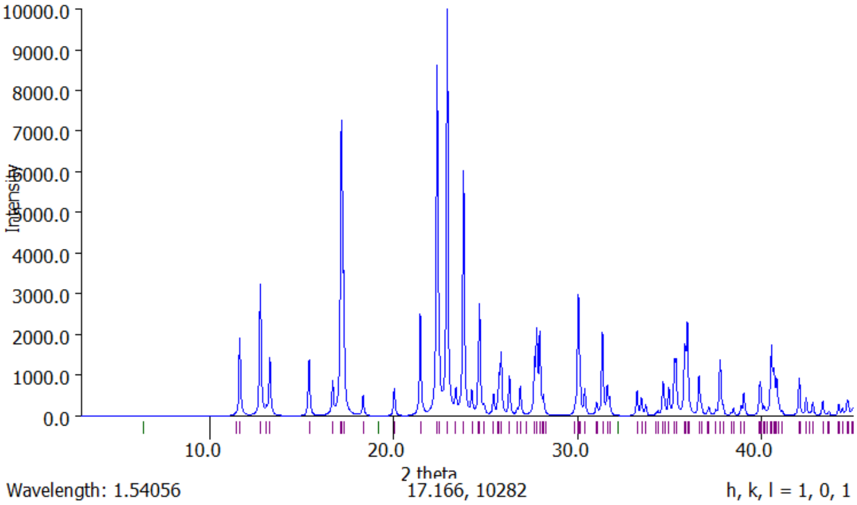

Figure S3: X-ray powder pattern of **12** (A). X-ray powder pattern of **12** simulated from single crystal data (B).

(A)

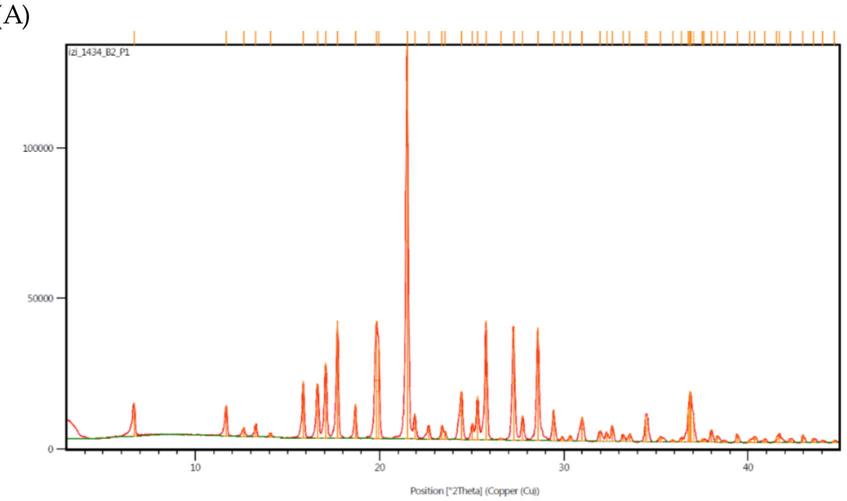

(B)

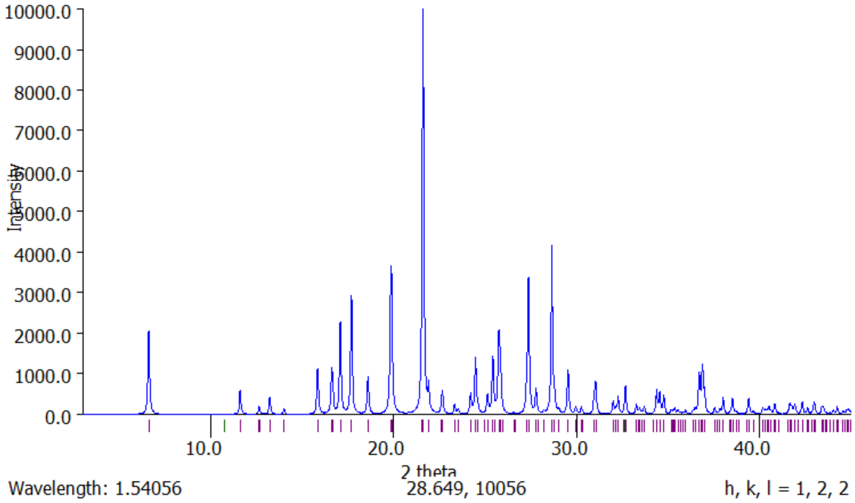

Figure S4: X-ray powder pattern of **13** (A). X-ray powder pattern of **13** simulated from single crystal data (B).

(A)

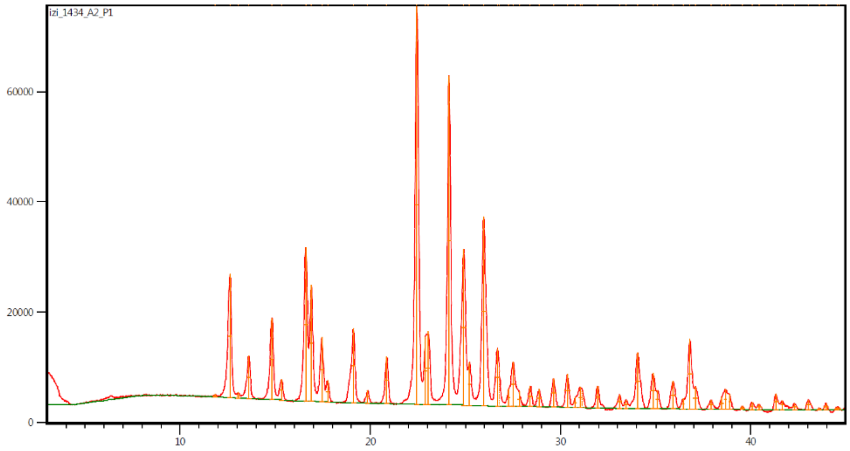

(B)

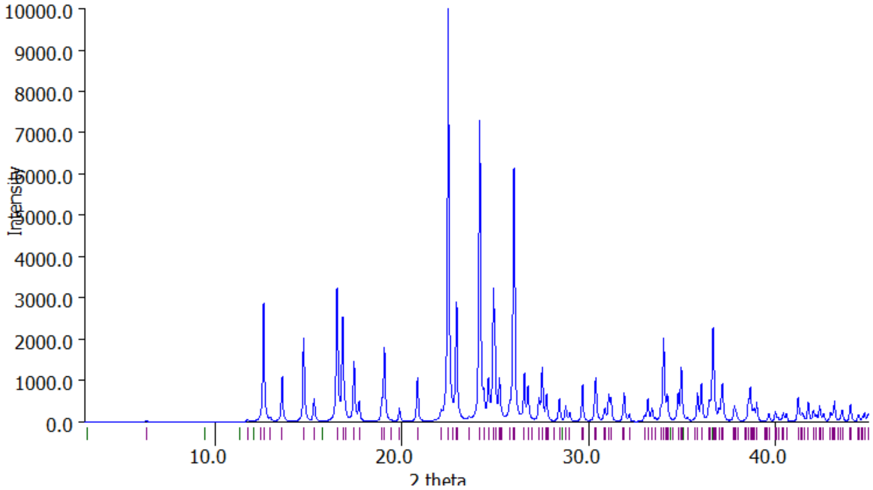

Figure S5: X-ray powder pattern of **14** (A). X-ray powder pattern of **14** simulated from single crystal data (B).

Section S5: Stability studies of NR⁺ salts

(A)

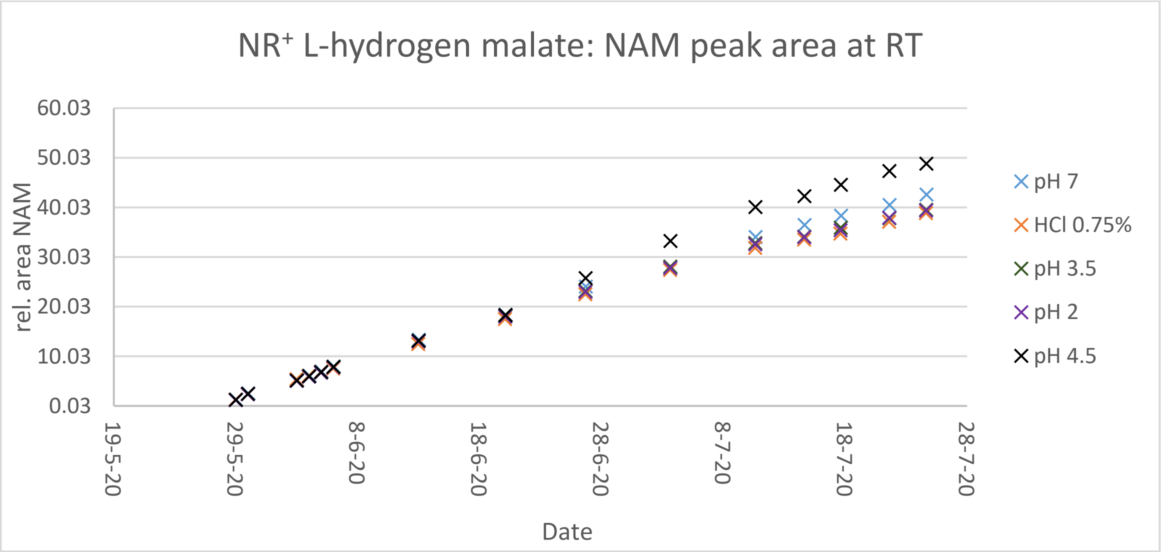

(B)

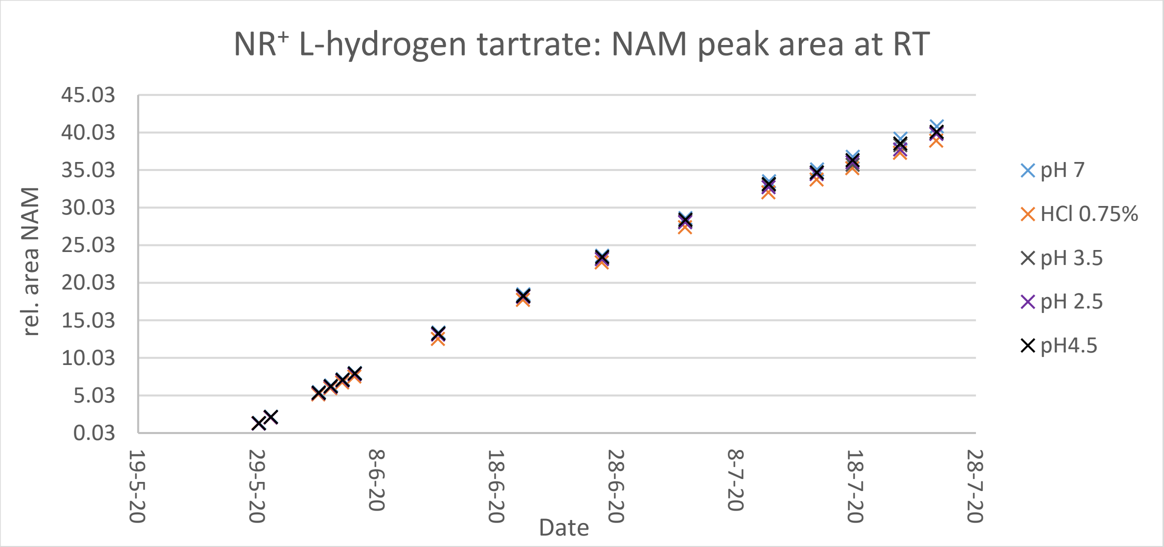
NR⁺ L-hydrogen tartrate: NAM peak area at RT
rel. area NAM
Date
pH 7
HCl 0.75%
pH 3.5
pH 2.5
pH4.5

(C)

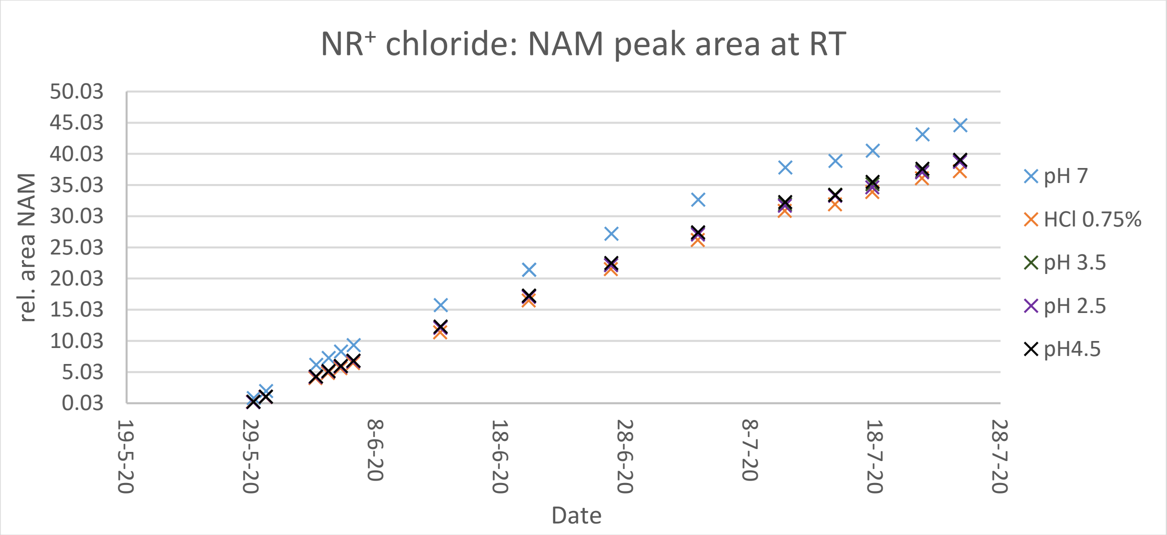
NR⁺ chloride: NAM peak area at RT
rel. area NAM
Date
pH 7
HCl 0.75%
pH 3.5
pH 2.5
pH4.5

(D)

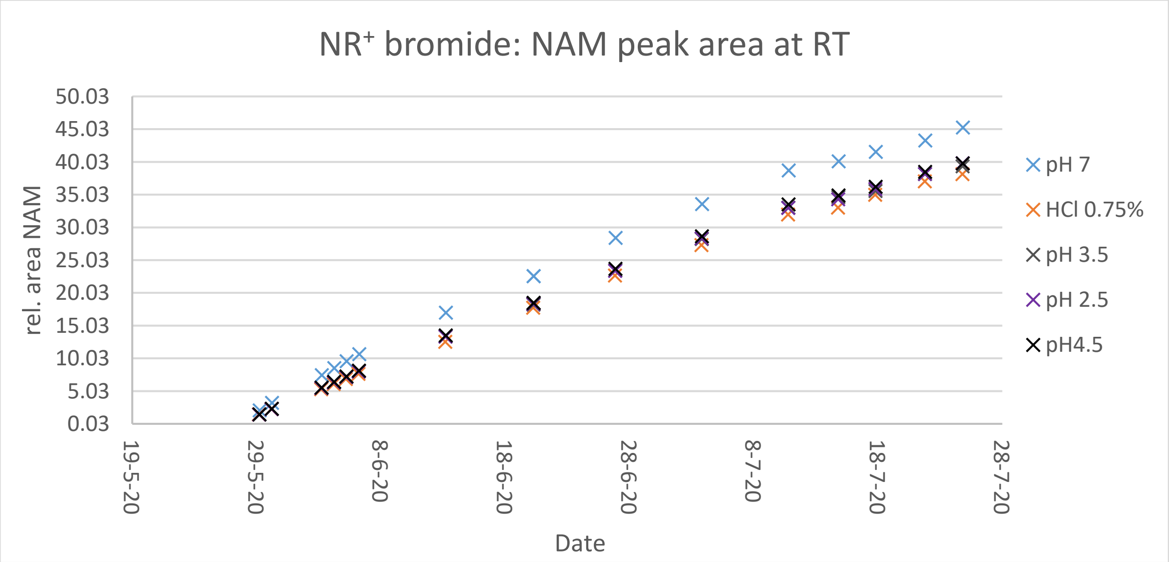
NR⁺ bromide: NAM peak area at RT
rel. area NAM
Date
pH 7
HCl 0.75%
pH 3.5
pH 2.5
pH4.5

(E)

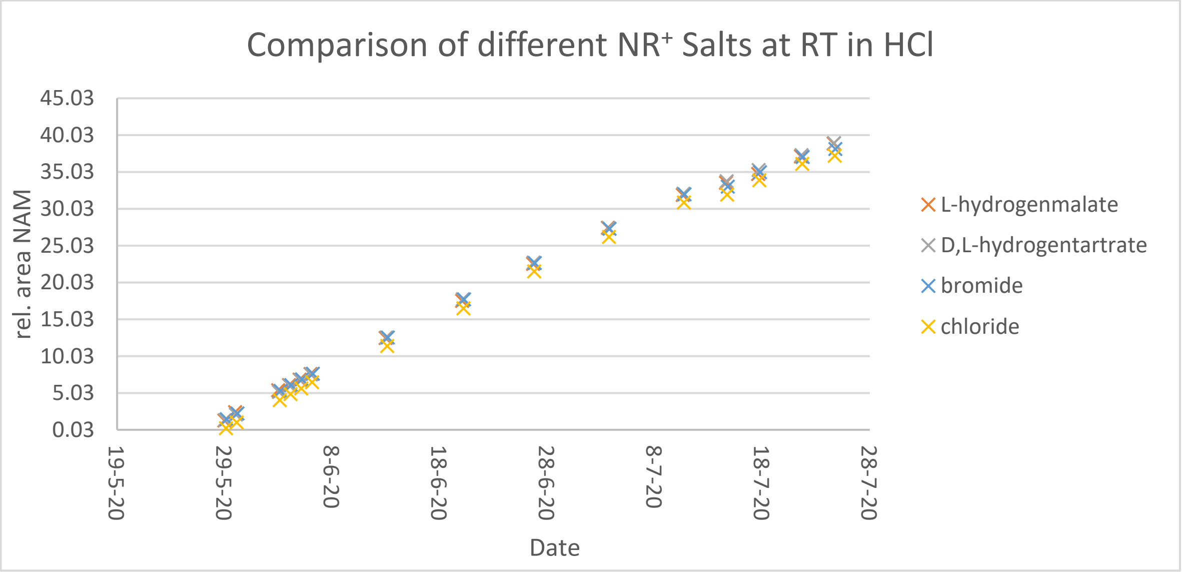

Figure S6: NAM peak area measurement over time at RT at various pH for NR^+ L-hydrogen malate (A), NR^+ D,L-hydrogen tartrate (B), NR^+ chloride (C), NR^+ bromide (D); Comparison of the different NR^+ Salts at RT in HCl (E).

(A)

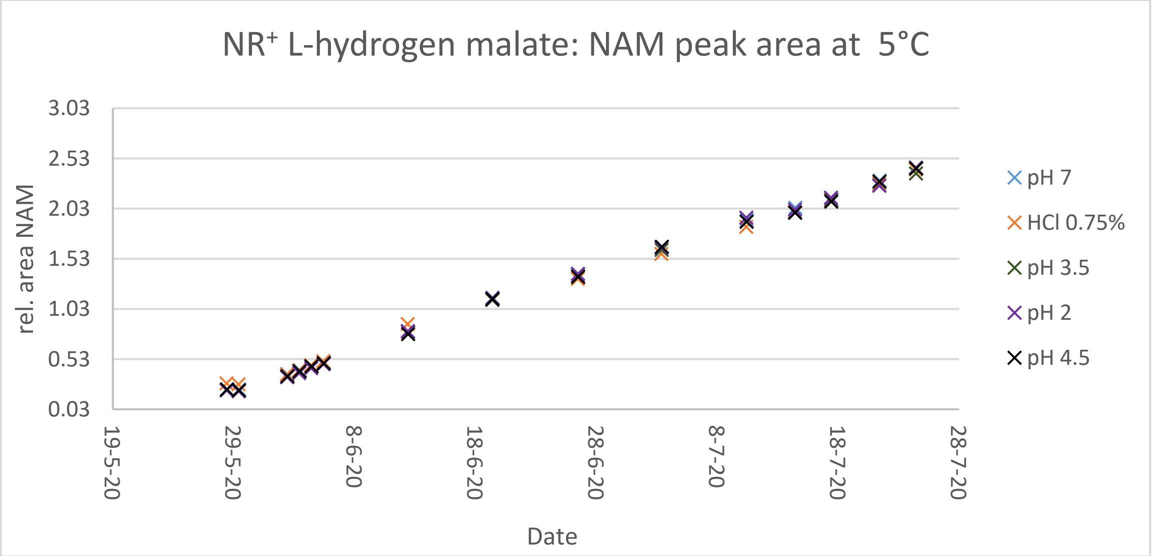

(B)

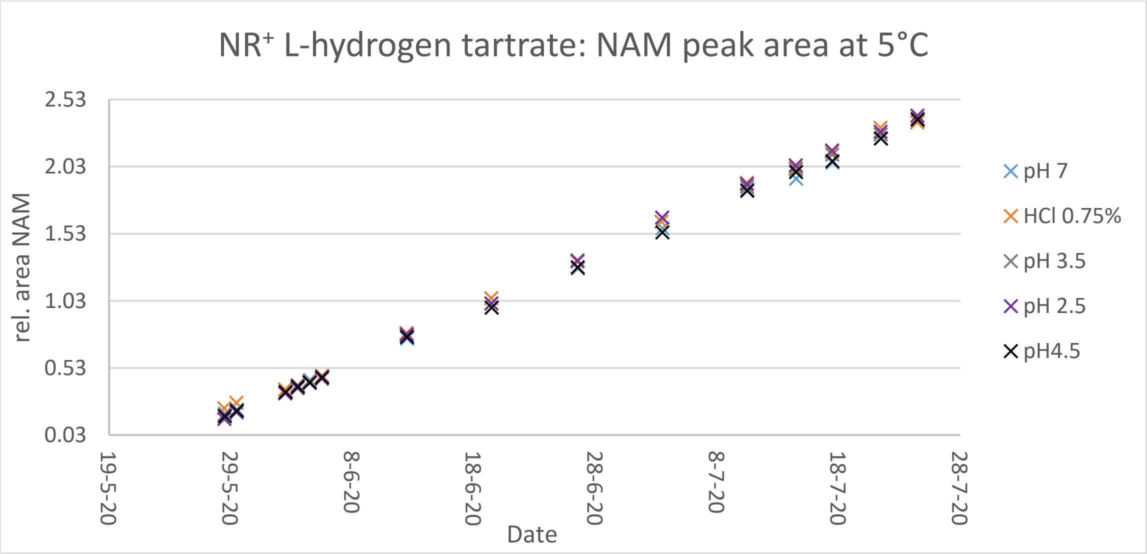
NR⁺ L-hydrogen tartrate: NAM peak area at 5°C
rel. area NAM
2.53
2.03
1.53
1.03
0.53
0.03
19-5-20
29-5-20
8-6-20
18-6-20
28-6-20
8-7-20
18-7-20
28-7-20
Date
pH 7
HCl 0.75%
pH 3.5
pH 2.5
pH4.5

(C)

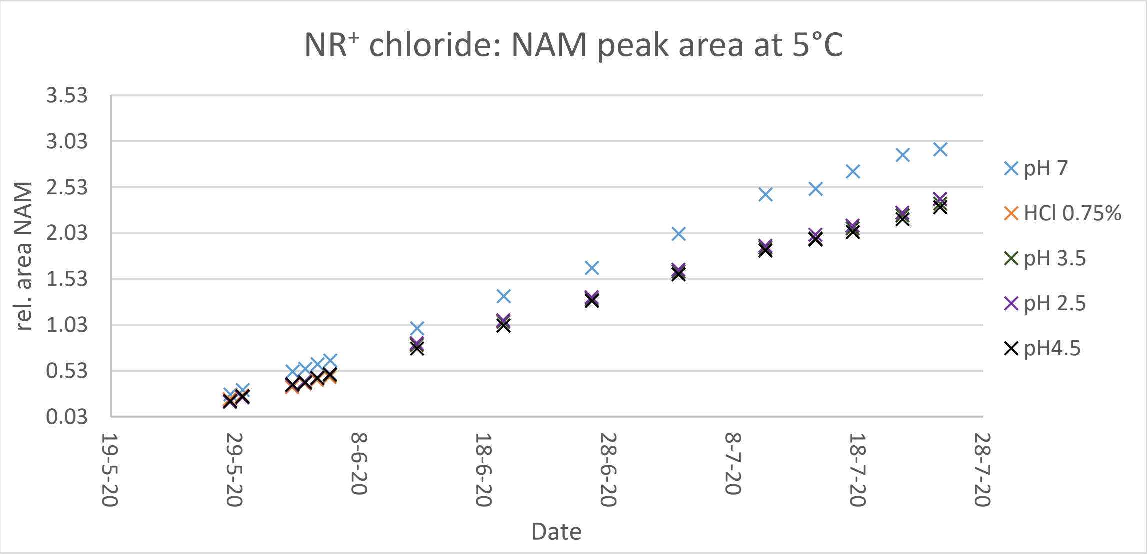
NR⁺ chloride: NAM peak area at 5°C
rel. area NAM
3.53
3.03
2.53
2.03
1.53
1.03
0.53
0.03
19-5-20
29-5-20
8-6-20
18-6-20
28-6-20
8-7-20
18-7-20
28-7-20
Date
pH 7
HCl 0.75%
pH 3.5
pH 2.5
pH4.5

(D)

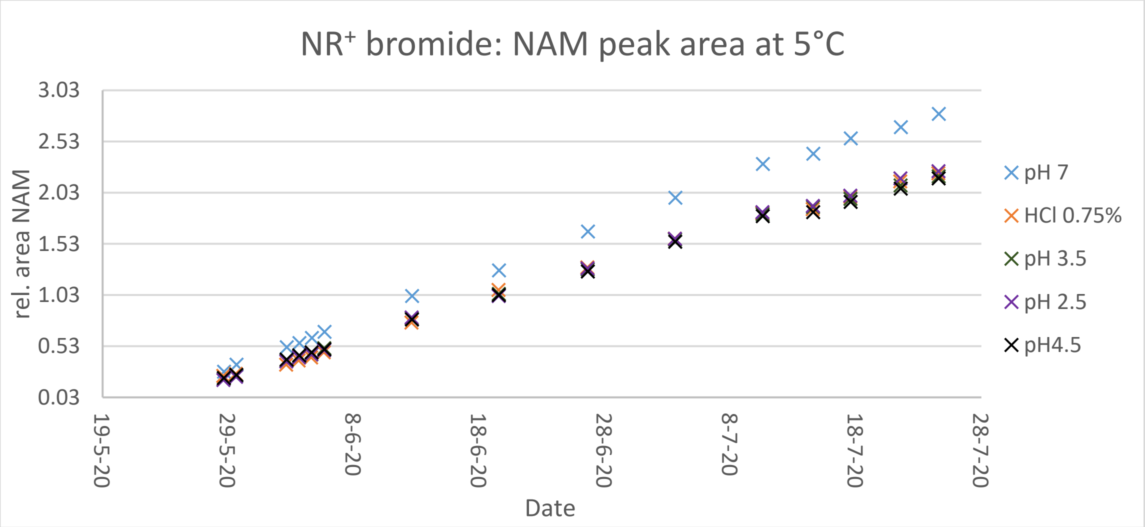

(E)

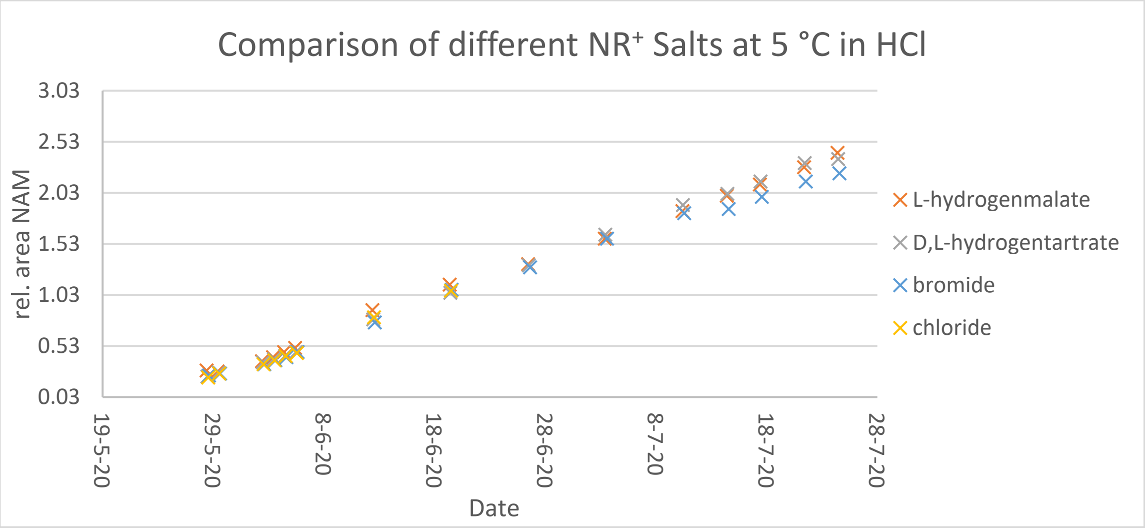

Figure S7: NAM peak area measurement over time at 5 °C at various pH for NR^+ L-hydrogen malate (A), NR^+ D,L-hydrogen tartrate (B), NR^+ chloride (D), NR^+ bromide (E); Comparison of the different NR^+ Salts at 5 °C in HCl (F).

(A)

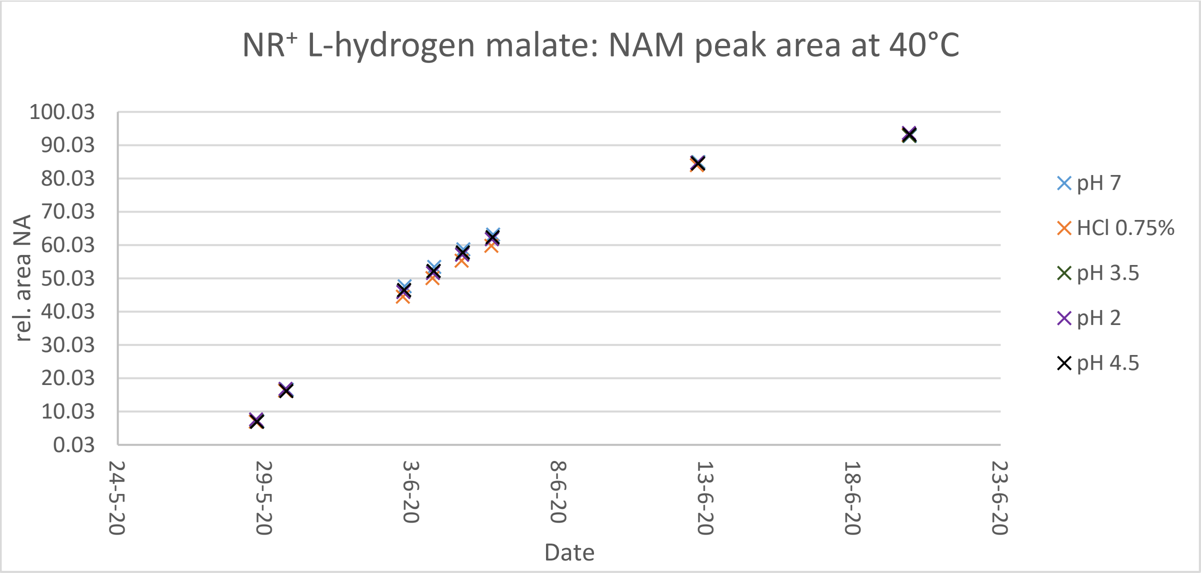

NR⁺ L-hydrogen malate: NAM peak area at 40°C
100.03
90.03
80.03
70.03
60.03
50.03
40.03
30.03
20.03
10.03
0.03
rel. area NA
24-5-20
29-5-20
3-6-20
8-6-20
13-6-20
18-6-20
23-6-20
Date
pH 7
HCl 0.75%
pH 3.5
pH 2
pH 4.5

(B)

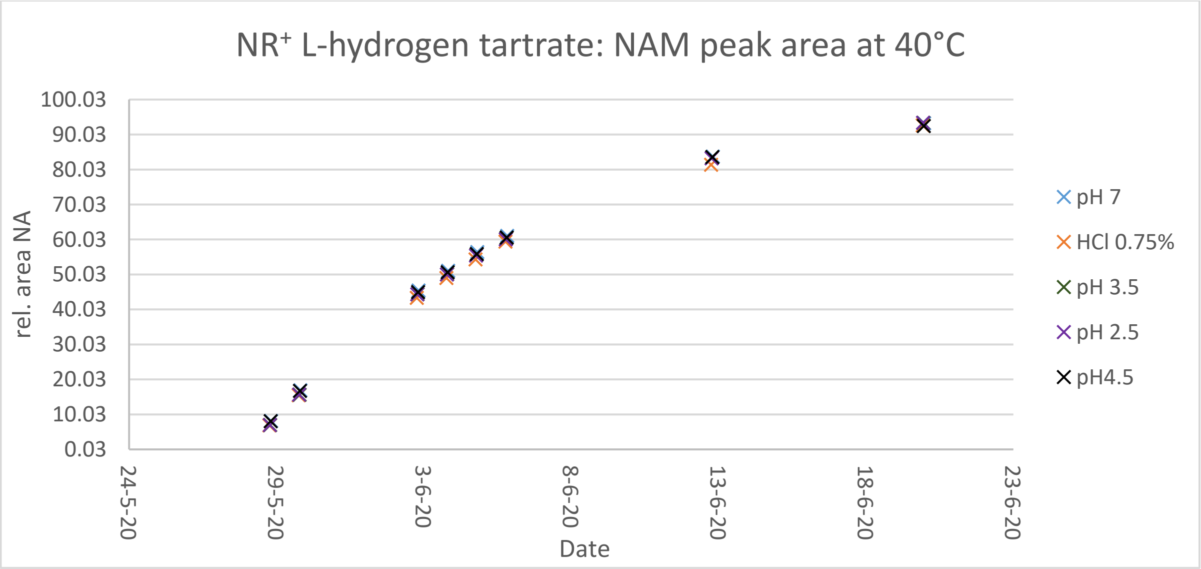

NR⁺ L-hydrogen tartrate: NAM peak area at 40°C
100.03
90.03
80.03
70.03
60.03
50.03
40.03
30.03
20.03
10.03
0.03
rel. area NA
24-5-20
29-5-20
3-6-20
8-6-20
13-6-20
18-6-20
23-6-20
Date
pH 7
HCl 0.75%
pH 3.5
pH 2.5
pH4.5

(C)

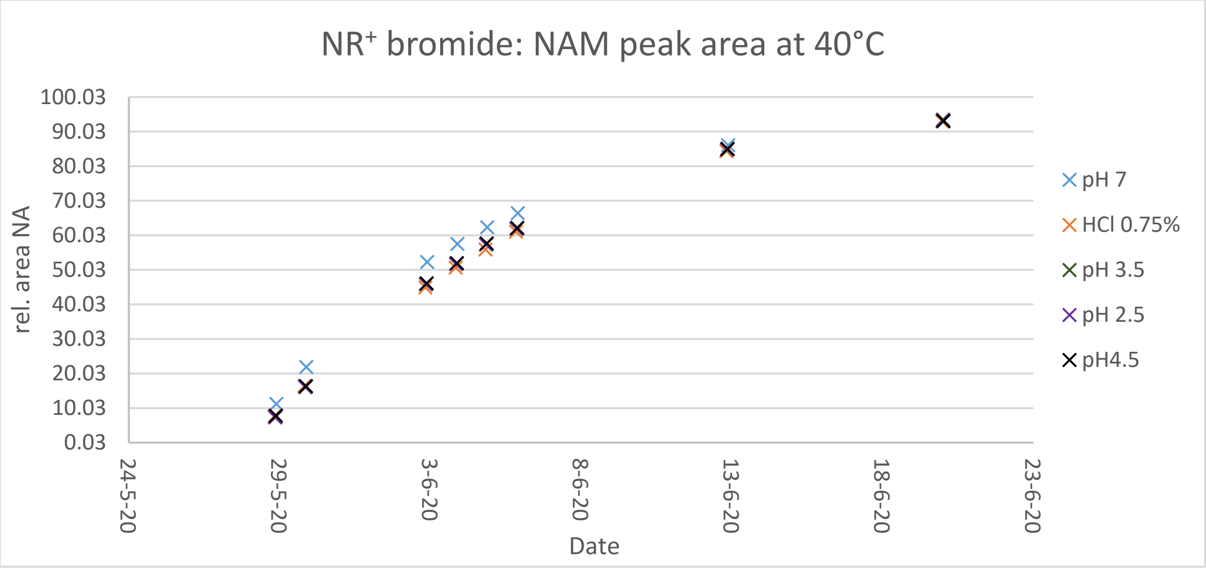
NR⁺ bromide: NAM peak area at 40°C
rel. area NA
Date
pH 7
HCl 0.75%
pH 3.5
pH 2.5
pH4.5

(D)

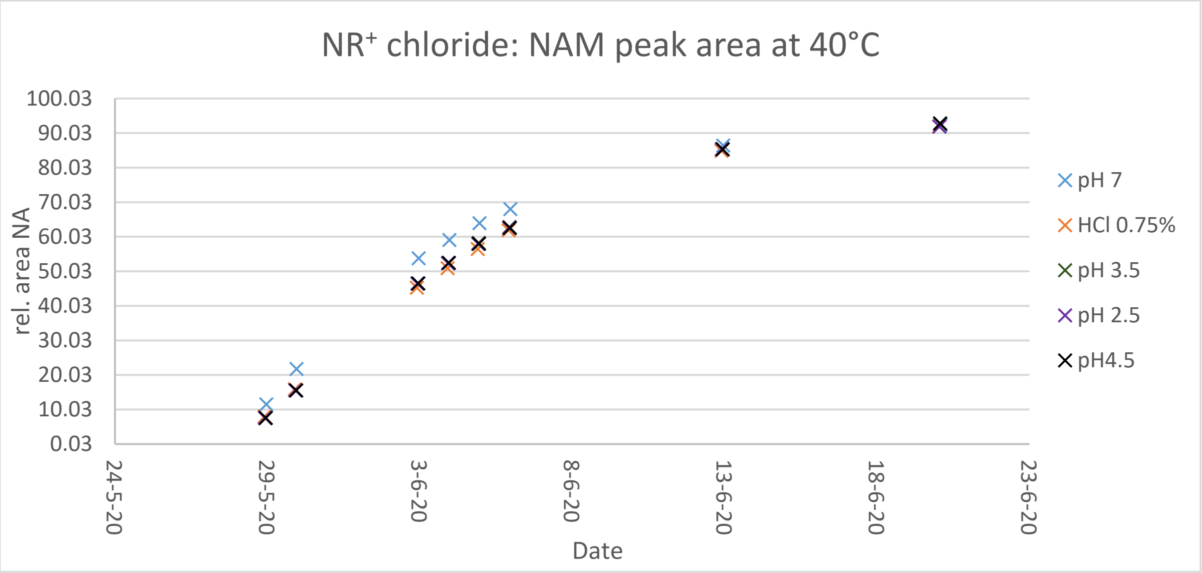
NR⁺ chloride: NAM peak area at 40°C
rel. area NA
Date
pH 7
HCl 0.75%
pH 3.5
pH 2.5
pH4.5

(E)

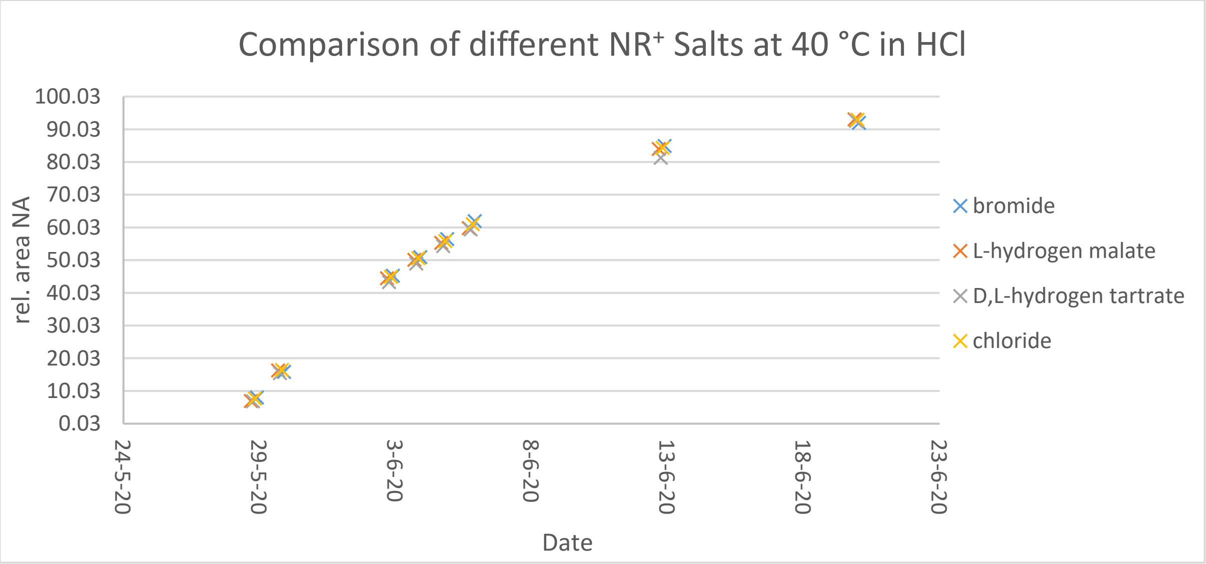
Comparison of different NR⁺ Salts at 40 °C in HCl
rel. area NA
Date
bromide
L-hydrogen malate
D,L-hydrogen tartrate
chloride

Figure S8: NAM peak area measurement over time at 40 °C at various pH for NR^+ L-hydrogen malate (A), NR^+ D,L-hydrogen tartrate (B), NR^+ chloride (D), NR^+ bromide (E); Comparison of the different NR^+ Salts at 40 °C in HCl (F).
