# Supplementary material for: New Crystalline Salts of Nicotinamide Riboside as Food Additives
Source: Molecules. 2021 May 6;26(9):2729. doi: 10.3390/molecules26092729 (PMC8125264; doi:10.3390/molecules26092729)

Example 1: NR L-ascorbate ^{1}H-NMR (D_2O, 400 MHz)

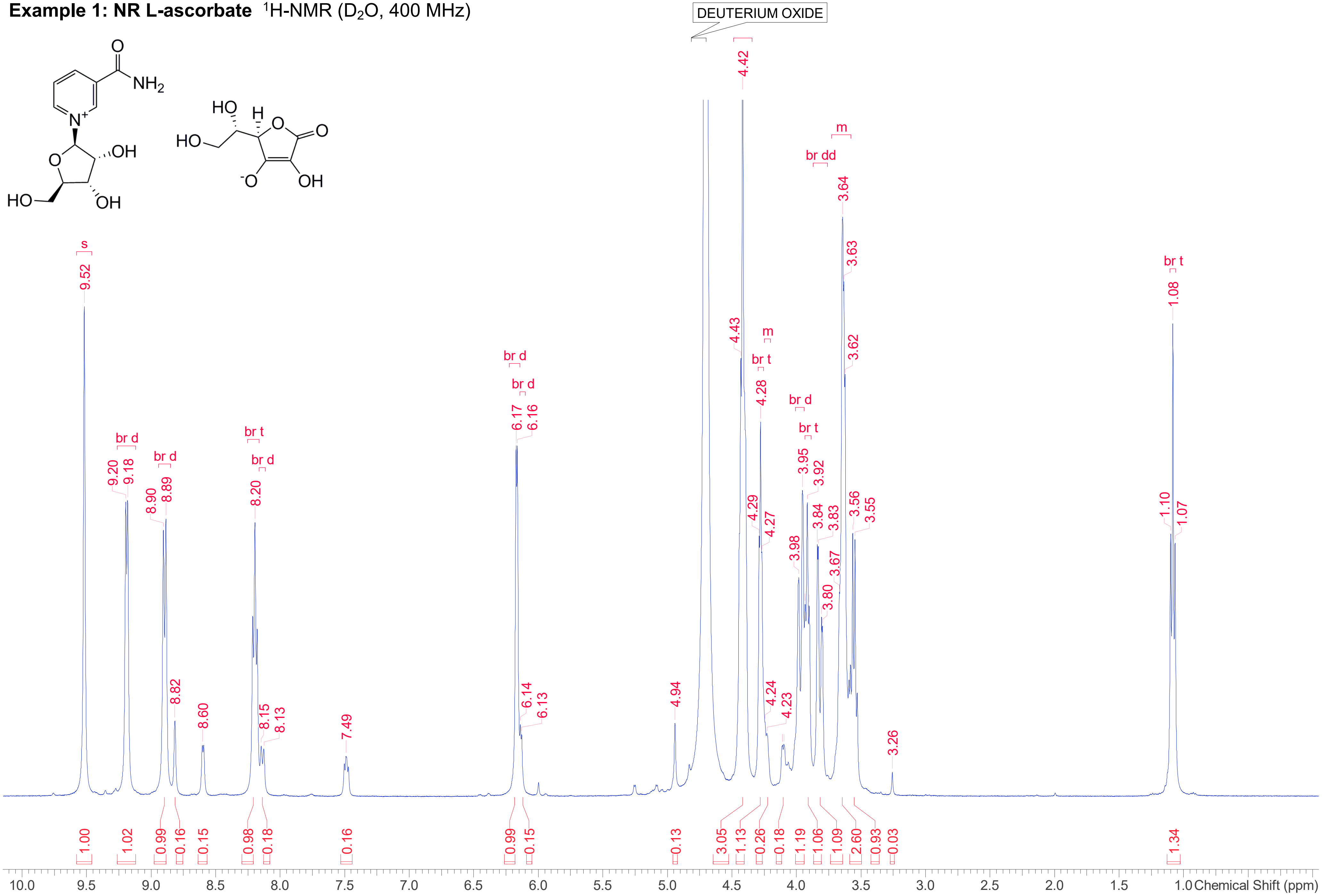

Example 1: NR L-ascorbate ^{13}C-NMR (D_2O, 100 MHz)

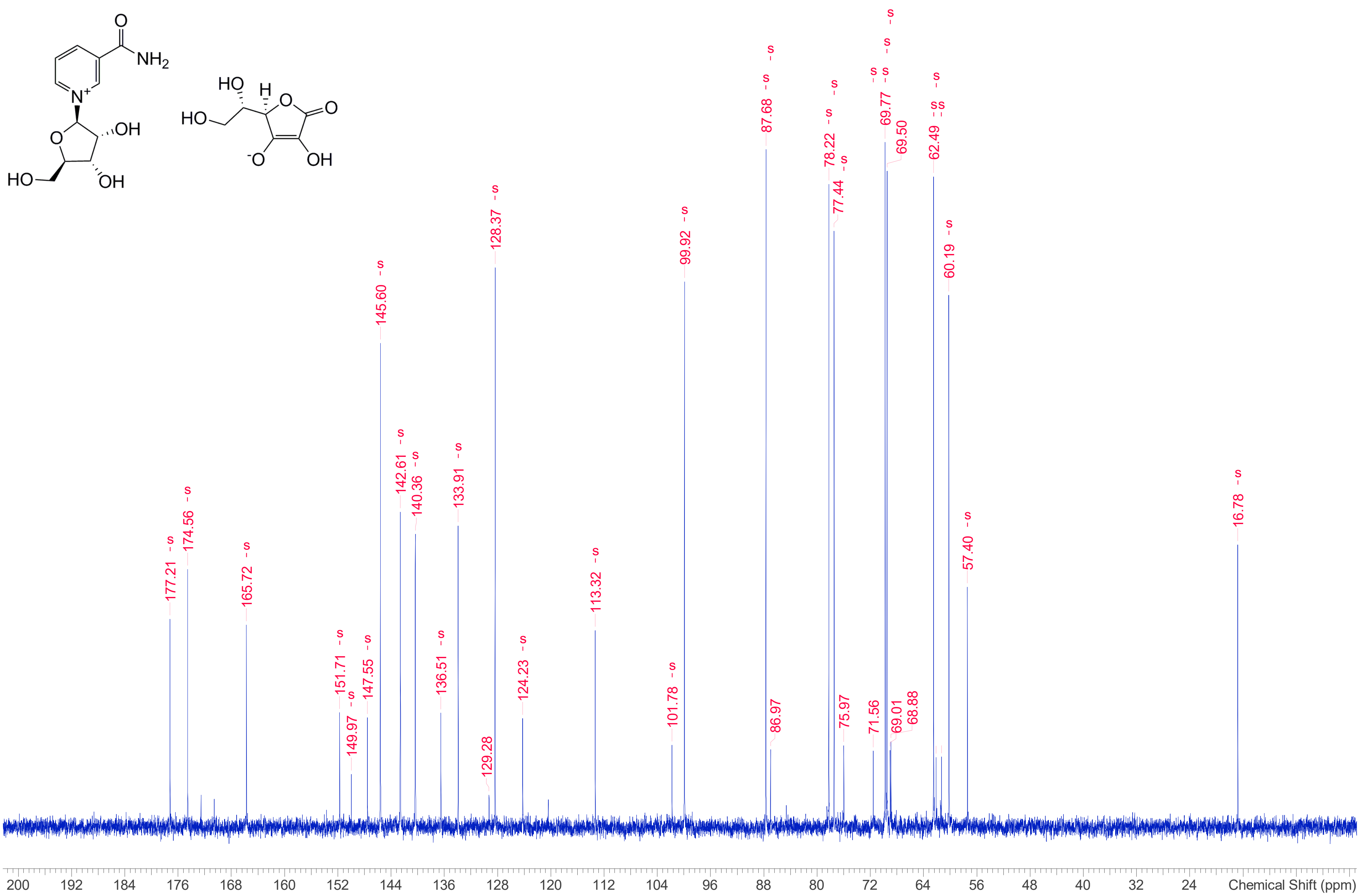

Example 2: NR citrate ^{1}H-NMR (D_2O, 400 MHz)

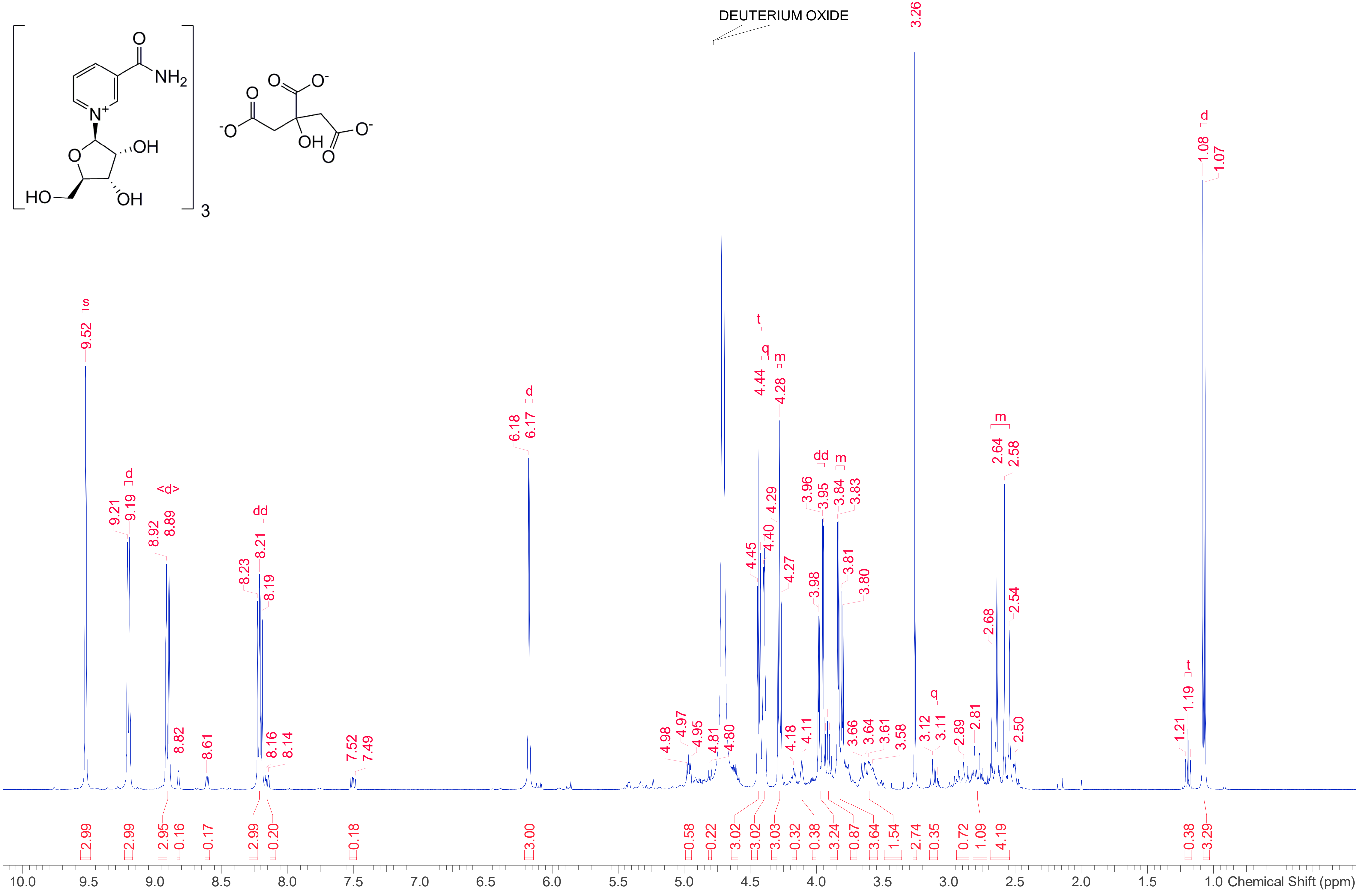

Example 2: NR citrate ^{13}C-NMR (D_2O, 100 MHz)

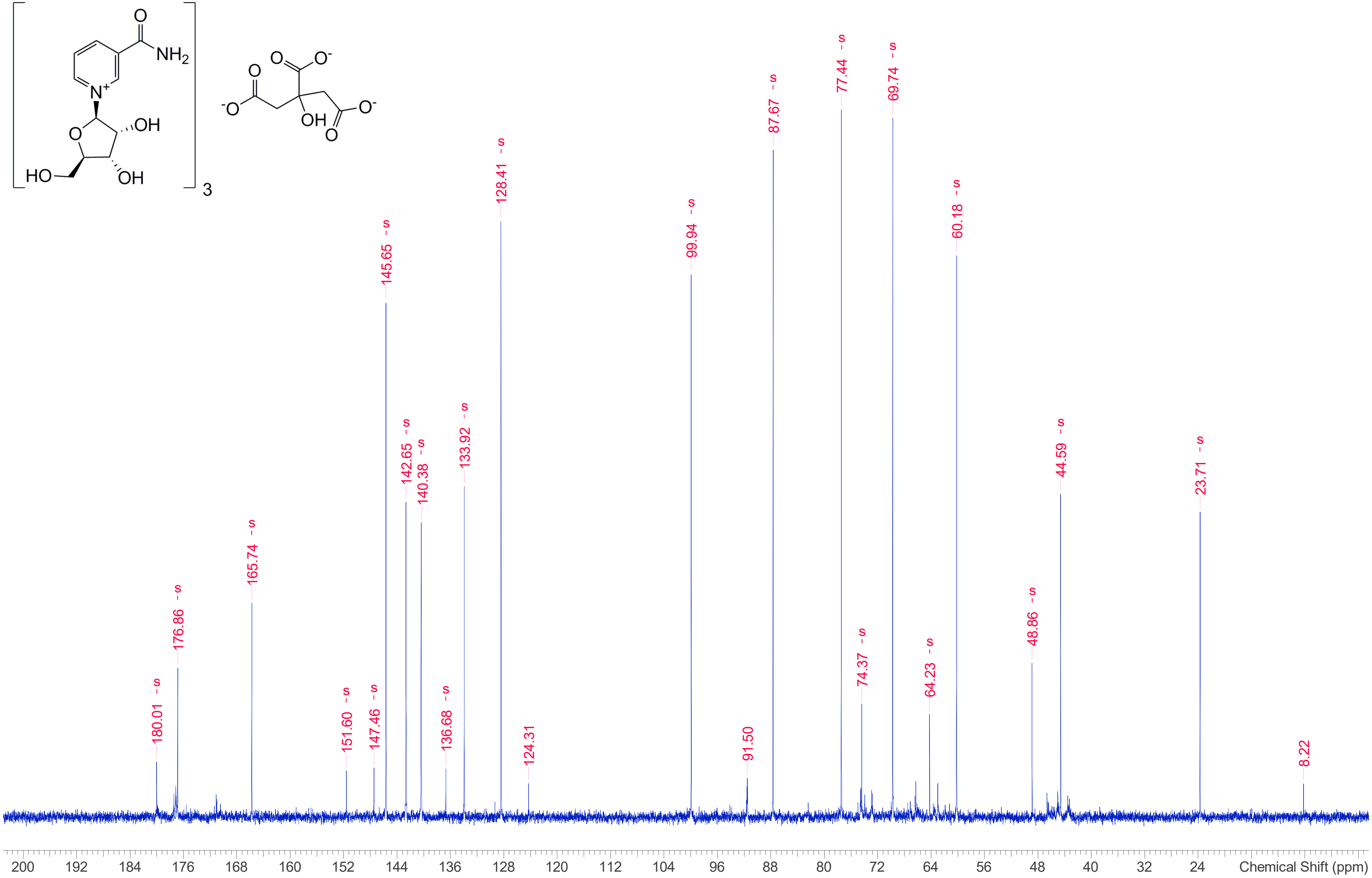

Example 3: NR bromide ^{1}H-NMR (D_2O, 400 MHz)

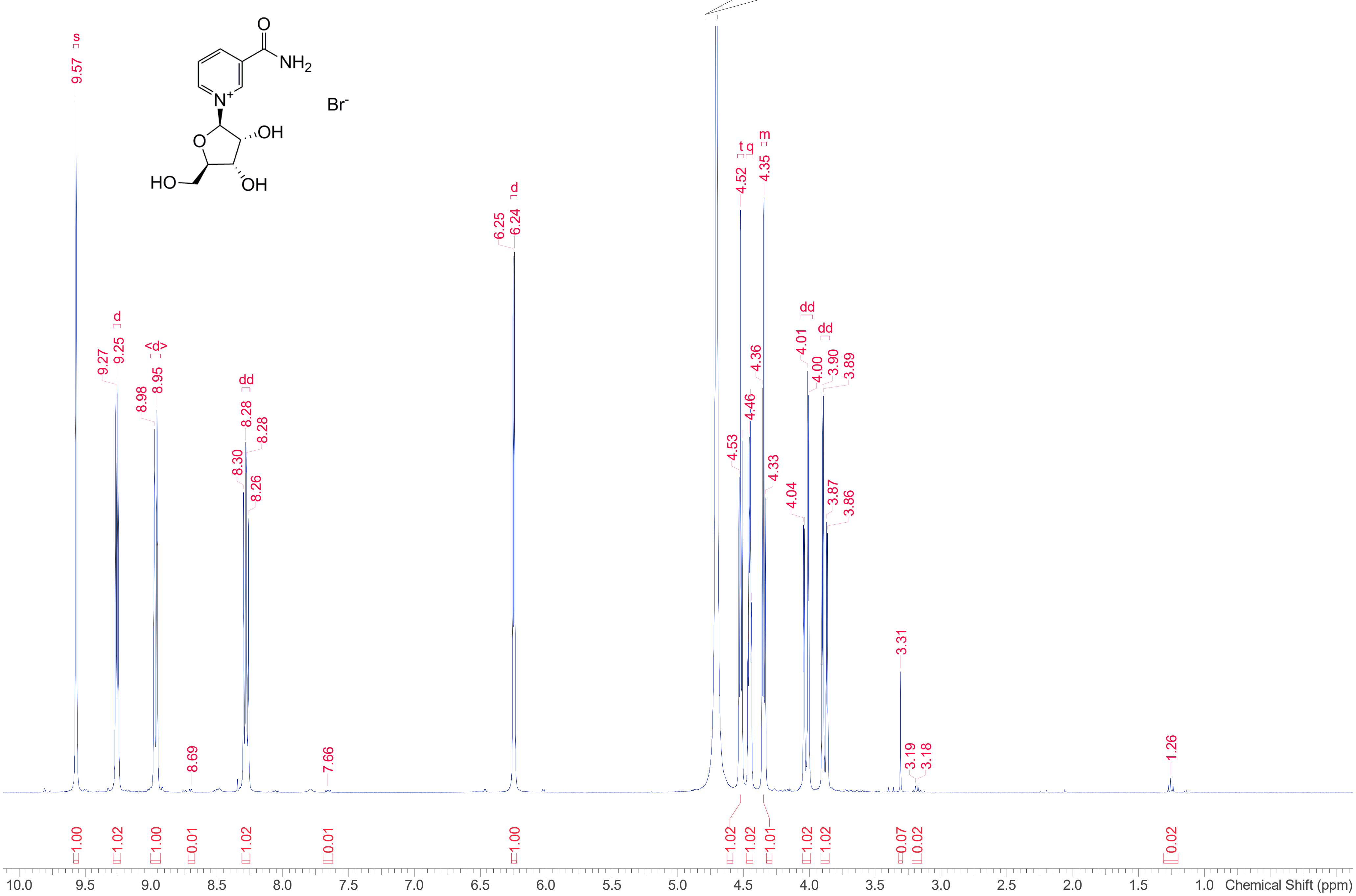

Example 3: NR bromide ^{13}C-NMR (D_2O, 100 MHz)

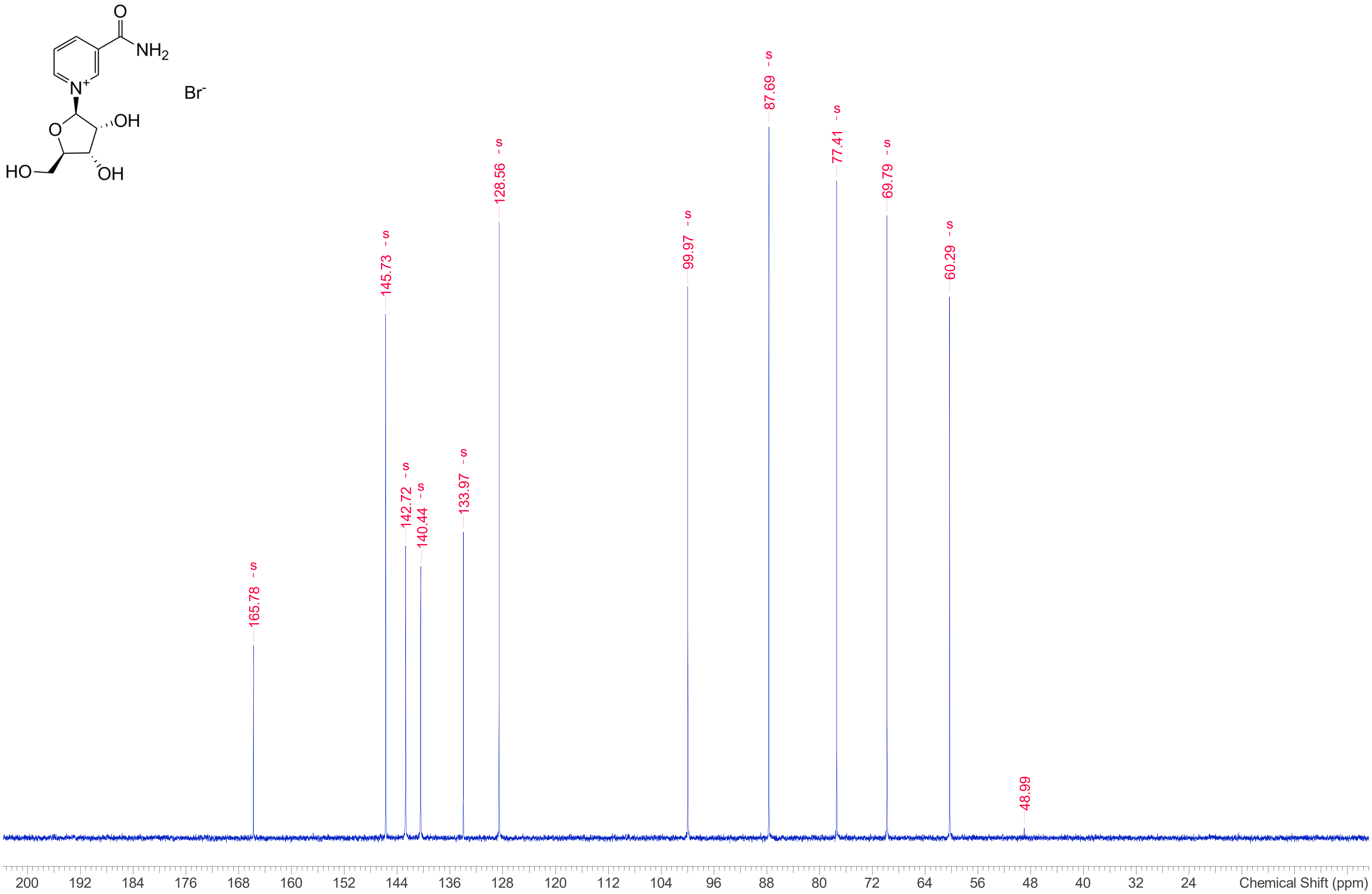

Example 4: NR D-glucuronate ^{1}H-NMR (D_2O, 400 MHz)

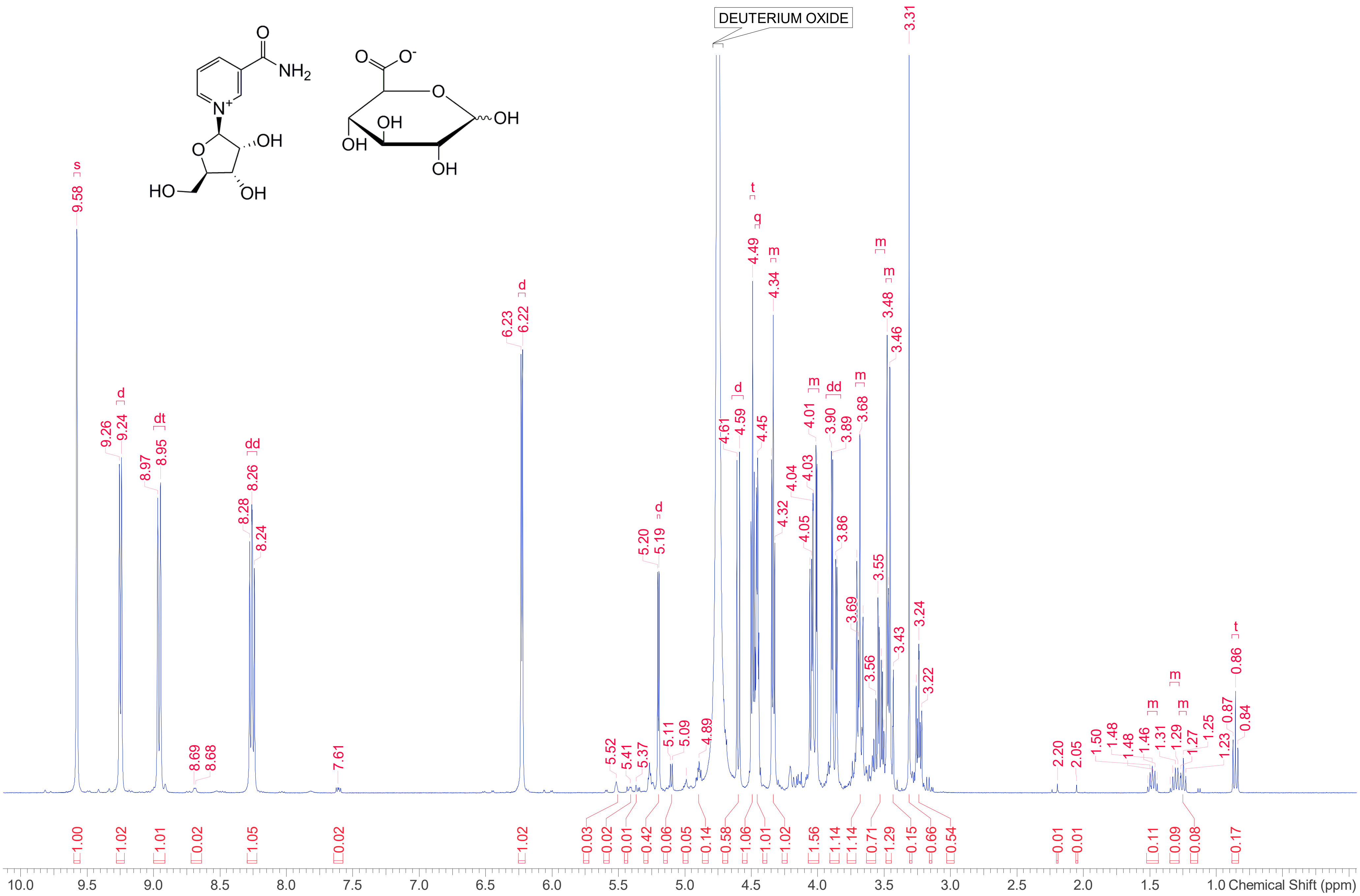

Example 4: NR D-glucuronate ^{13}C-NMR (D_2O, 100 MHz)

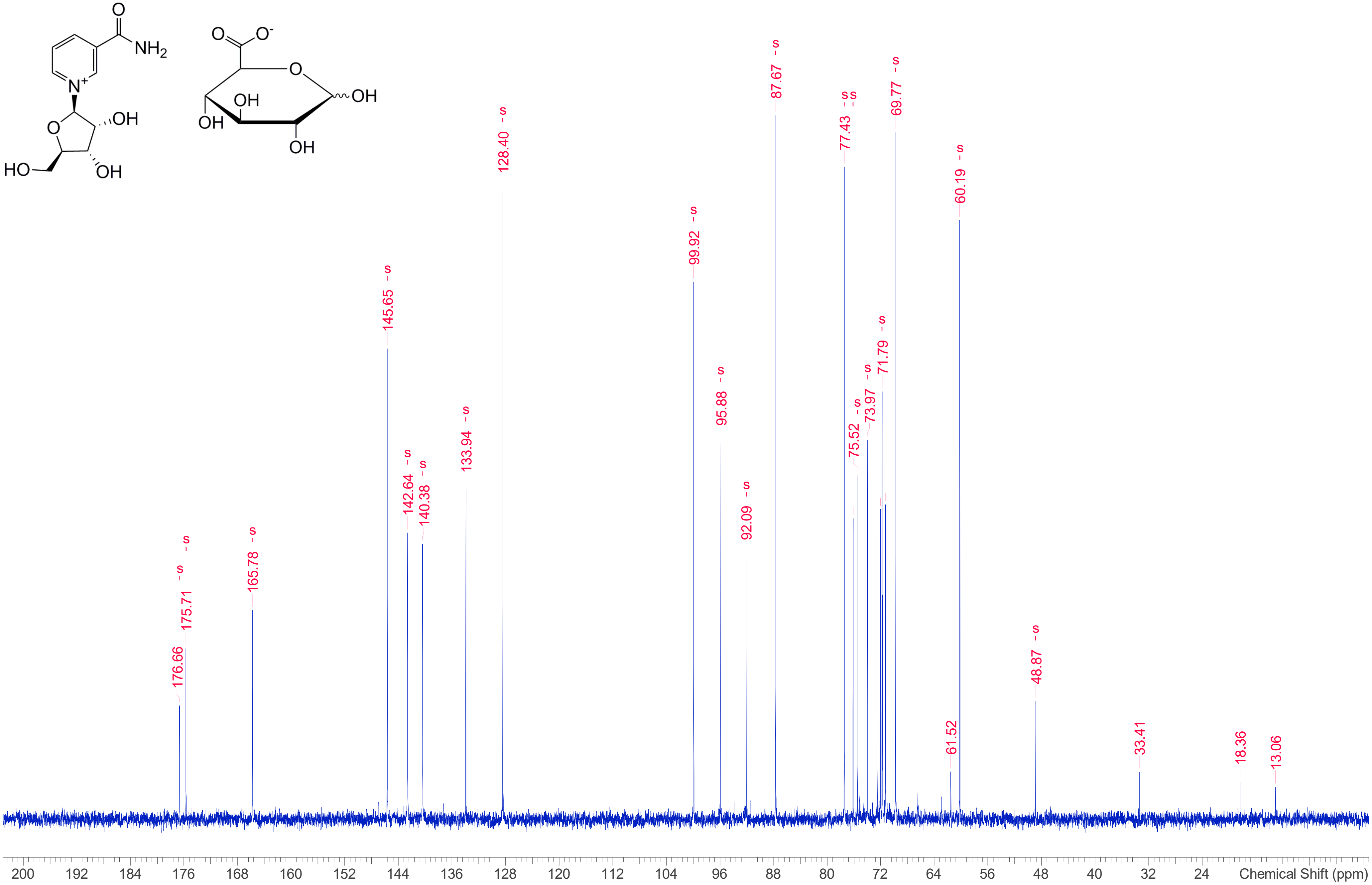

Example 5: NR bromide ^{1}H-NMR (D_2O, 400 MHz)

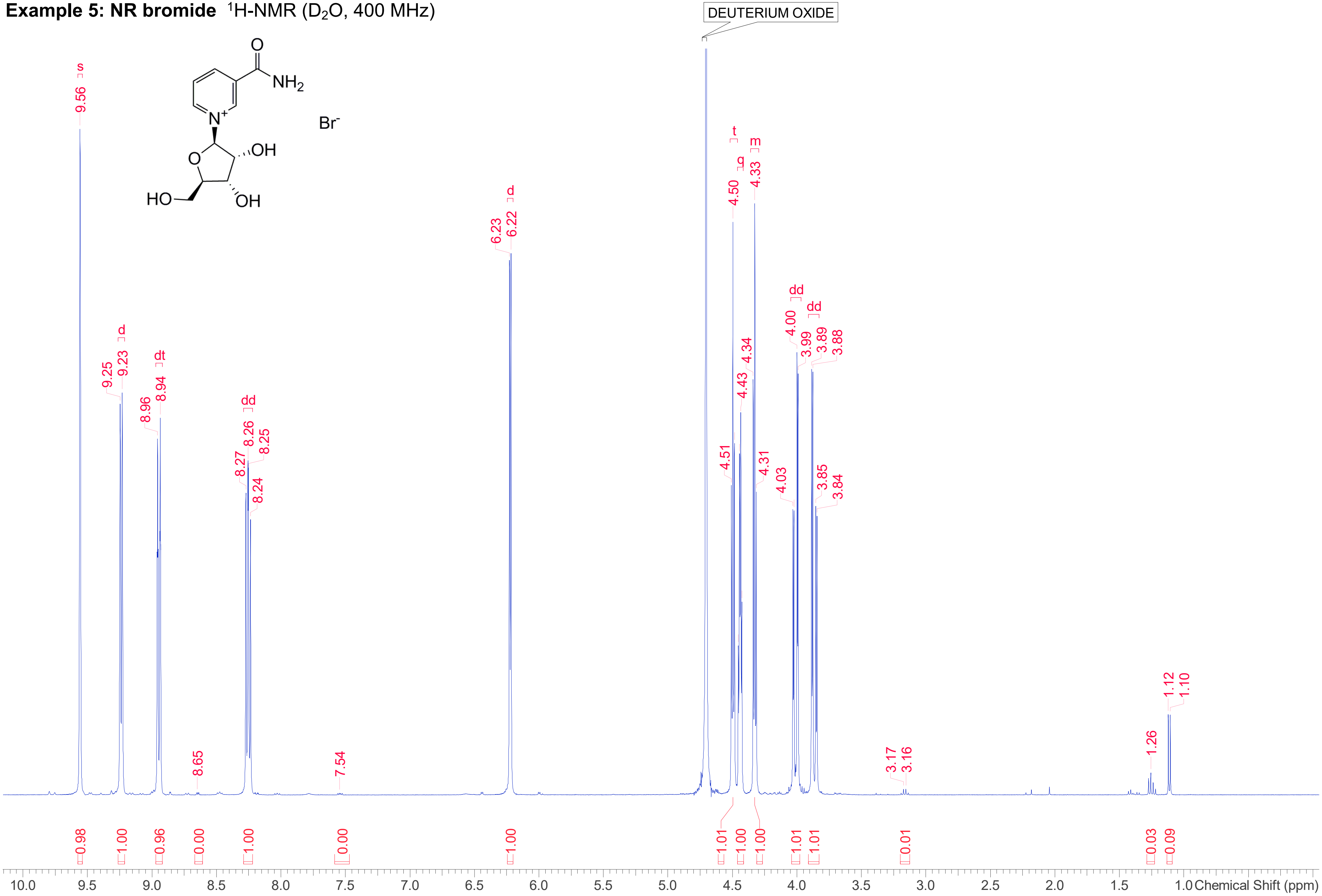

Example 5: NR bromide ^{13}C-NMR (D_2O, 100 MHz)

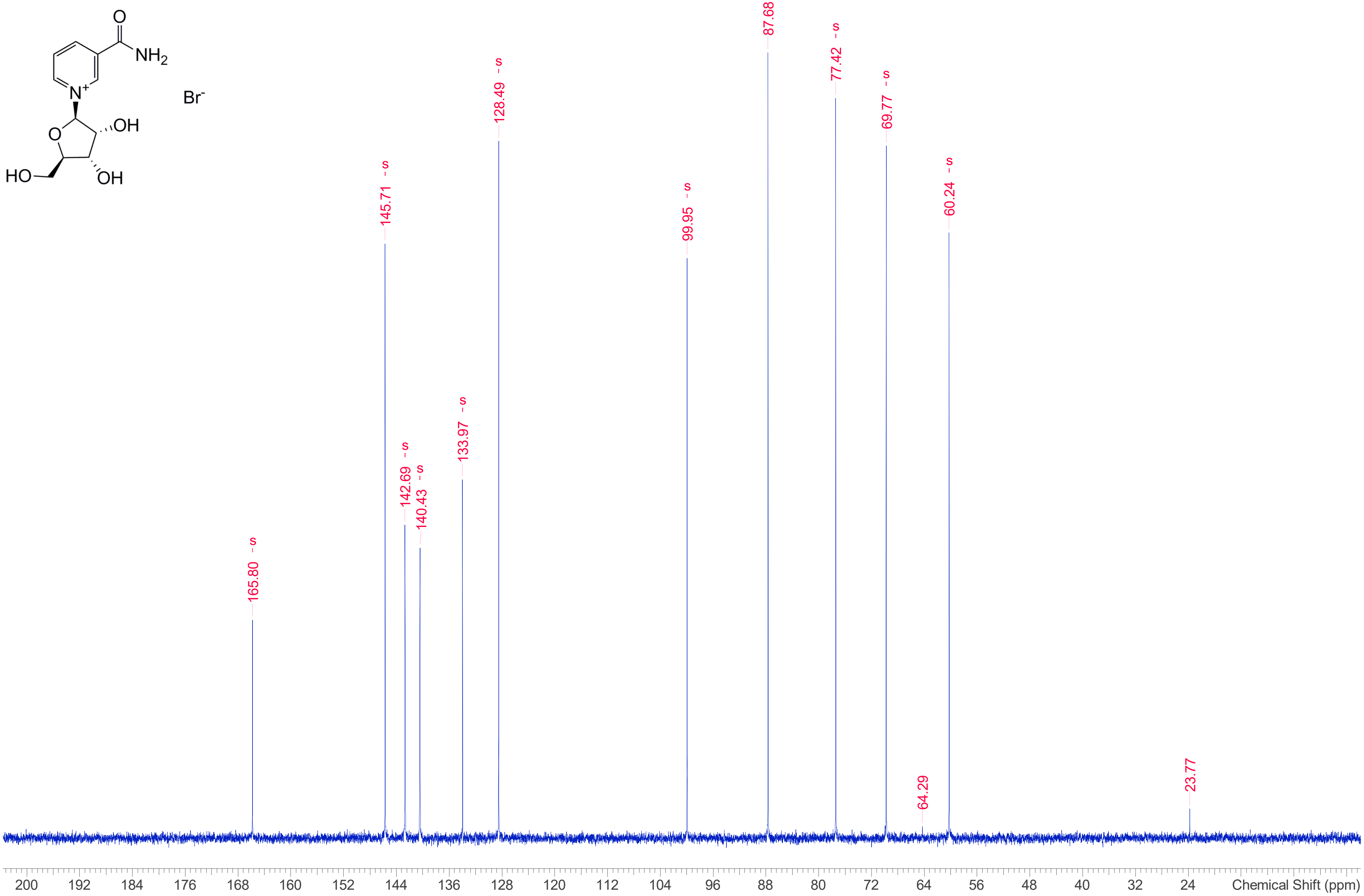

Example 6: NR L-malate ^{1}H-NMR (D_2O, 400 MHz)

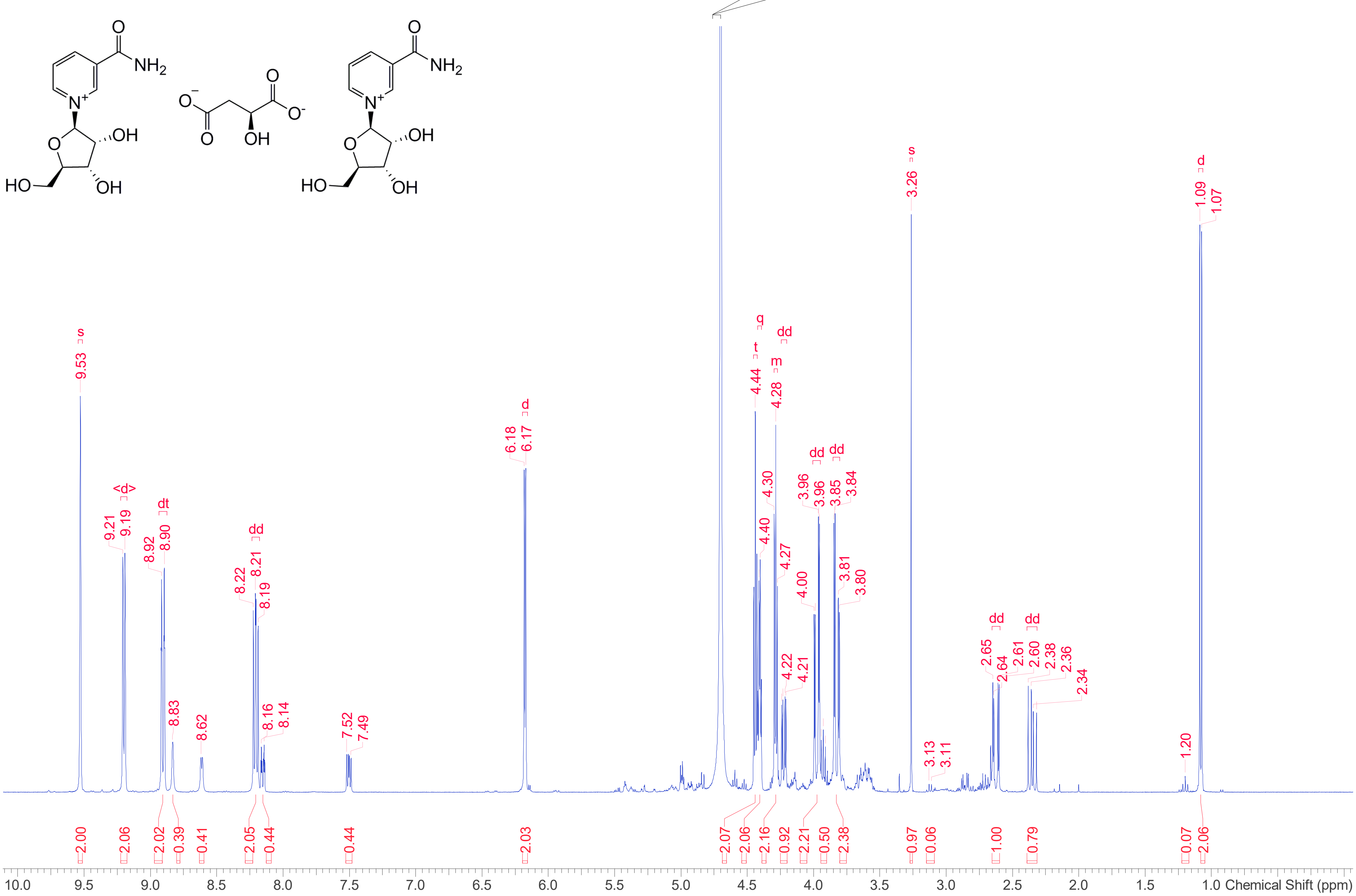

Example 6: NR L-malate ^{13}C-NMR (D_2O, 100 MHz)

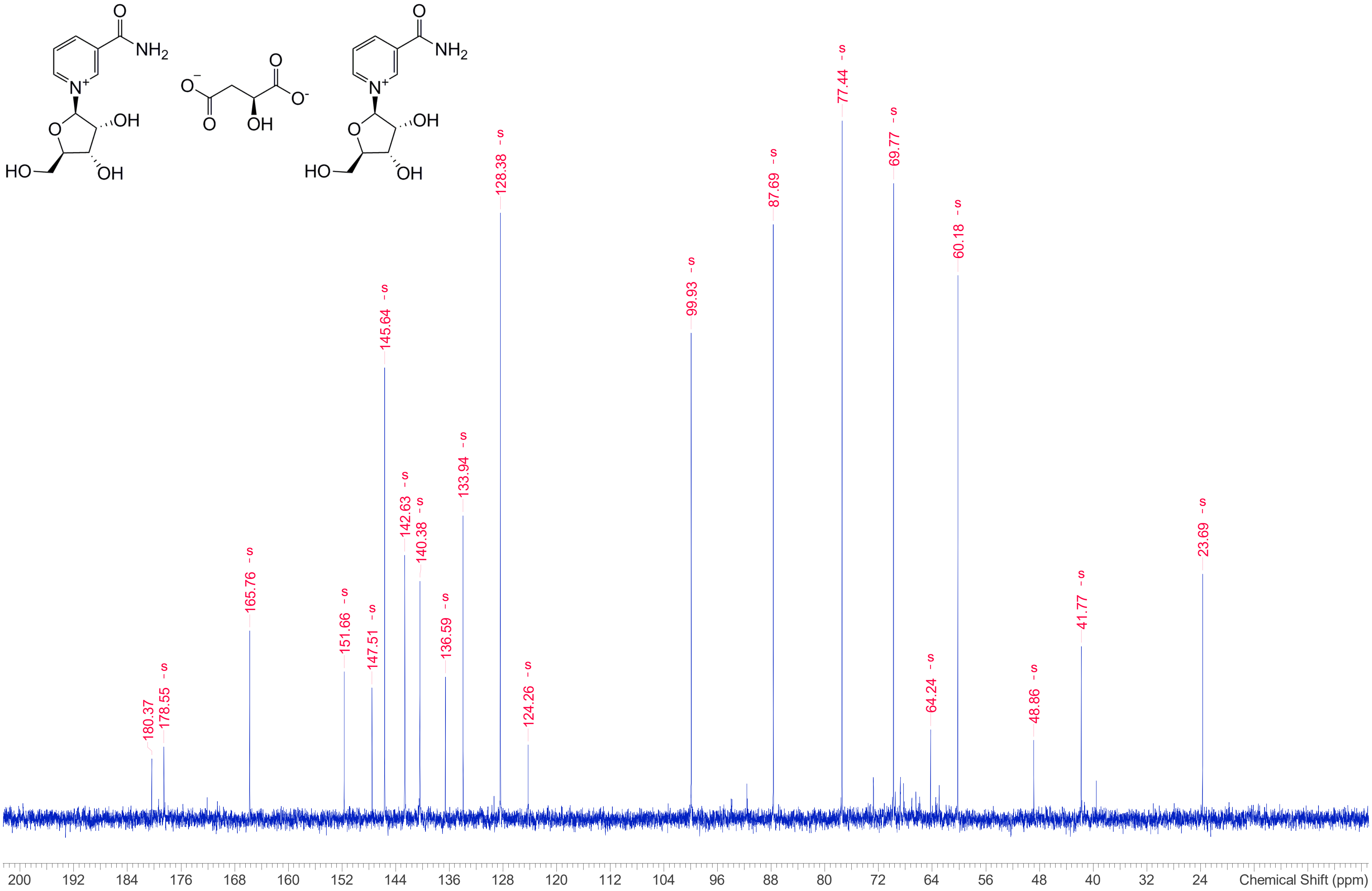

Example 7: NR L-tartrate ^{1}H-NMR (D_2O, 400 MHz)

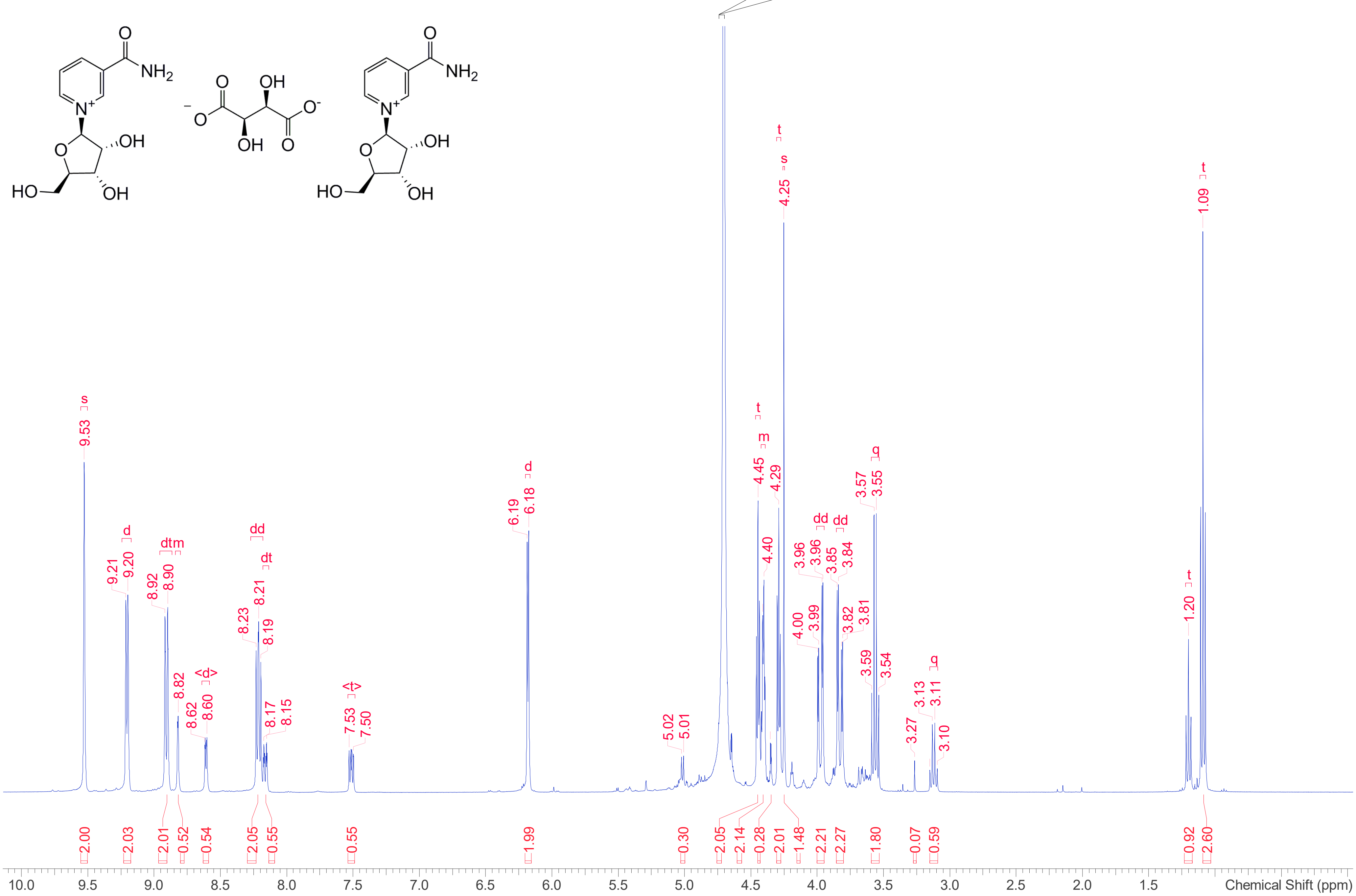

Example 7: NR L-tartrate ^{13}C-NMR (D_2O, 100 MHz)

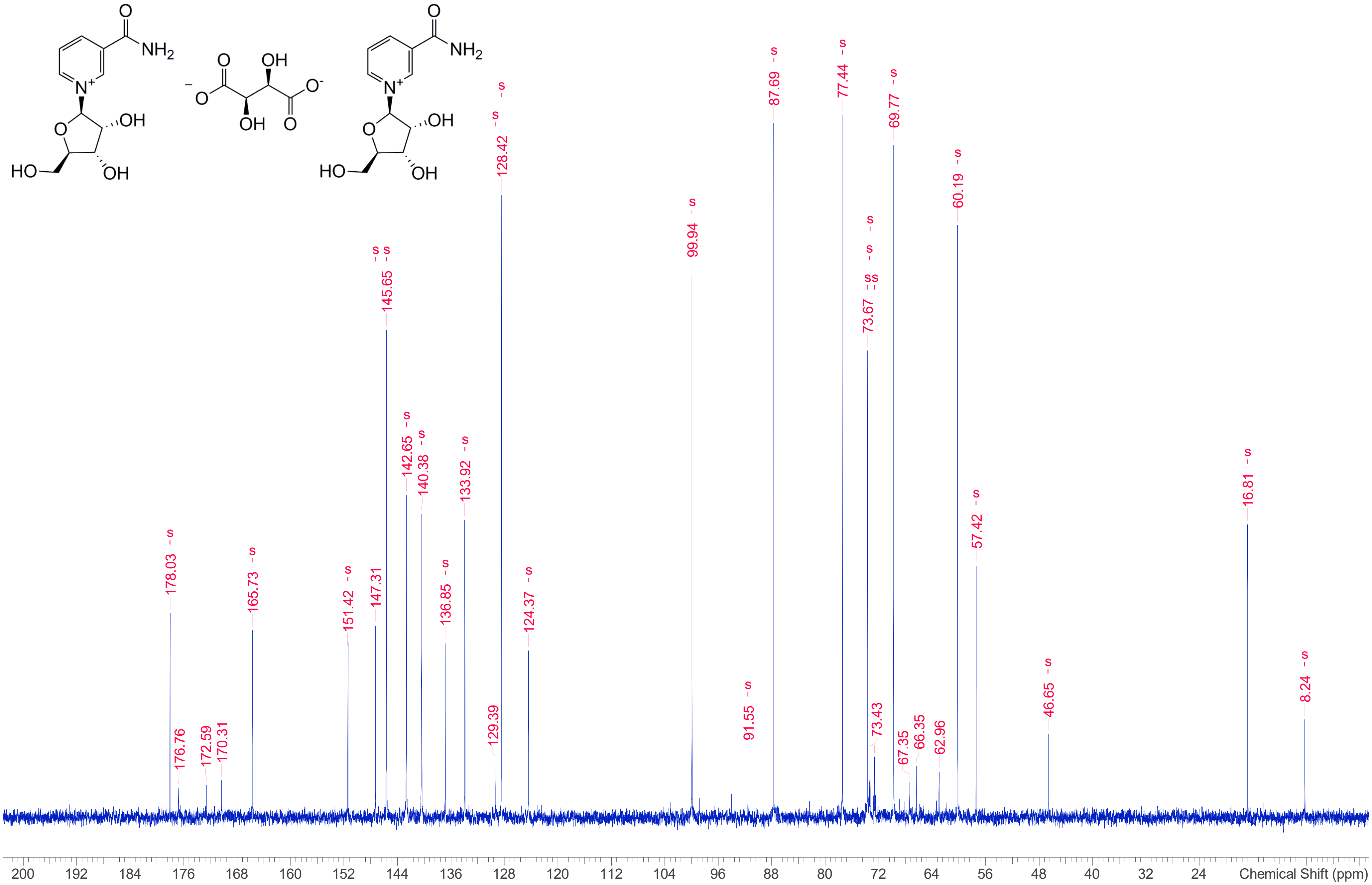

Example 9: NR meso-Hydrogentartrate ^{1}H-NMR (D_2O, 400 MHz)

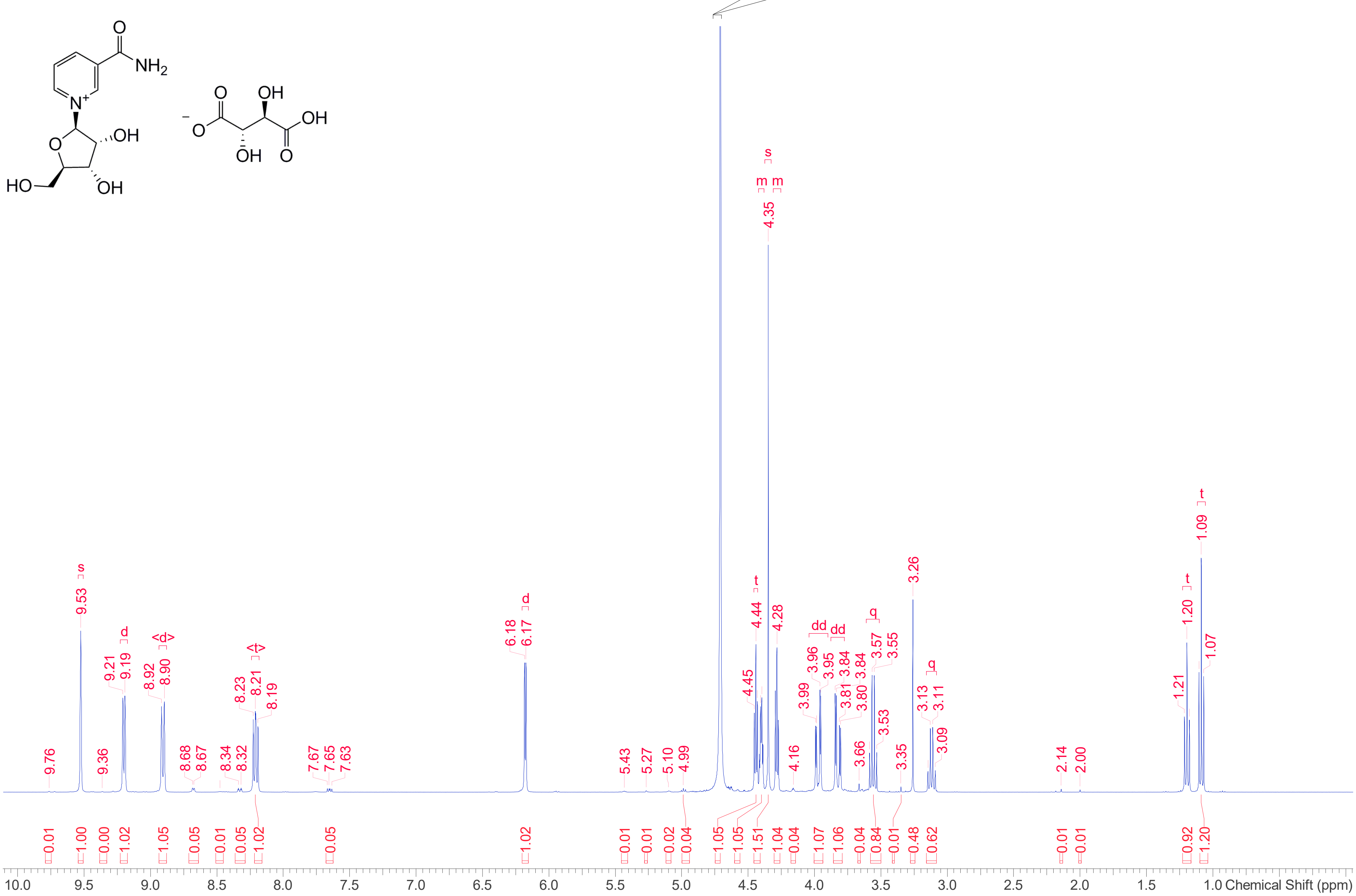

Example 9: NR meso-Hydrogentartrate ^{13}C-NMR (D_2O, 100 MHz)

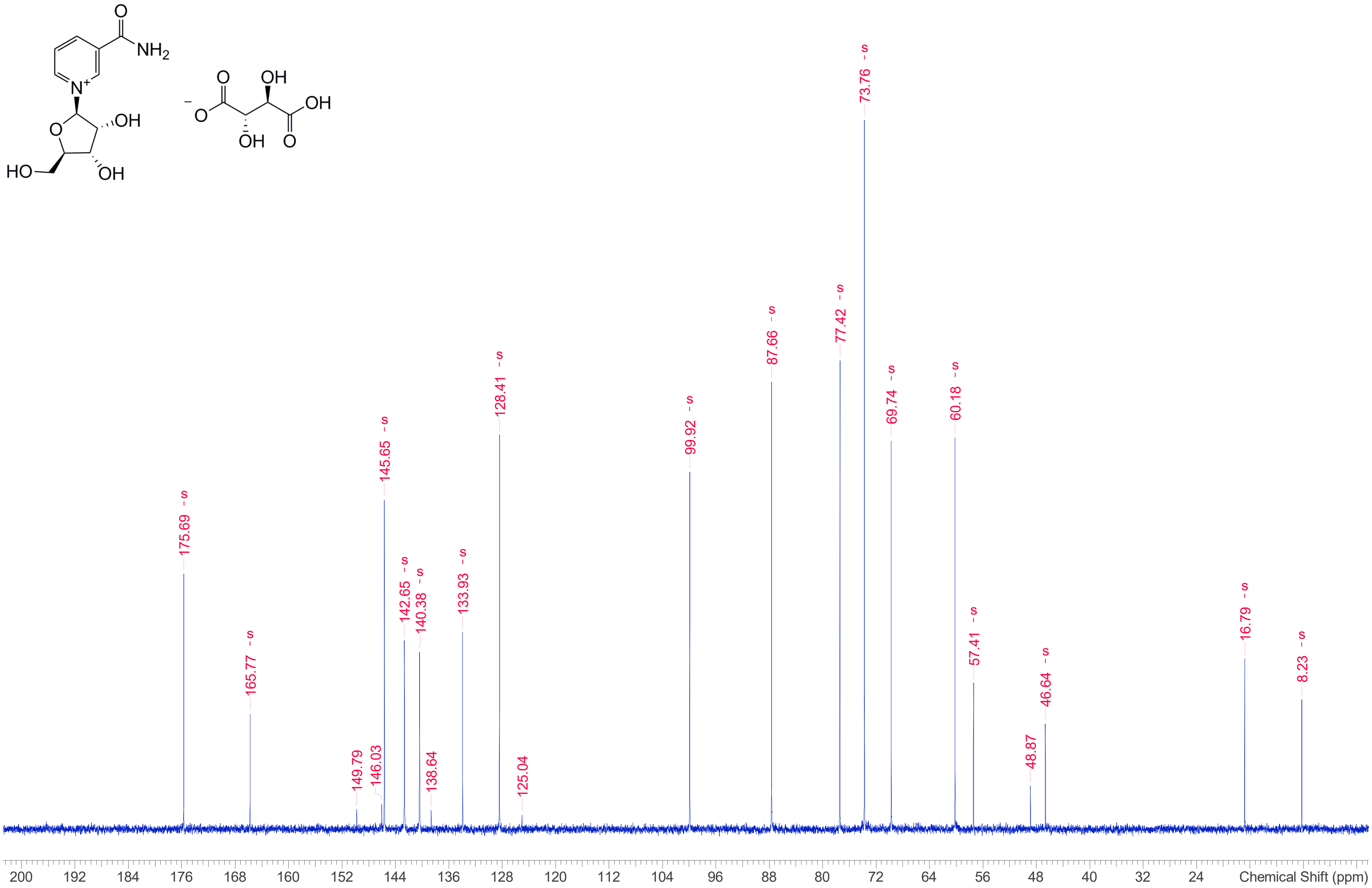

Example 10a: NR L-Hydrogentartrate ^{1}H-NMR (D_2O, 400 MHz)

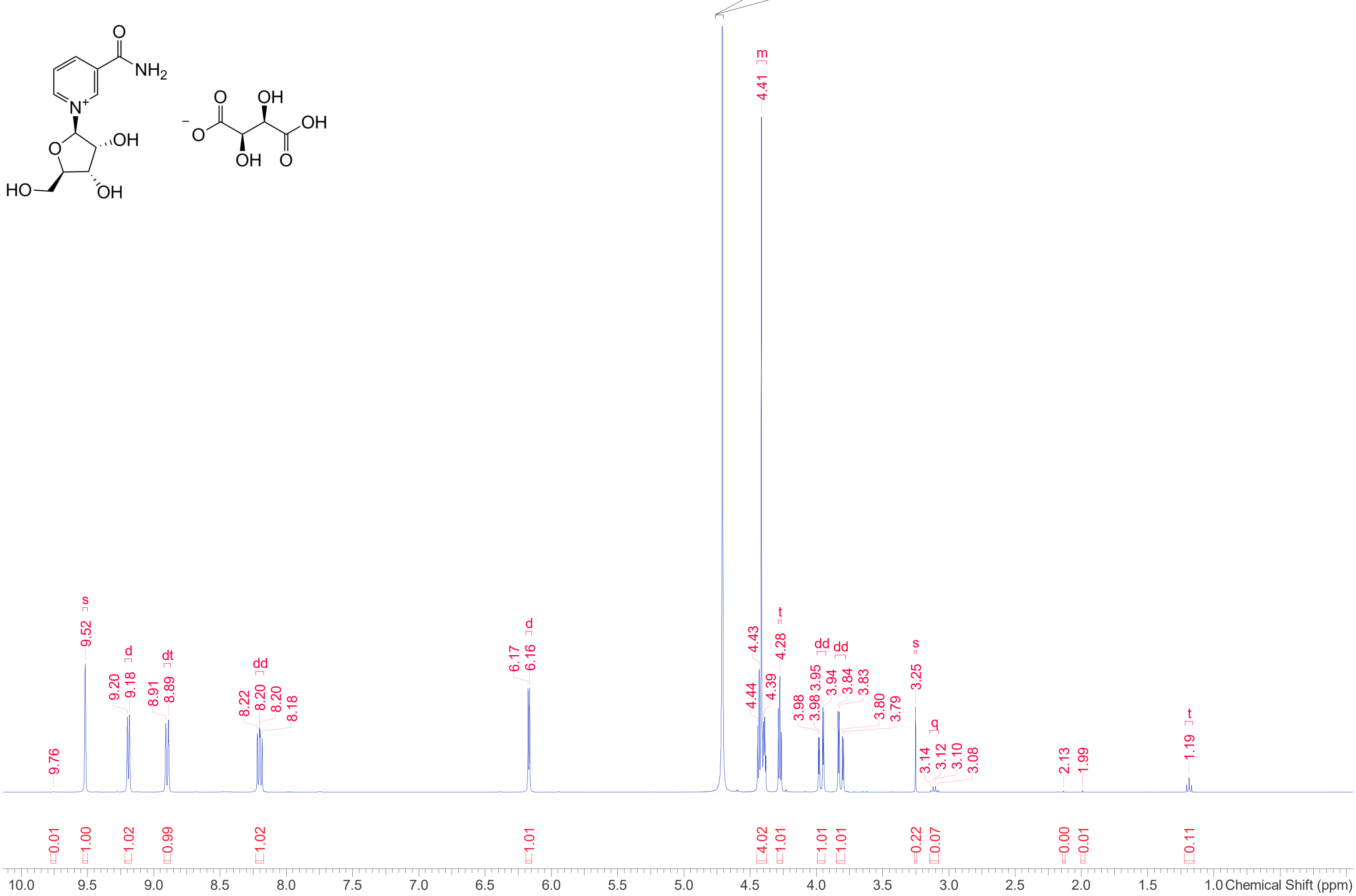

Example 10a: NR L-Hydrogentartrate ^{13}C-NMR (D_2O, 100 MHz)

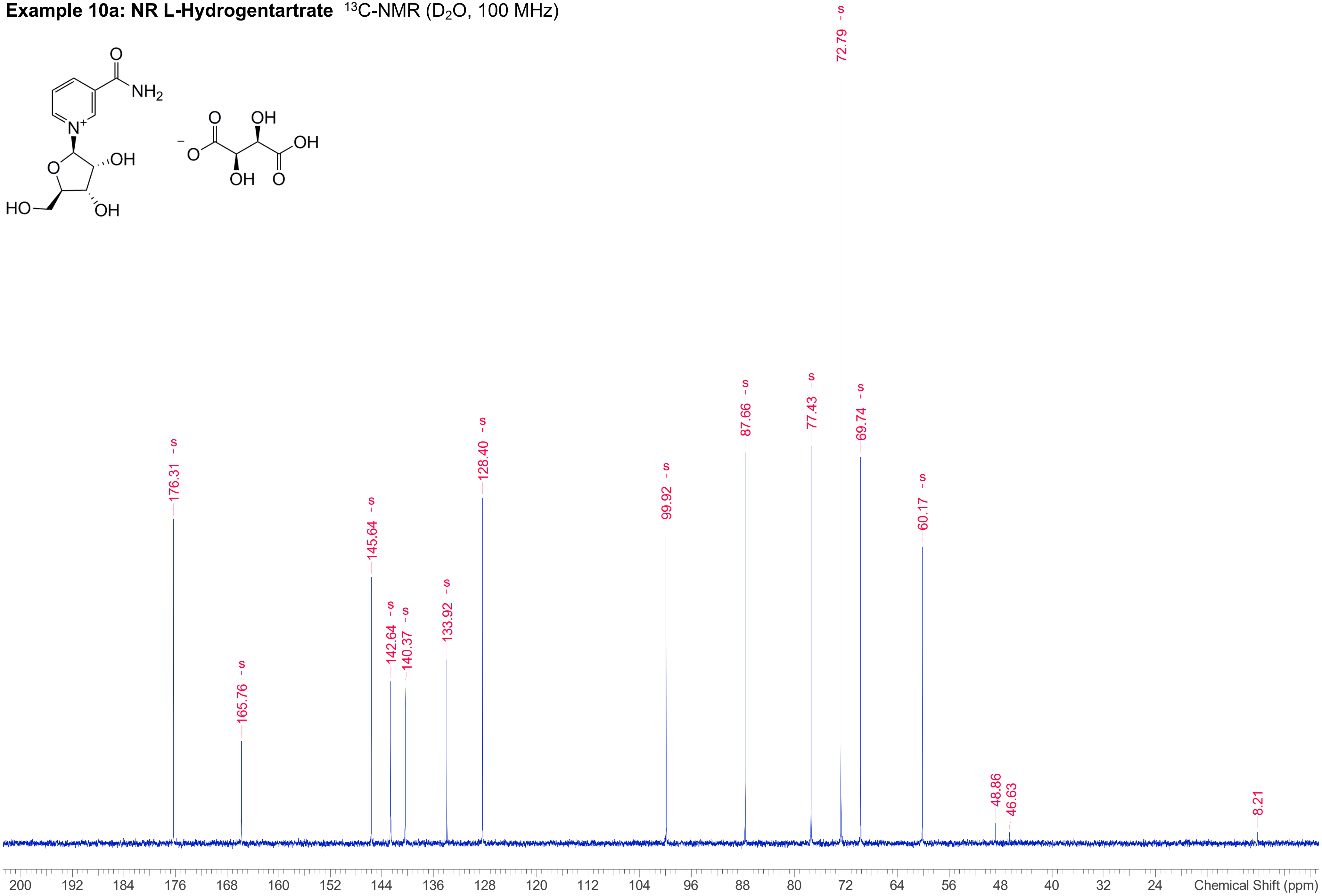

Example 11: NR DL-Hydrogentartrate ^{1}H-NMR (D_2O, 400 MHz)

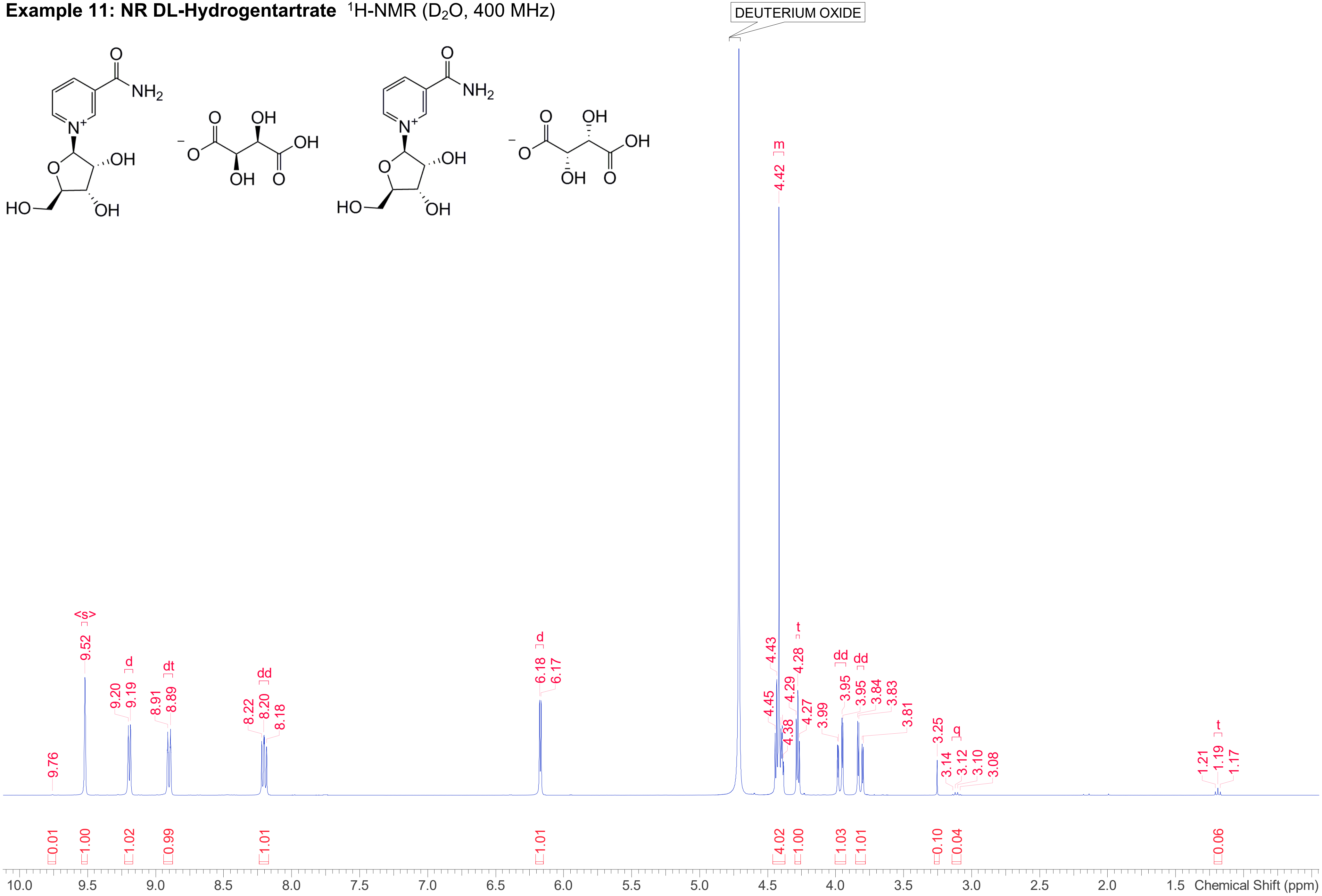

Example 11: NR DL-Hydrogentartrate ^{13}C-NMR (D_2O, 100 MHz)

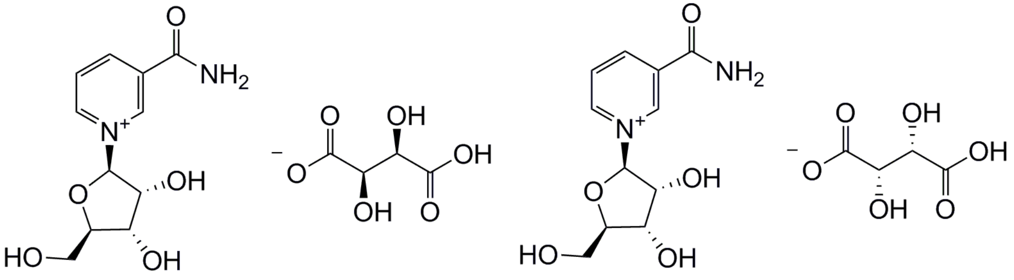

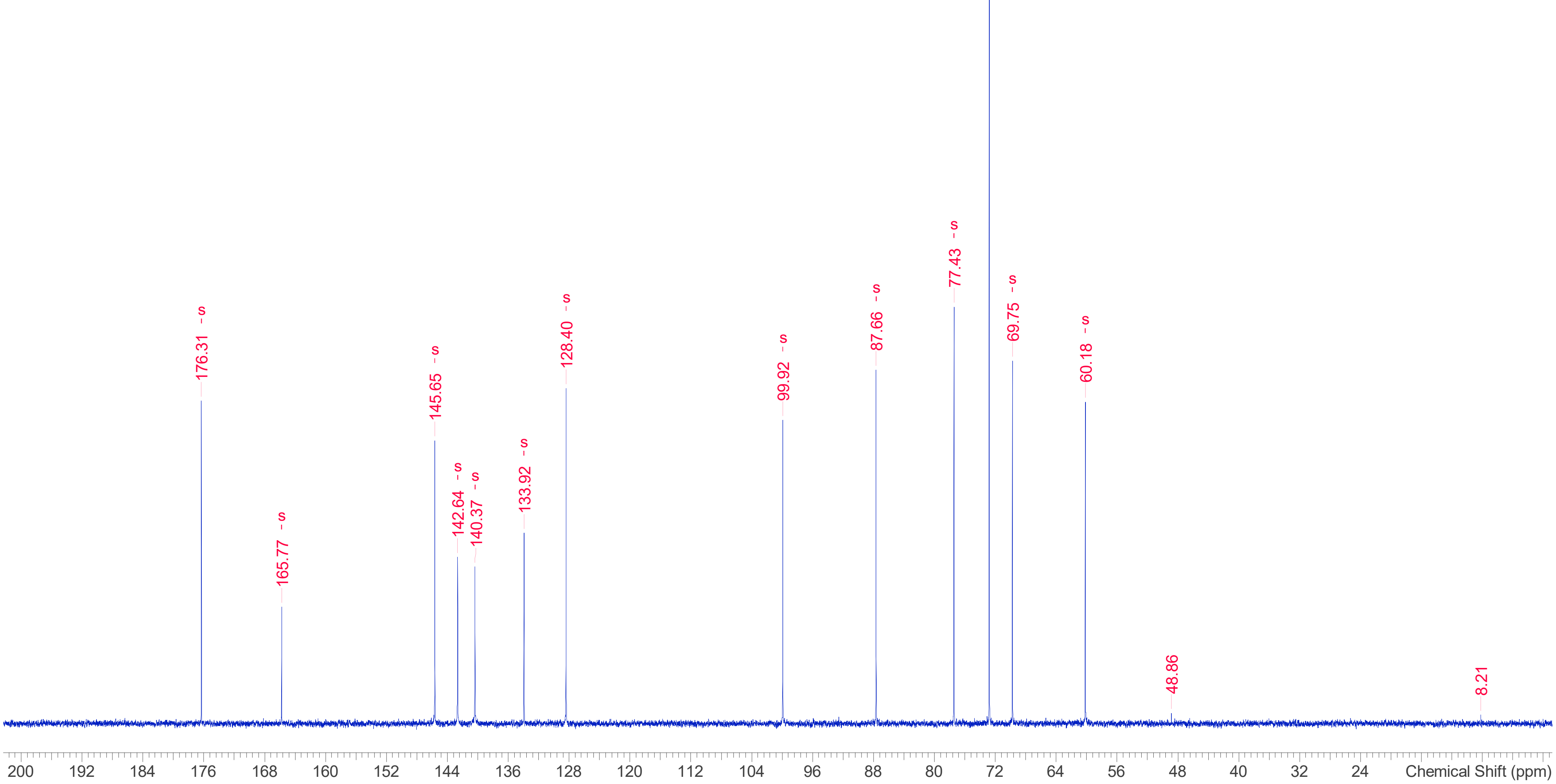

Example 12a: NR L-Hydrogenmalate ^{1}H-NMR (D_2O, 400 MHz)

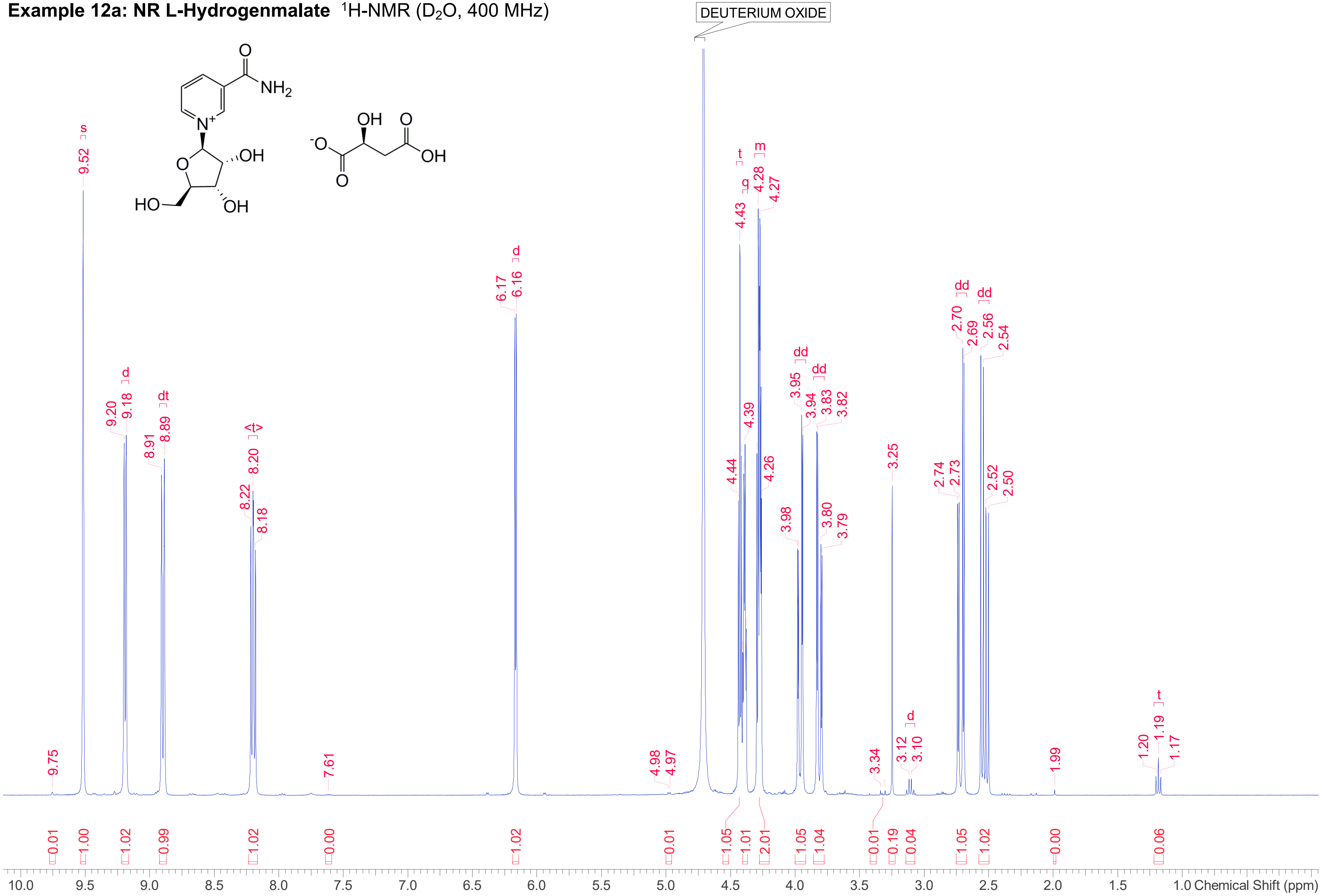

Example 12a: NR L-Hydrogenmalate ^{13}C-NMR (D_2O, 100 MHz)

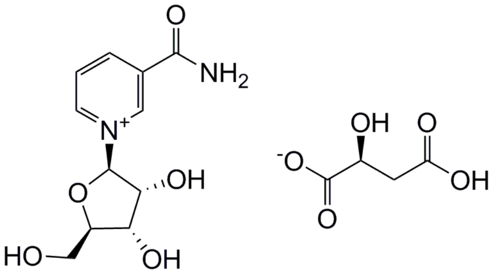

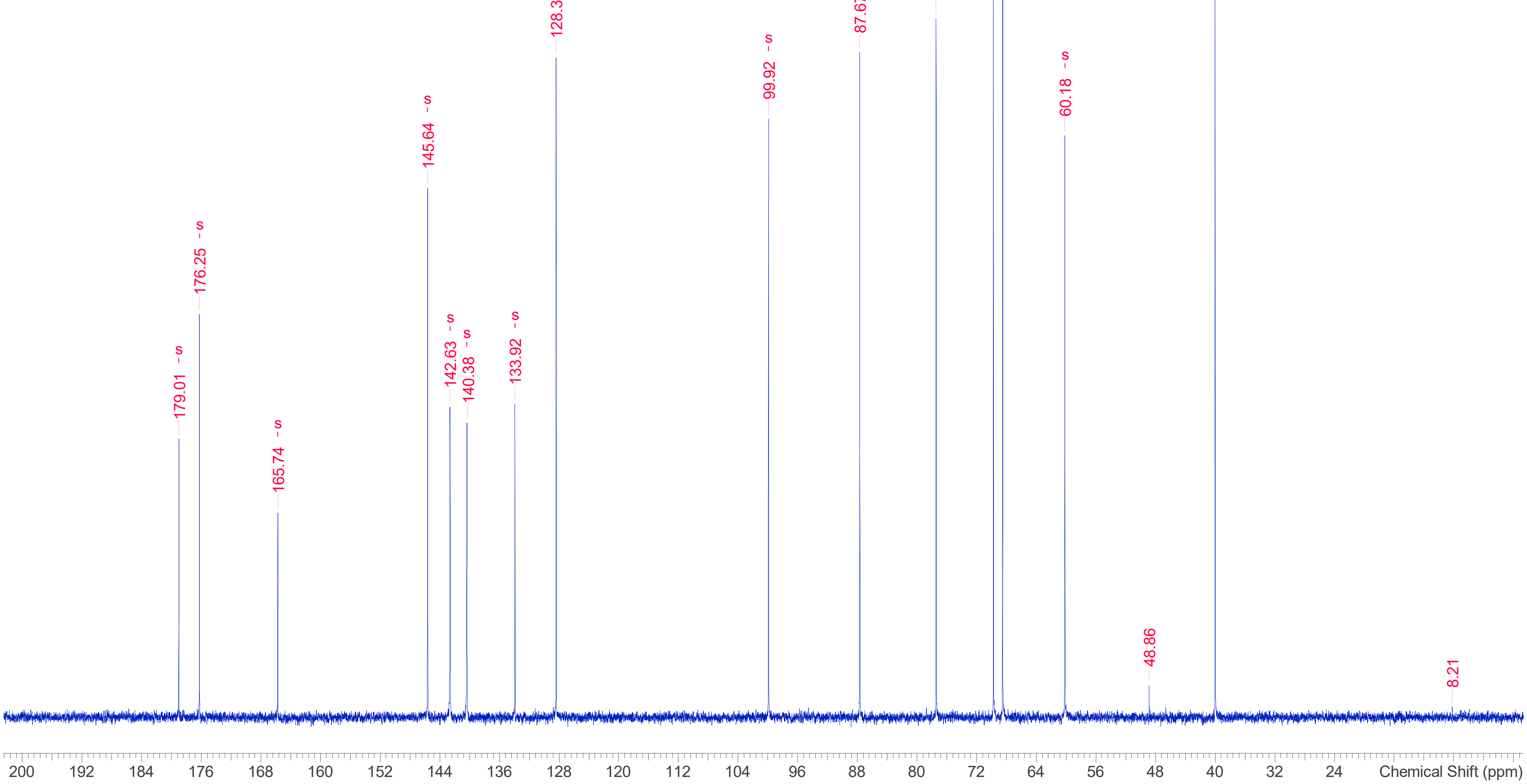

Example 13: NR D-Hydrogenmalate ^{1}H-NMR (D_2O, 400 MHz)

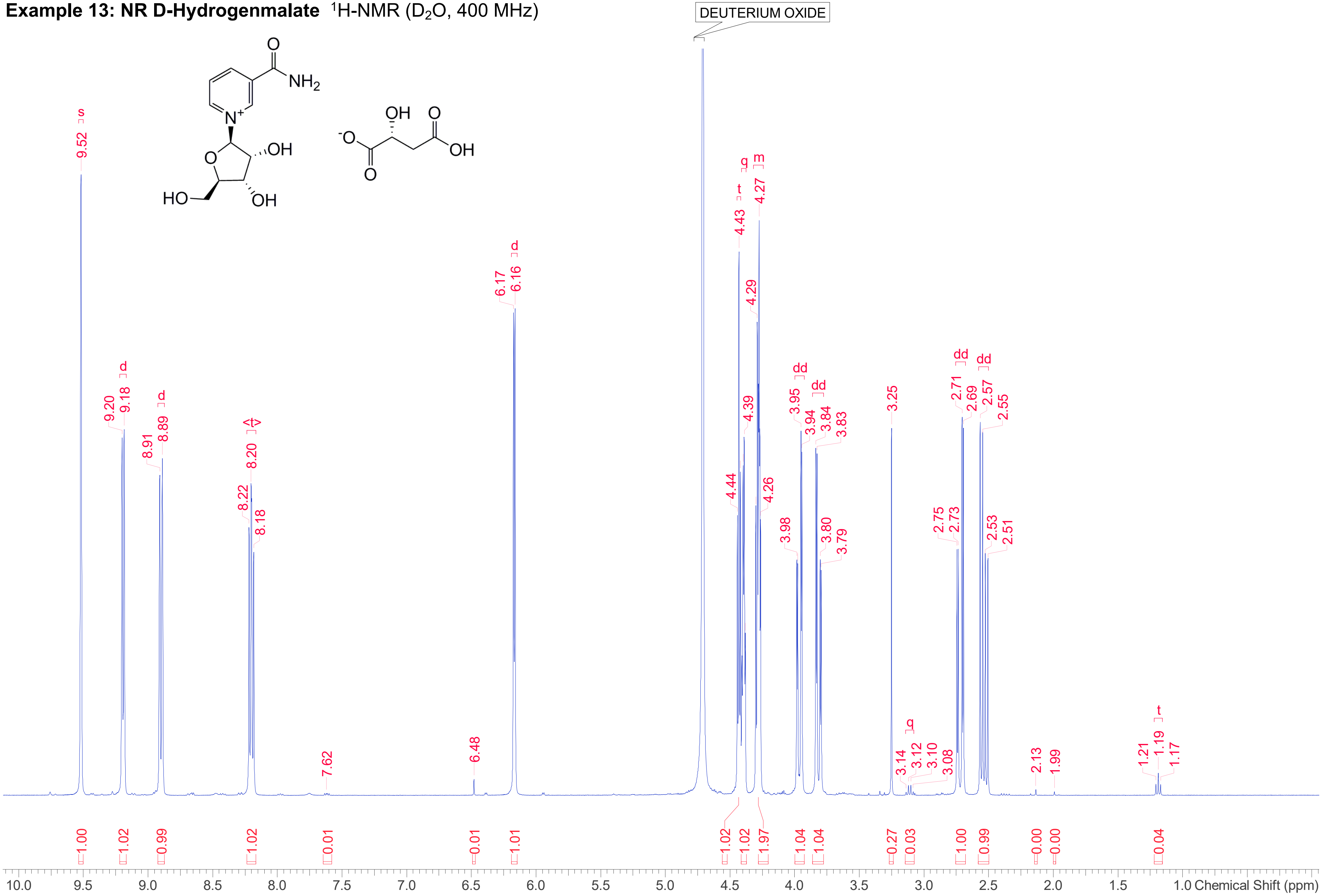

Example 13: NR D-Hydrogenmalate ^{13}C-NMR (D_2O, 100 MHz)

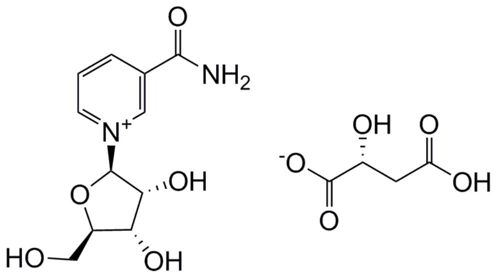

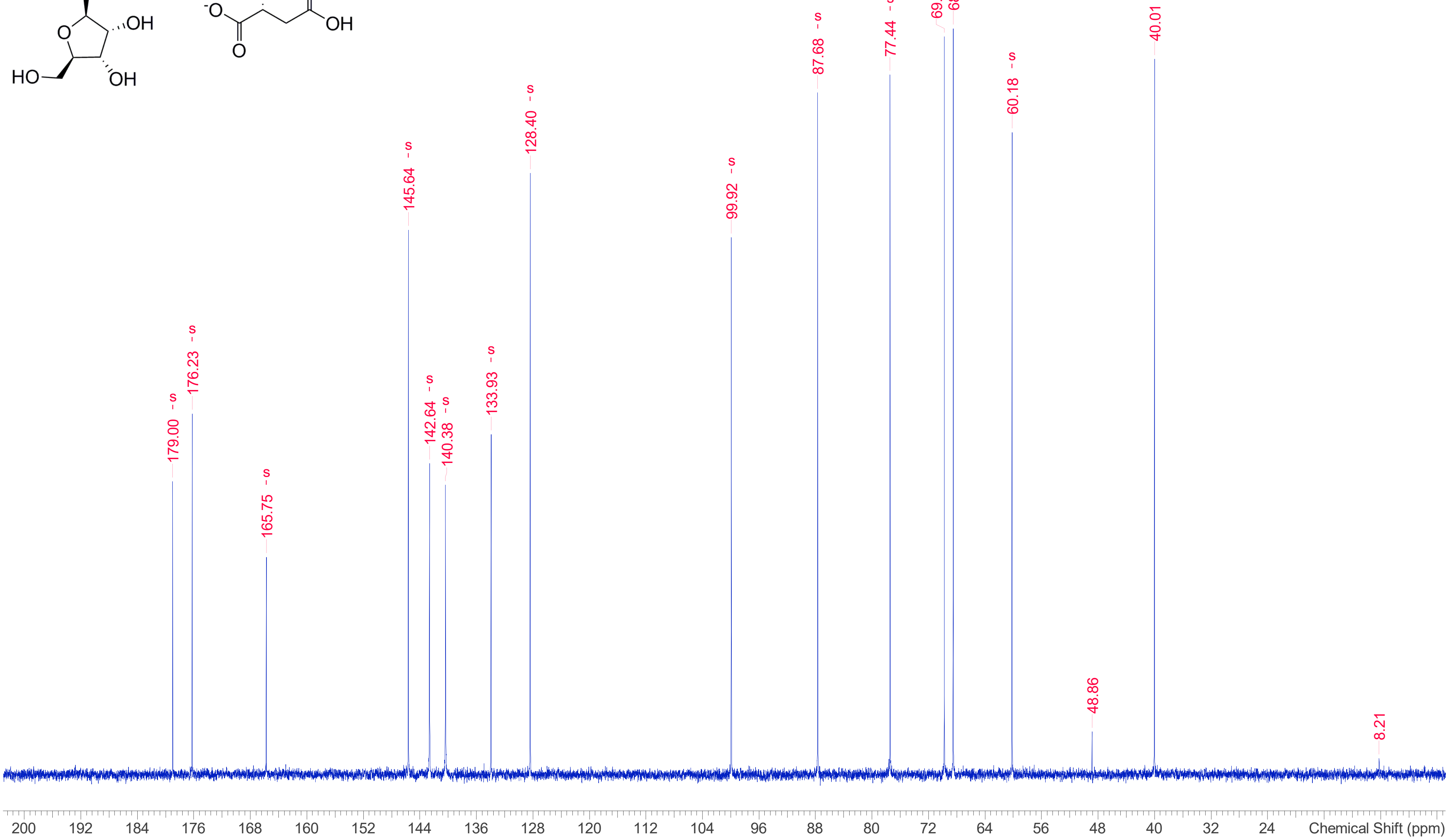

Example 13: NR DL-Hydrogenmalate ^{1}H-NMR (D_2O, 400 MHz)

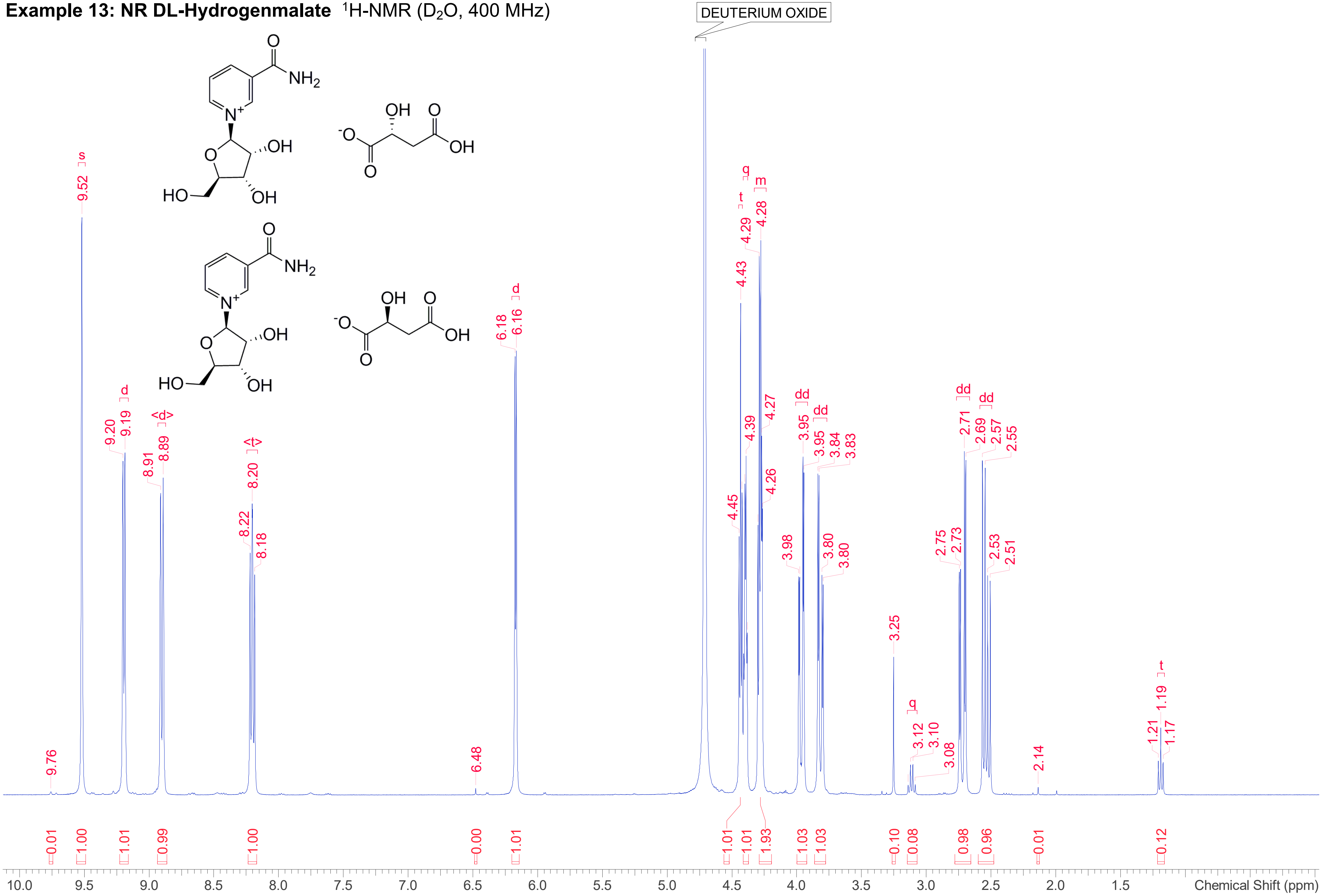

Example 13: NR DL-Hydrogenmalate ^{13}C-NMR (D_2O, 100 MHz)

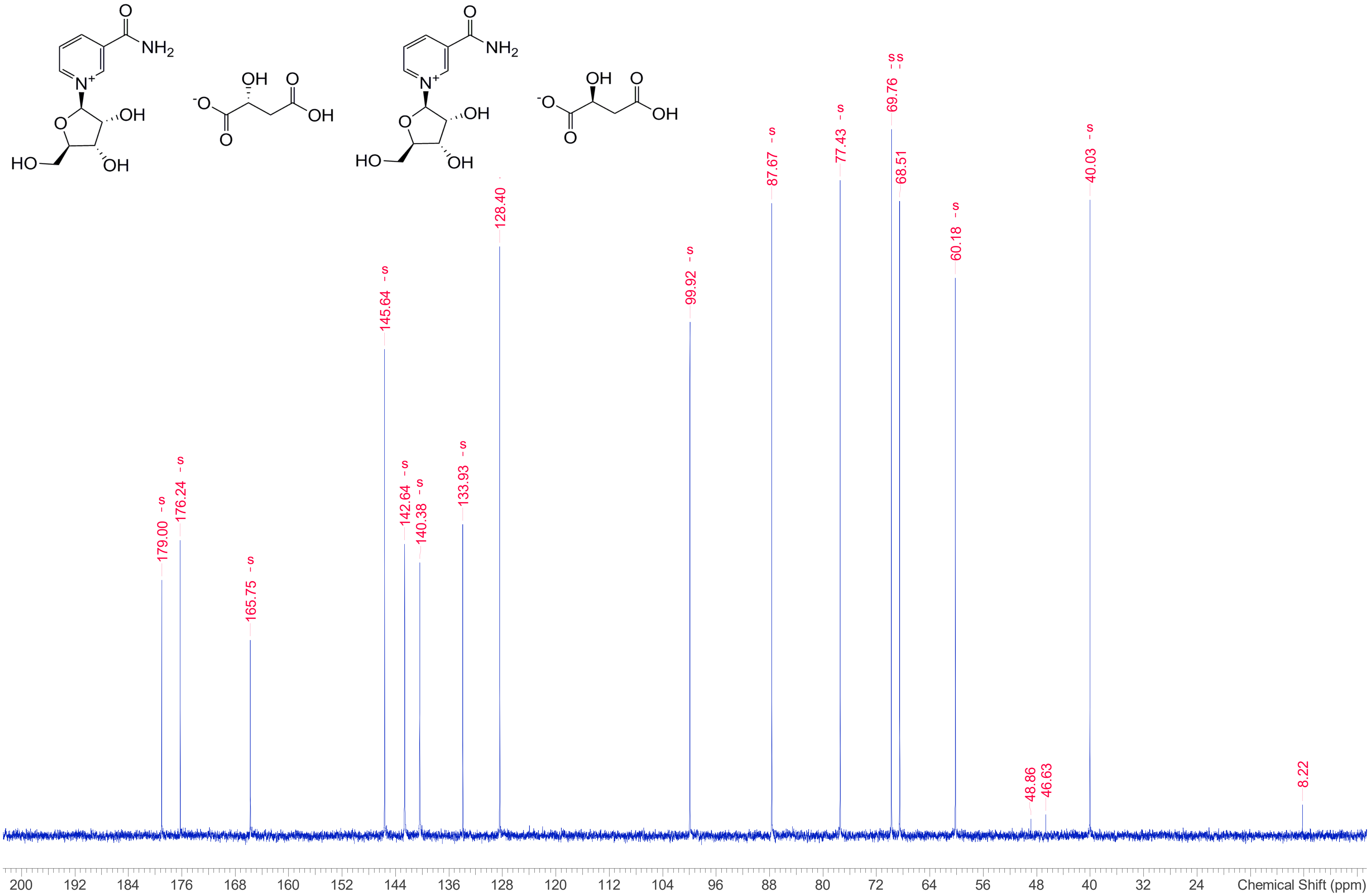

Example 13: NR D-Hydrogentartrate ^{1}H-NMR (D_2O, 400 MHz)

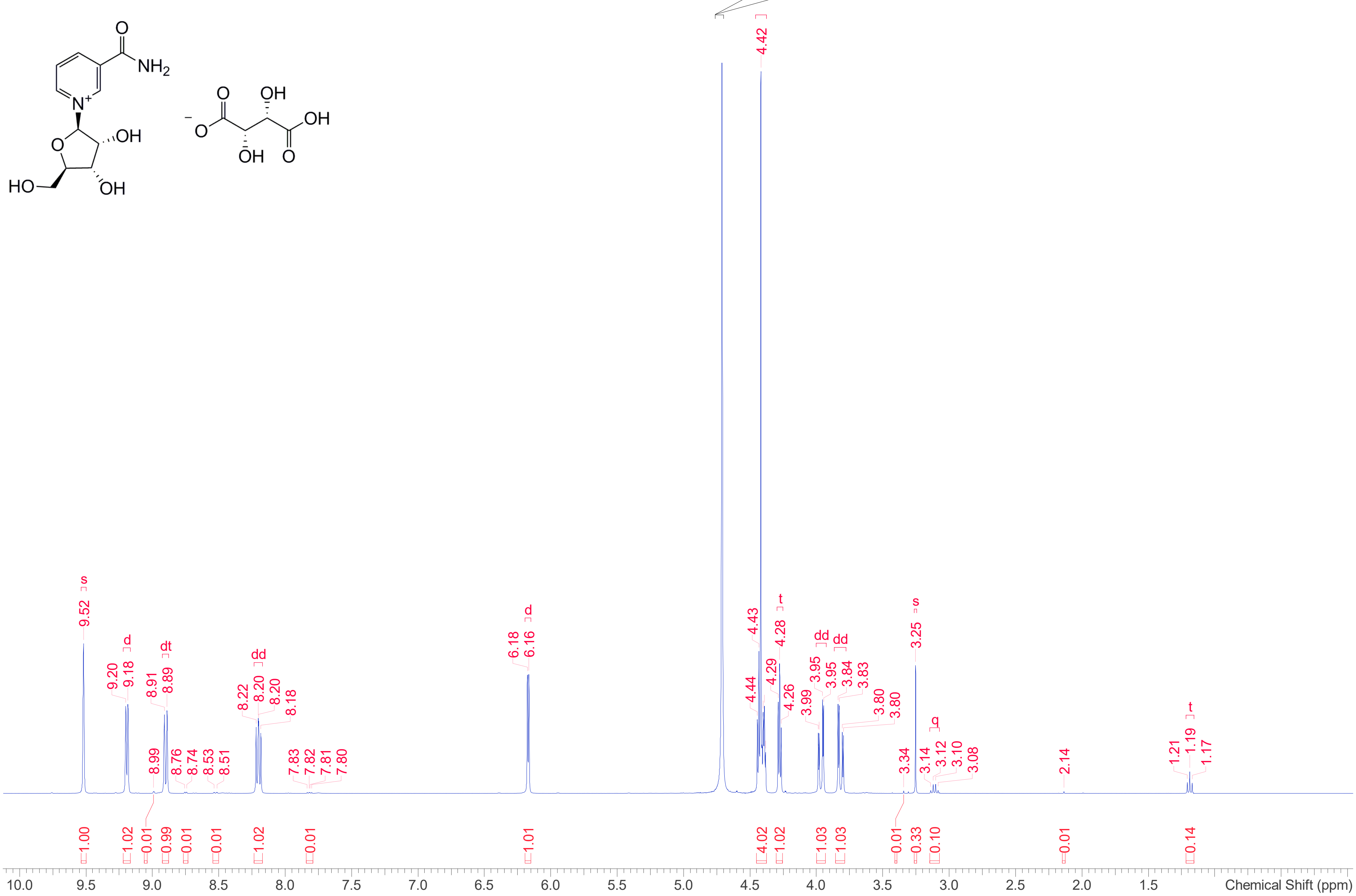

Example 13: NR D-Hydrogentartrate ^{13}C-NMR (D_2O, 100 MHz)

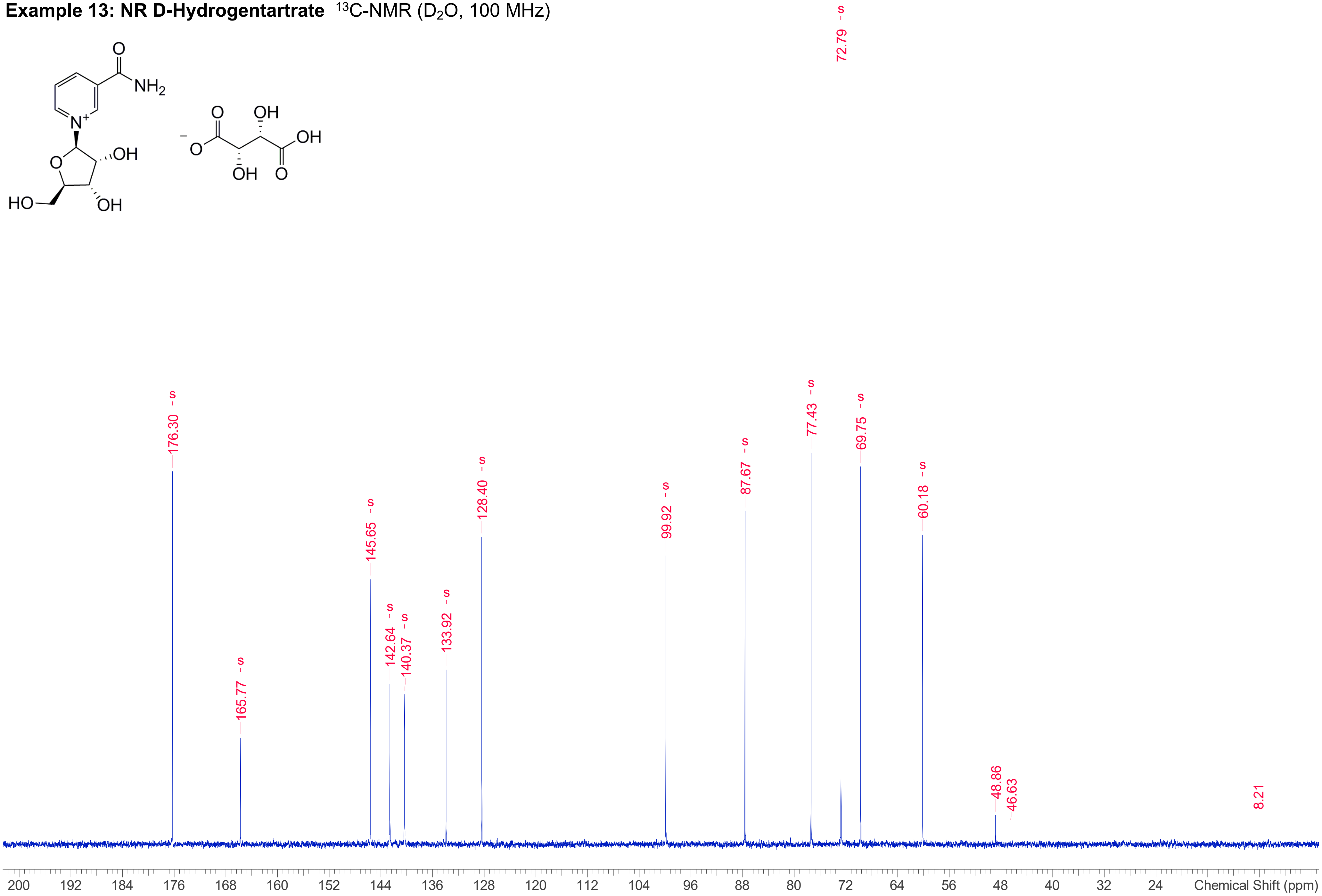

Example 14a: NR-2,3,5-triacetate triflate ^{1}H-NMR (D_2O, 400 MHz)

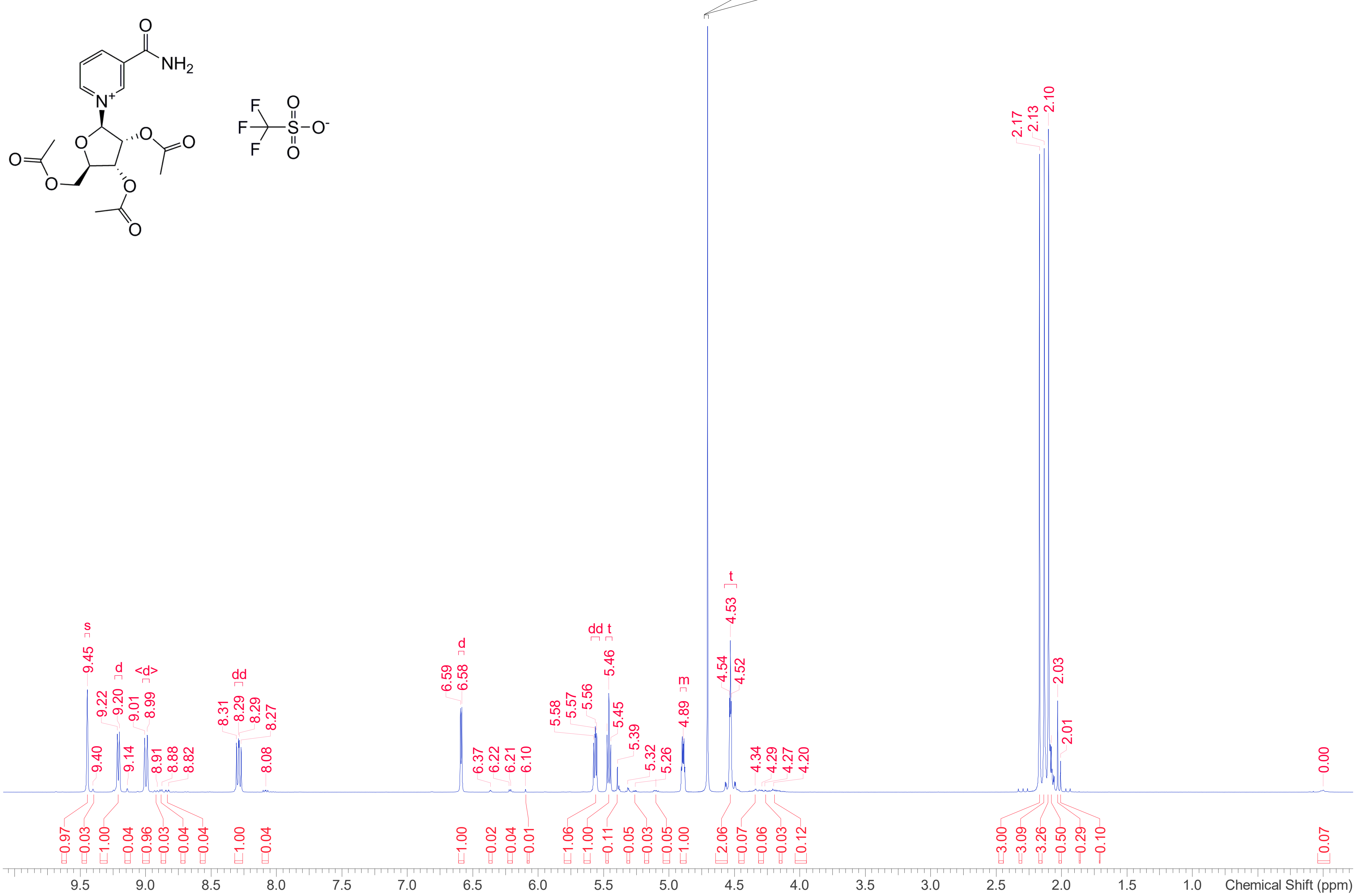

Example 14a: NR-2,3,5-triacetate triflate ^{13}C-NMR (D_2O, 100 MHz)

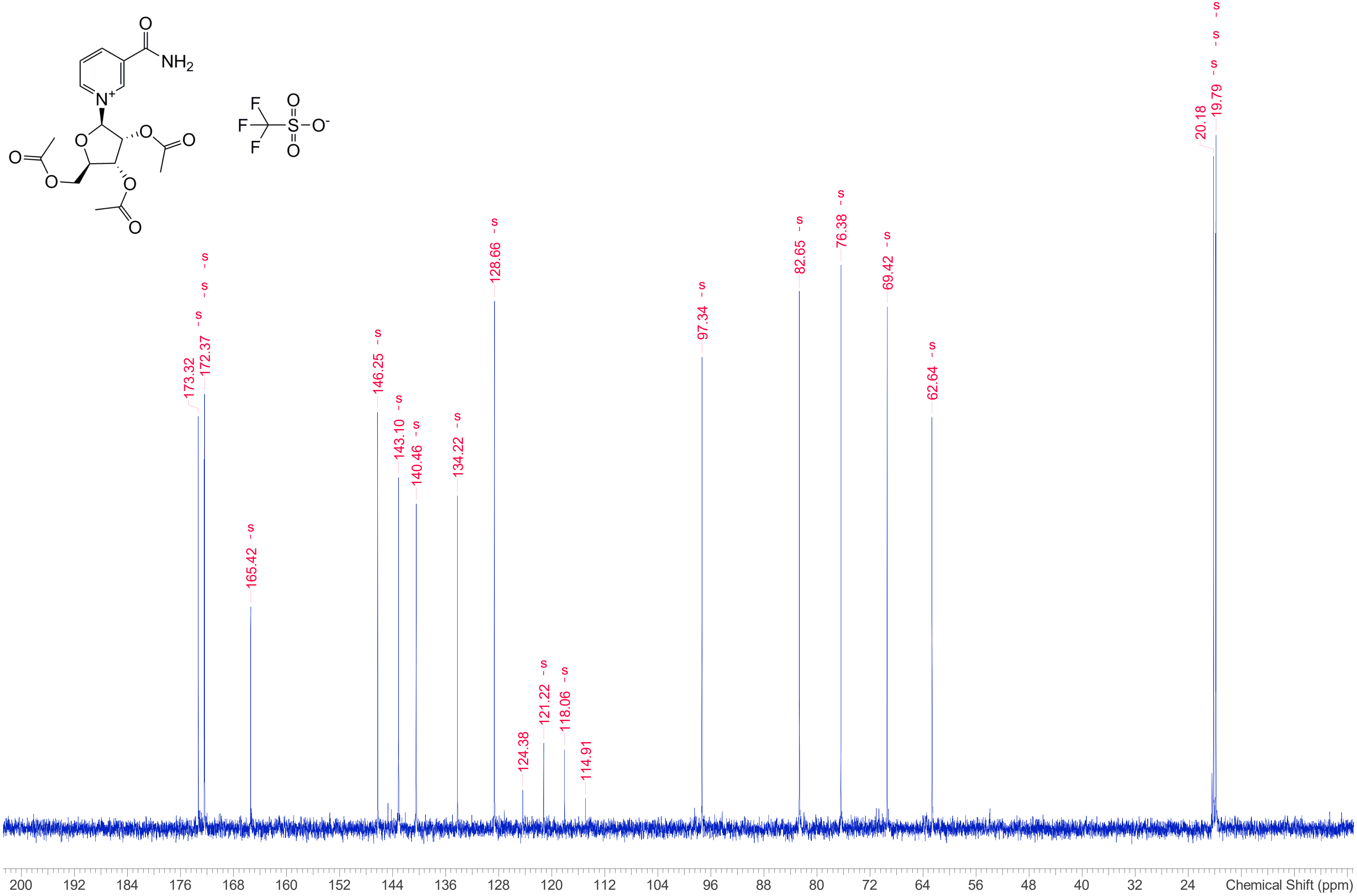

Example 15a: NR L-Hydrogentartrate ^{1}H-NMR (D_2O, 400 MHz)

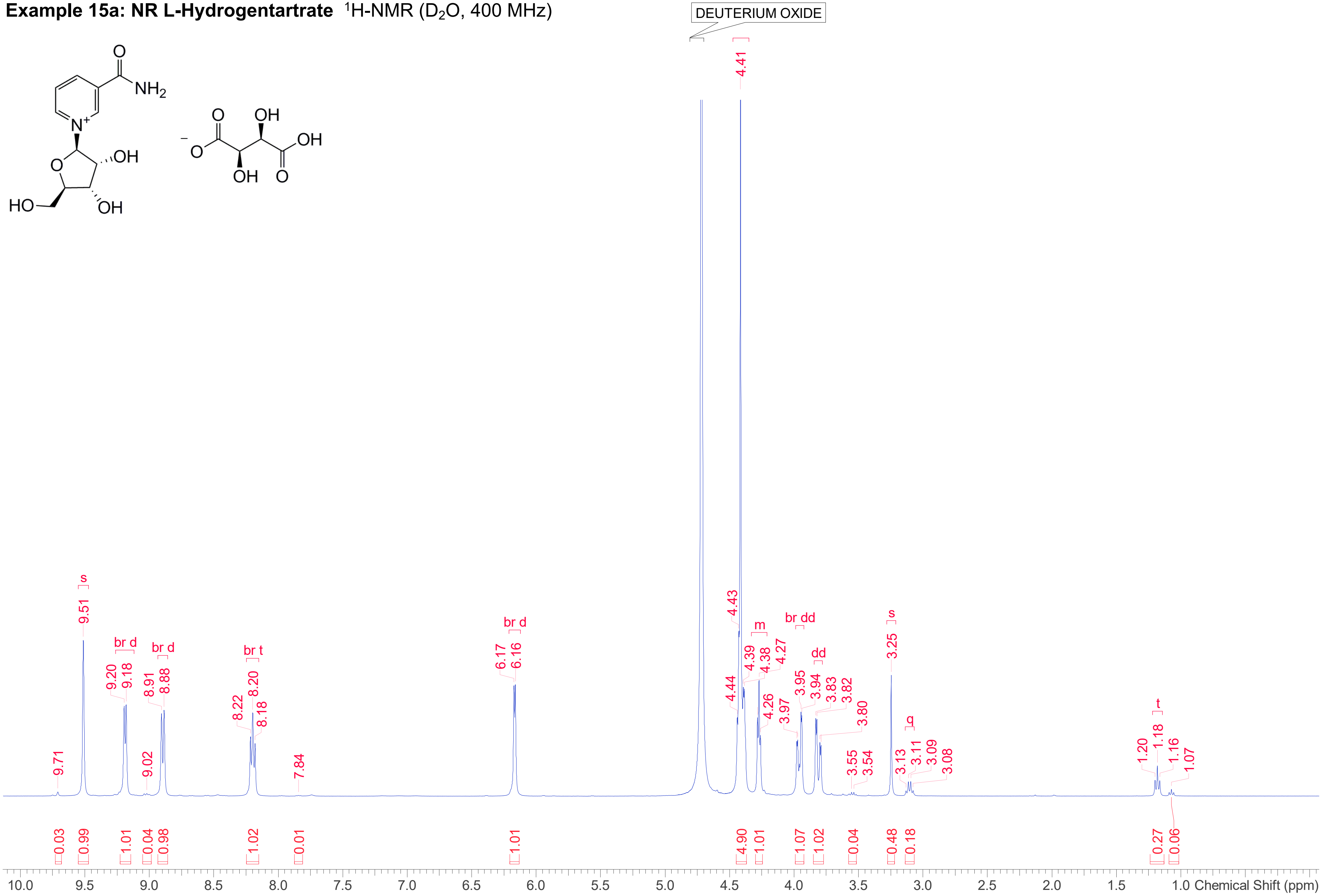

Example 15a: NR L-Hydrogentartrate ^{13}C-NMR (D_2O, 100 MHz)

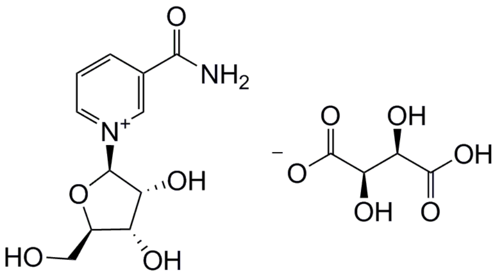

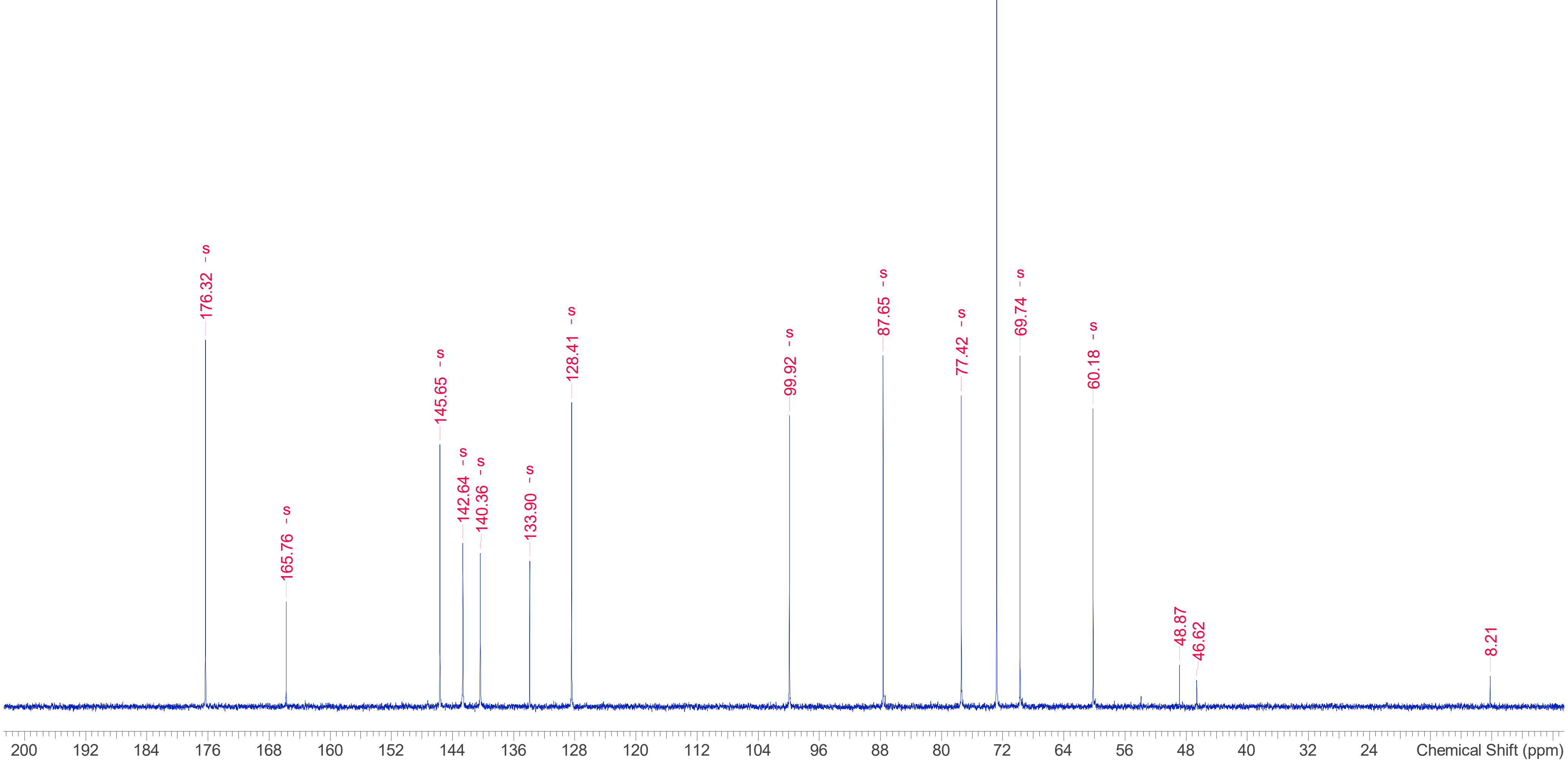

Example 15c: NR L-Hydrogentartrate ^{1}H-NMR (D_2O, 400 MHz)

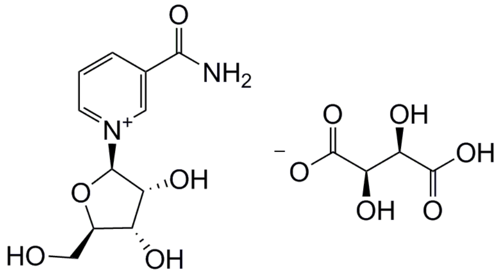

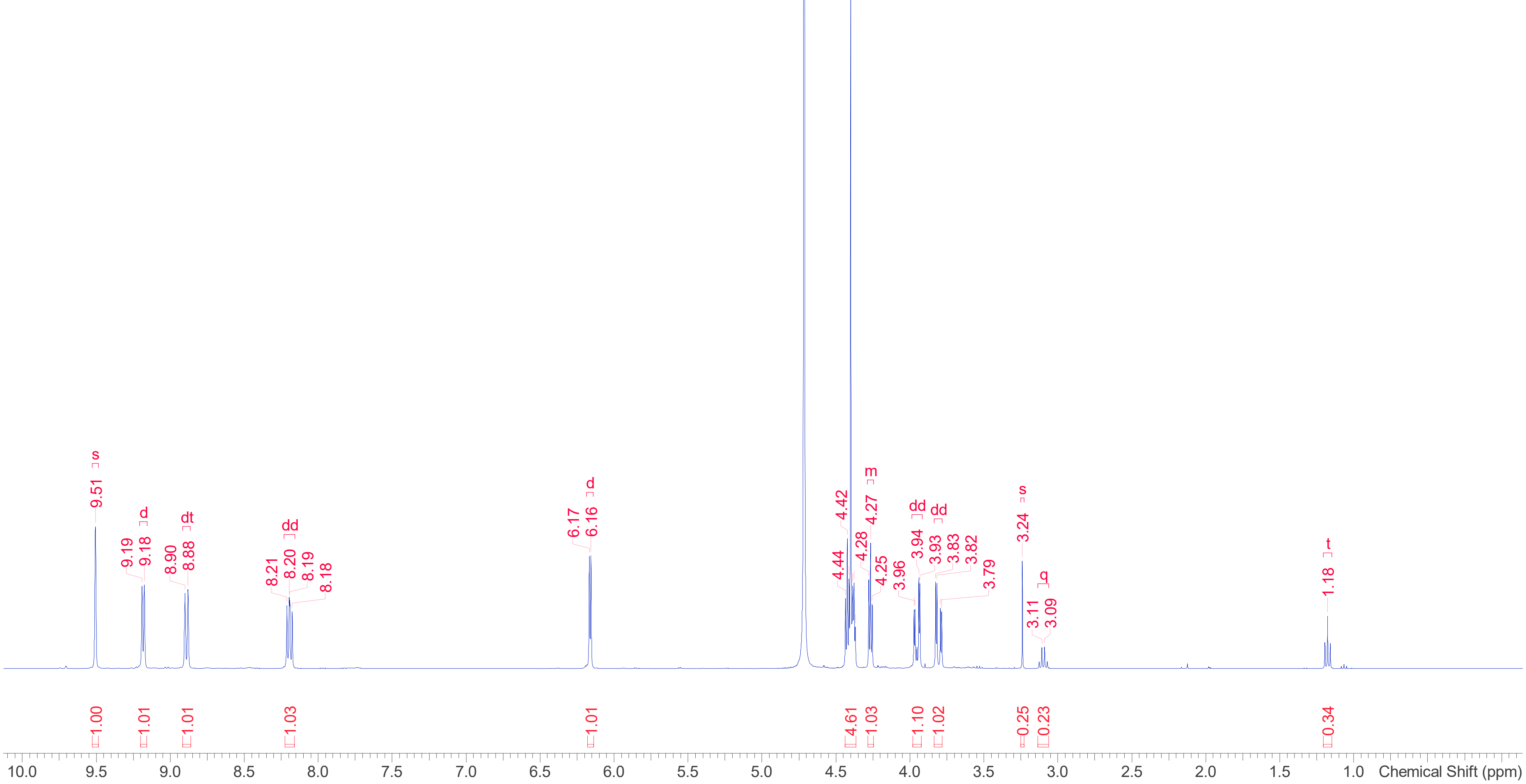

Example 15c: NR L-Hydrogentartrate ^{13}C-NMR (D_2O, 100 MHz)

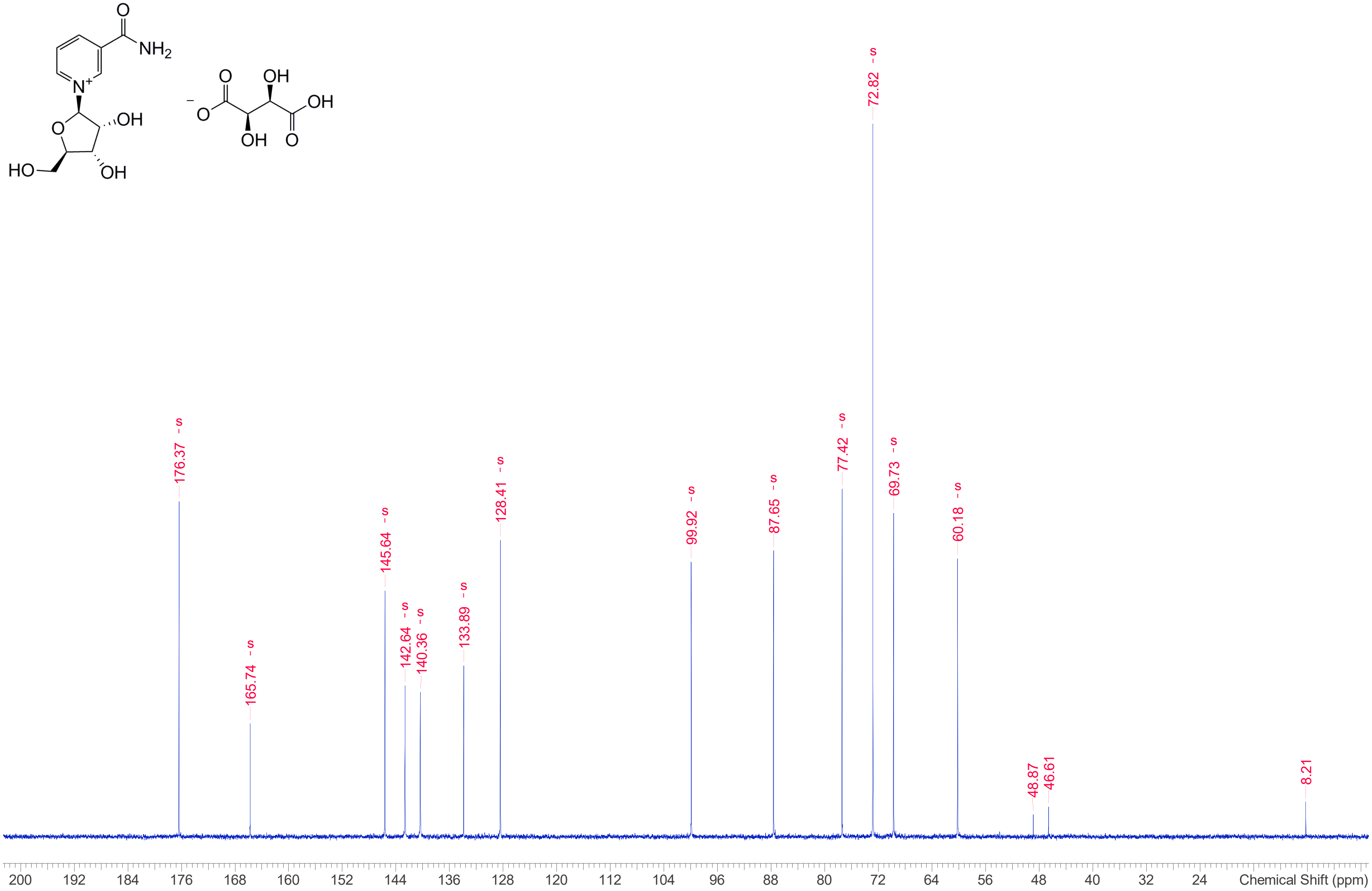

Example 16a: NR L-Hydrogenmalate ^{1}H-NMR (D_2O, 400 MHz)

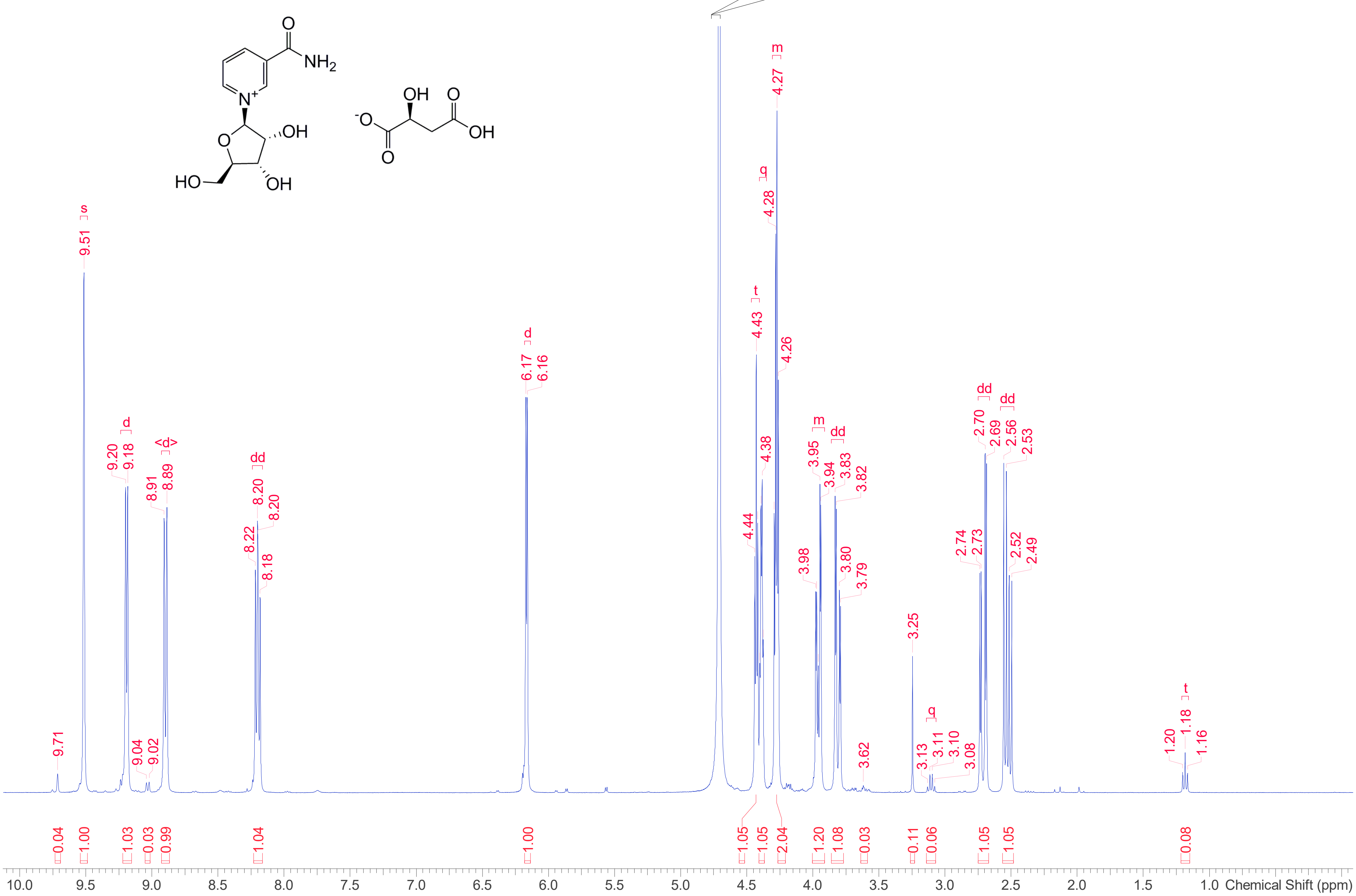

Example 16a: NR L-Hydrogenmalate ^{13}C-NMR (D_2O, 100 MHz)

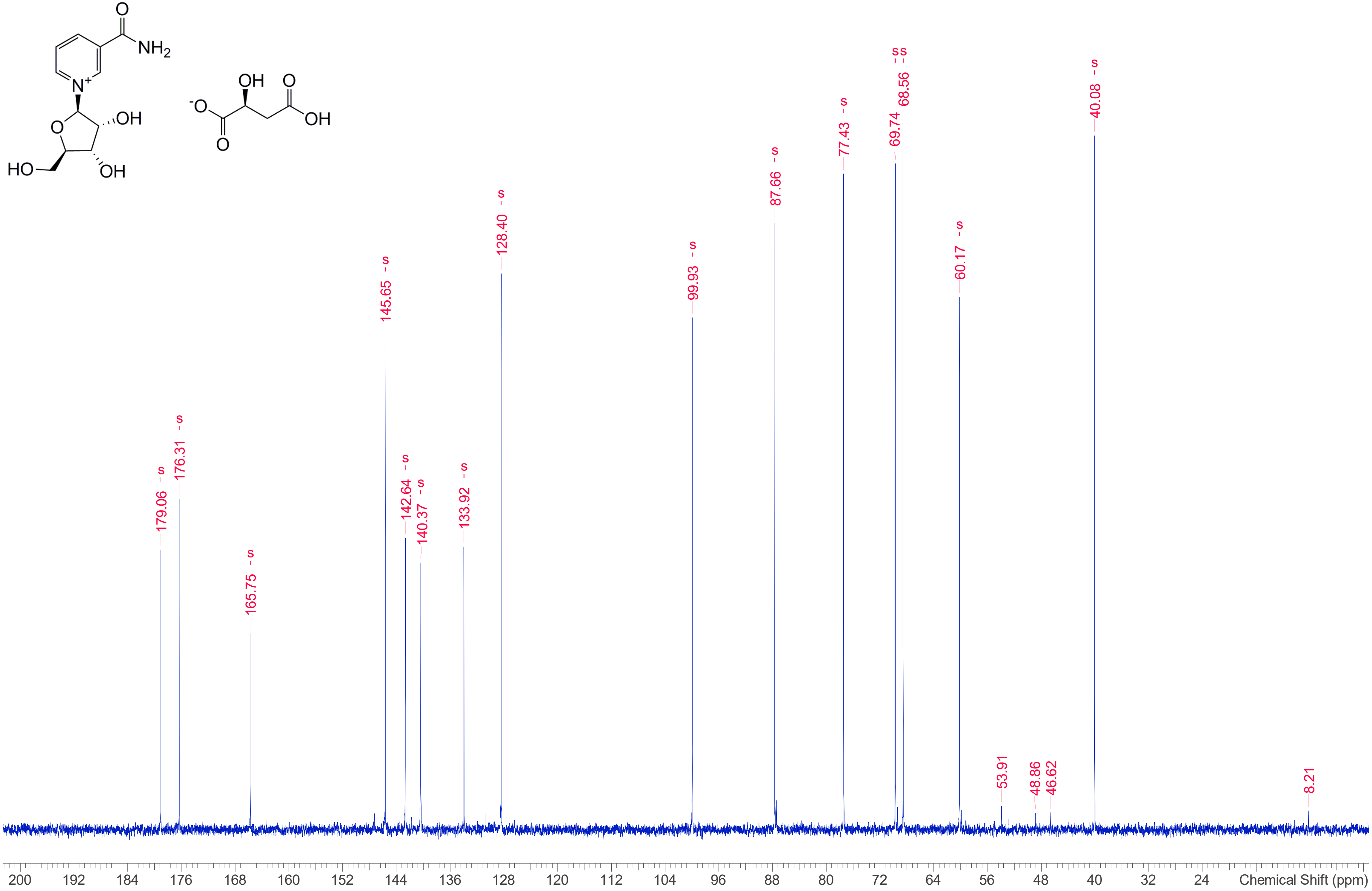

Example 16b: NR L-Hydrogenmalate ^{1}H-NMR (D_2O, 400 MHz)

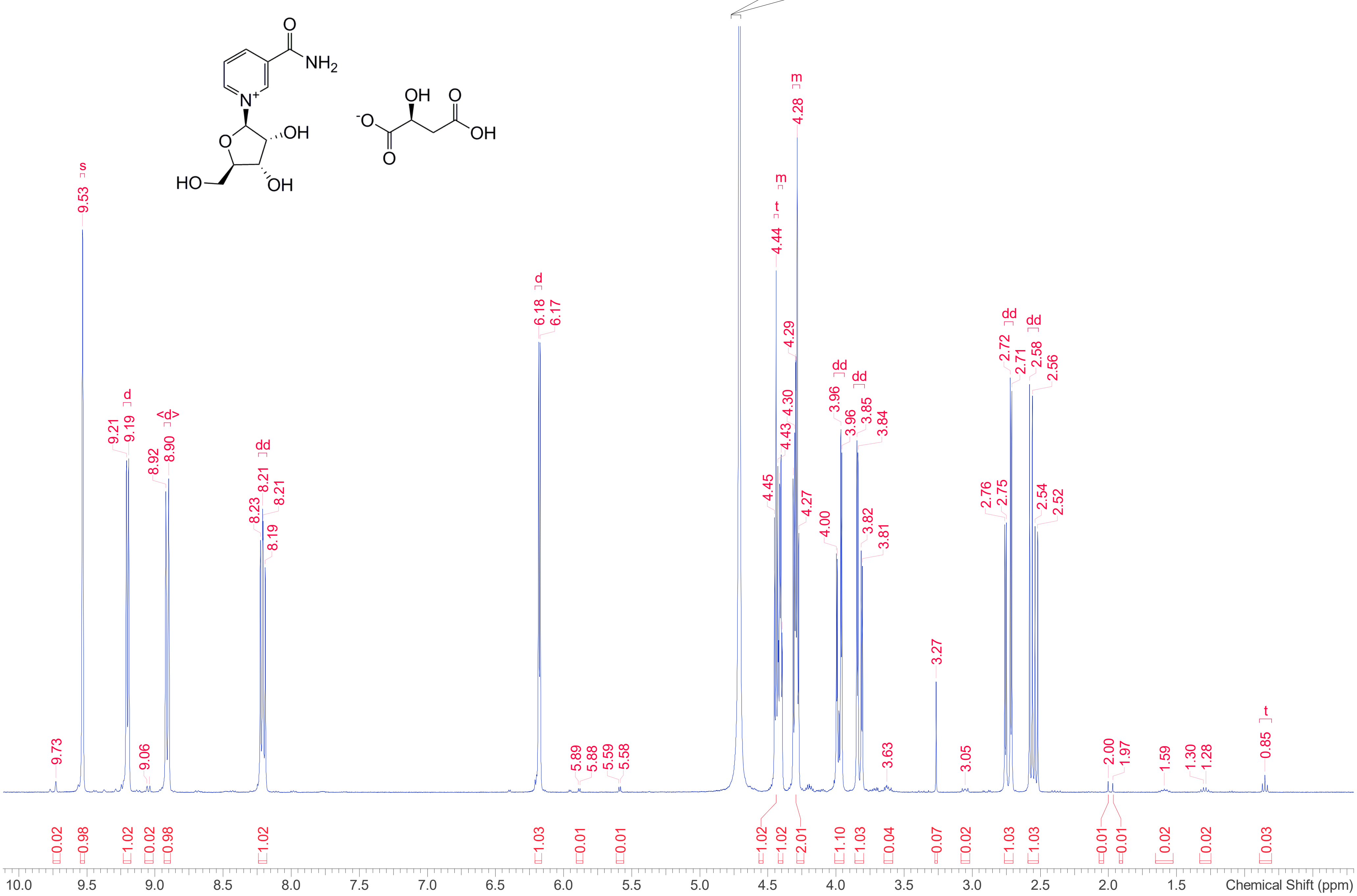

Example 16b: NR L-Hydrogenmalate ^{13}C-NMR (D_2O, 100 MHz)

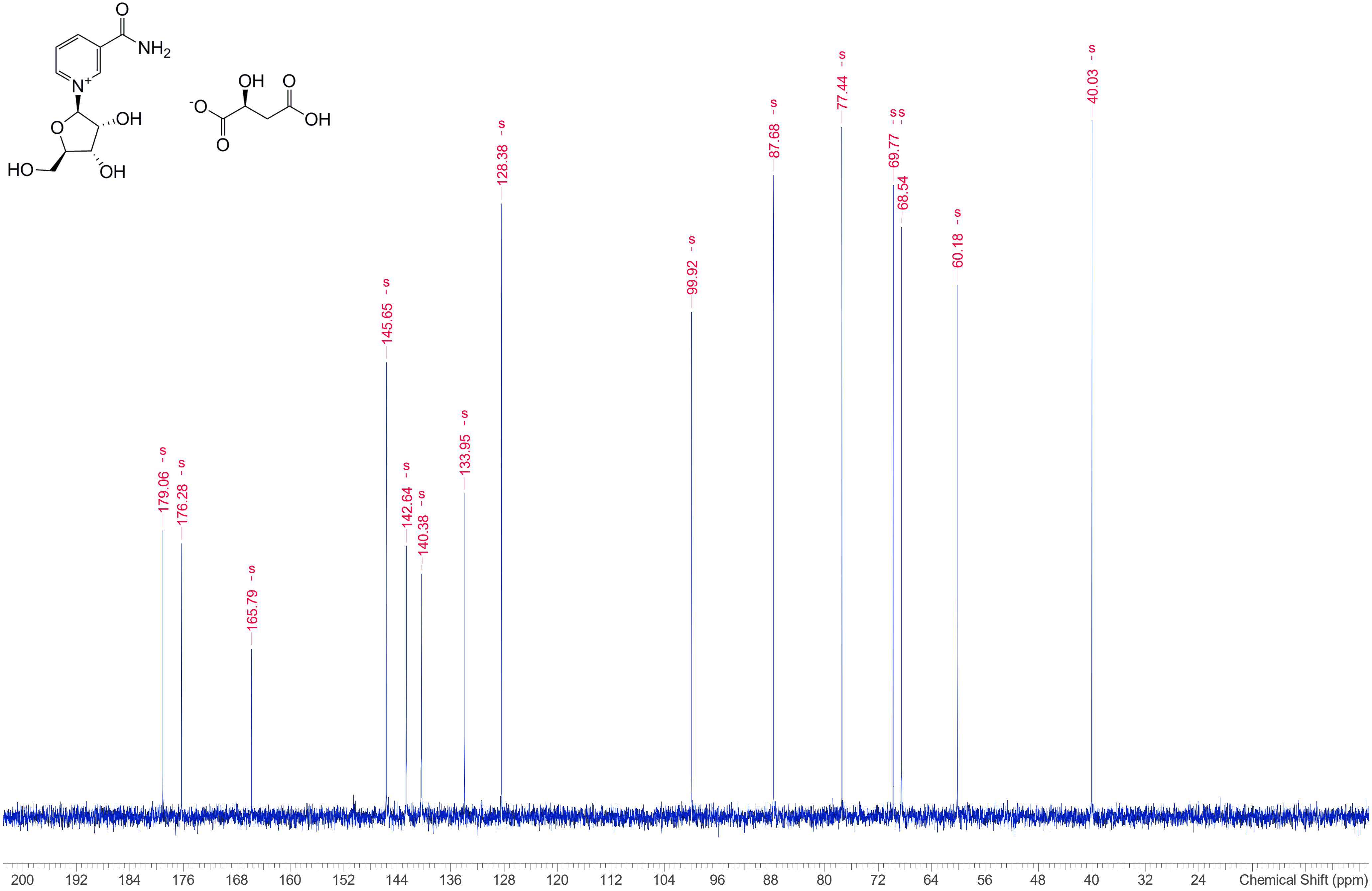

Example 17: NR-2,3,5-triacetate iodide ^{1}H-NMR (D_2O, 400 MHz)

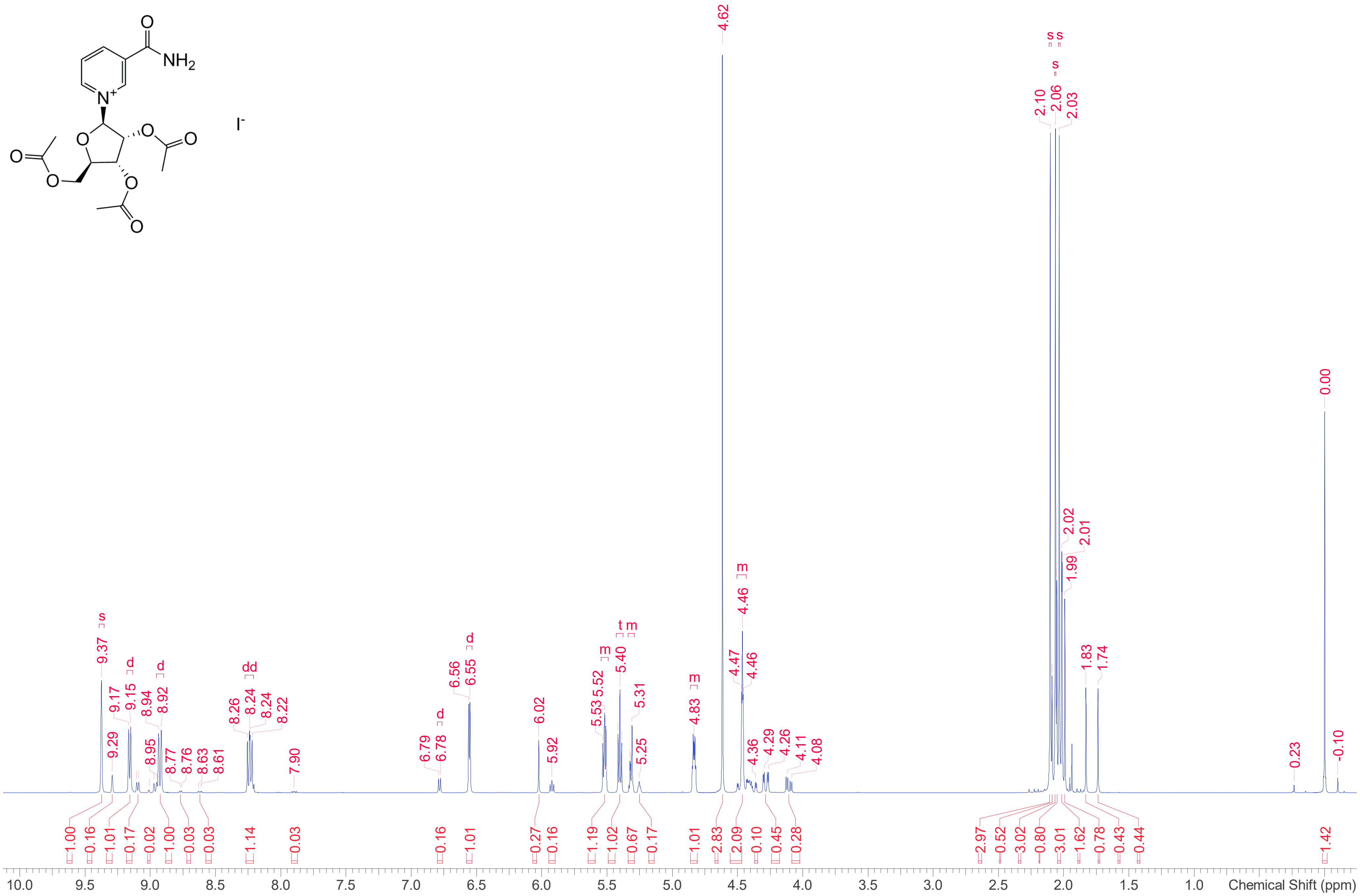

Example 17: NR-2,3,5-triacetate iodide ^{13}C-NMR (D_2O, 100 MHz)

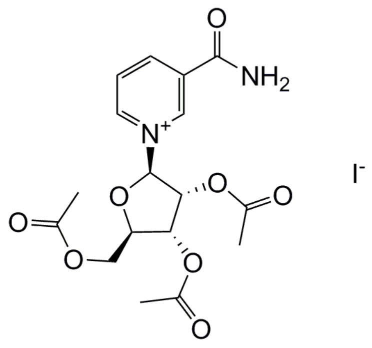

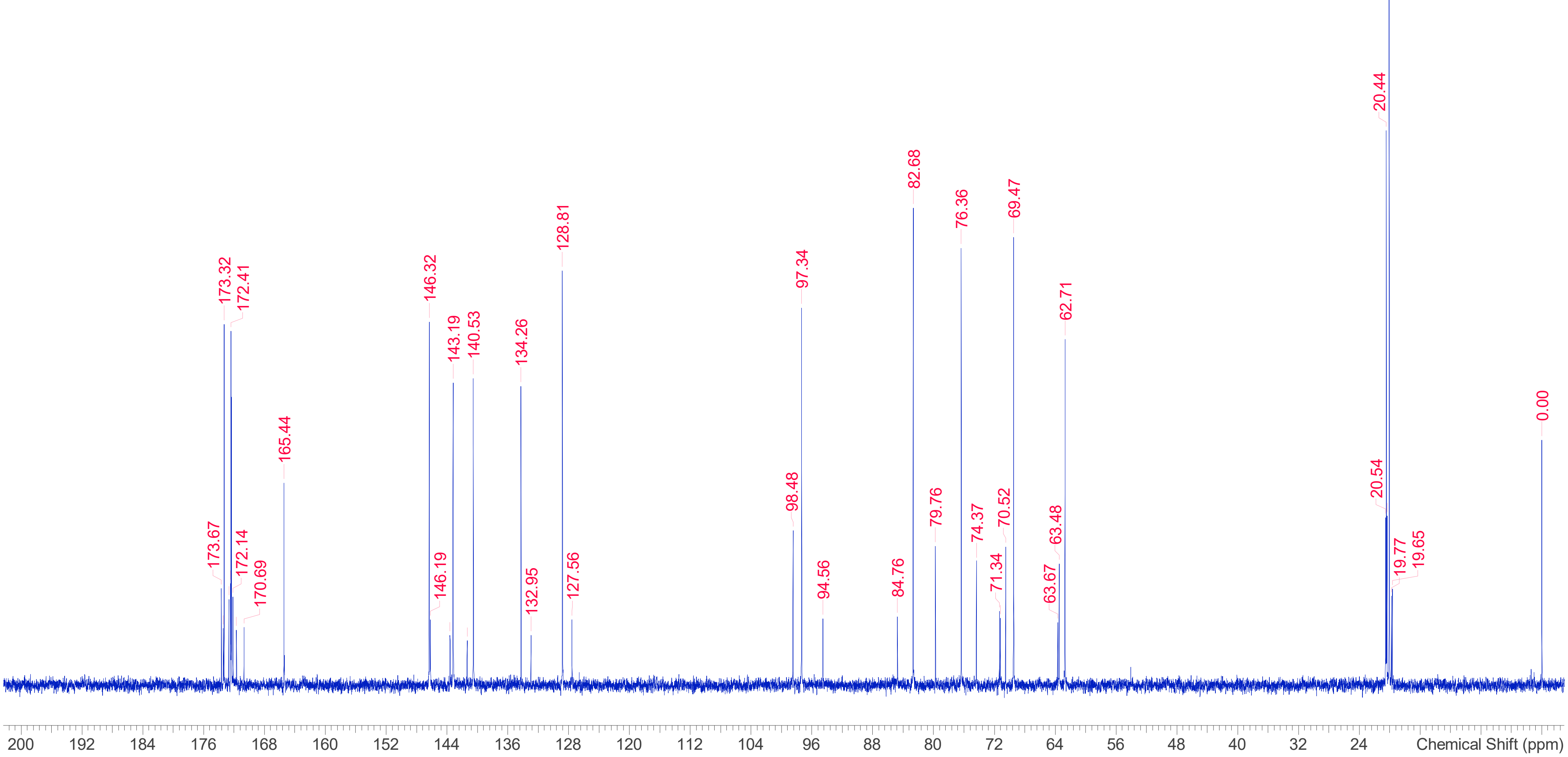

Supplement: Supplementary file 1 [file molecules-26-02729-s001.zip › molecules-1173671-supplementary/NR_Publication_NMR_Ex_V3.pdf]
